# Precise, fast and comprehensive analysis of intact glycopeptides and modified glycans with pGlyco3

**DOI:** 10.1038/s41592-021-01306-0

**Published:** 2021-11-25

**Authors:** Wen-Feng Zeng, Wei-Qian Cao, Ming-Qi Liu, Si-Min He, Peng-Yuan Yang

**Affiliations:** 1 Key Laboratory of Intelligent Information Processing of Chinese Academy of Sciences (CAS), Institute of Computing Technology, CAS, Beijing, China; 2 University of Chinese Academy of Sciences, Beijing, China; 3 Shanghai Fifth People’s Hospital and Institutes of Biomedical Sciences, Fudan University, Shanghai, China; 4 NHC Key Laboratory of Glycoconjugates Research, Fudan University, Shanghai, China; 5 The Shanghai Key Laboratory of Medical Epigenetics and the International Co-laboratory of Medical Epigenetics and Metabolism, Ministry of Science and Technology, Fudan University, Shanghai, China; 6 Department of Chemistry, Fudan University, Shanghai, China; 7 Present Address: Proteomics and Signal Transduction, Max Planck Institute of Biochemistry, Martinsried, Germany

**Keywords:** Proteome informatics, Glycobiology, Proteomics, Software

## Abstract

Great advances have been made in mass spectrometric data interpretation for intact glycopeptide analysis. However, accurate identification of intact glycopeptides and modified saccharide units at the site-specific level and with fast speed remains challenging. Here, we present a glycan-first glycopeptide search engine, pGlyco3, to comprehensively analyze intact N- and O-glycopeptides, including glycopeptides with modified saccharide units. A glycan ion-indexing algorithm developed for glycan-first search makes pGlyco3 5–40 times faster than other glycoproteomic search engines without decreasing accuracy or sensitivity. By combining electron-based dissociation spectra, pGlyco3 integrates a dynamic programming-based algorithm termed pGlycoSite for site-specific glycan localization. Our evaluation shows that the site-specific glycan localization probabilities estimated by pGlycoSite are suitable to localize site-specific glycans. With pGlyco3, we confidently identified N-glycopeptides and O-mannose glycopeptides that were extensively modified by ammonia adducts in yeast samples. The freely available pGlyco3 is an accurate and flexible tool that can be used to identify glycopeptides and modified saccharide units.

## Abstract (web summary)

pGlyco3 is a glycan-first glycopeptide search engine for the identification and localization of site-specific N- and O-glycopeptides, including glycopeptides with modified glycans.

## Main

Protein glycosylation is a fundamental posttranslational modification (PTM) that is involved in many biological functions^1–3^. In recent years, tandem mass spectrometry (MS/MS) has been shown to be a promising technique to analyze site-specific glycans on proteins^4,5^. Modern MS instruments have integrated different fragmentation techniques for glycopeptide analysis, such as higher-energy collisional dissociation (HCD), electron-transfer dissociation (ETD), electron-transfer/higher-energy collision dissociation (EThcD) and electron-transfer/collision-induced dissociation (ETciD)^6,7^. HCD, especially stepped collision energy HCD (sceHCD), can provide abundant glycopeptide Y ions (glycan Y ions with an intact peptide attached) and quite a few b/y ions of naked peptides to identify the glycan parts and peptide parts, respectively^8,9^. Information about Y ions allows us to directly identify the entire glycan composition, and core Y ions (for example, Y0, Y1 and Y2) can be used to determine the glycan and peptide masses. The b/y ions and b/y+HexNAc ions in HCD can be used to identify peptide parts, but they commonly do not provide enough information to determine site-specific glycans for multiple glycosylated sites with a given sceHCD spectrum. ETxxD (ETD, EThcD and ETciD) can generate glycan-attached c/z ions to not only identify peptides but also deduce site-specific glycans^10–13^.

Many glycopeptide search engines based on modern MS techniques have been developed over the last decade^4,5,14^. There are three main search strategies for intact glycopeptide identification: peptide-first, glycan-removal and glycan-first. The peptide-first search is arguably the strategy that is most widely used and was adopted by Byonic^15^, gpFinder^16^, GPQuest^17^, pMatchGlyco^18^, GPSeeker^19^, Protein Prospector^11^ and two recently developed tools MSFragger (MSFragger-Glyco^20^) and MetaMorpheus (MetaMorpheus O-Pair^21^). This method first searches the peptide part and then deduces the glycan part as a large variable modification by considering some B/Y ions. MSFragger and MetaMorpheus use the peptide ion-indexing technique^22^ to accelerate the peptide-first search. The glycan-removal search deduces the pseudopeptide masses from N-glycopeptide spectra by using potential reducing-end Y ions, and then modifies the spectral precursor masses as the pseudopeptide masses to identify peptides using conventional peptide search engines^23–25^. Based on the deduced glycan mass, MAGIC^25^ searches the glycan compositions by using the knapsack algorithm without glycan composition/structure databases. The recently developed O-search further extends the glycan-removal strategy for O-glycopeptide identification^26^. These tools, especially Byonic, MSFragger and MetaMorpheus, have increased the identification sensitivity for glycoproteomics^27^; however, glycan-level quality control has not been a priority. Recently published StrucGP also first identifies the peptide parts based on trimannosyl core ions of the N-glycans^28^. Unlike MAGIC, it tries to interpret glycan structures based on predefined substructure templates without the need for any glycan database, and it also includes glycan-level quality control after glycan identification. Glycan-first search is used in the pGlyco software series^8,29^ as well as other tools (Sweet-Heart^30^ and GlycoMaster DB^31^), and it first searches the glycan parts to remove unreliable glycans and then searches the peptide parts. pGlyco 2.0 is the first search engine that can perform glycan-, peptide- and glycopeptide-level quality control for glycopeptides. It was extended for peptide identification and tandem mass tag (TMT)-quantification with MS3 by SugarQuant^32^. However, pGlyco 2.0 supports only the search against the normal mammalian N-glycans present in GlycomeDB^33^ with sceHCD spectra; hence, it is difficult for users to apply it to analyze customized glycans or modified saccharide units (for example, phospho-Hex or mannose-6-phosphate). Furthermore, modern glycopeptide search engines should consider site-specific glycan localization (SSGL), as ETxxD techniques have been used widely in glycoproteomics. Some works, such as the ‘Delta Mod score’ of Byonic and ‘SLIP’^34^ of Protein Prospector, extended the site localization algorithms from traditional PTMs to glycosylation, and assessed the localization reliabilities. However, traditional PTM search algorithms do not address the computational complexity of SSGL for glycopeptides, which has been well discussed by Lu et al.^21^ Graph-based SSGL algorithms, such as GlycoMID for hydroxylysine O-glycosylation^35^ and MetaMorpheus for common O-glycosylation^21^, enable a fast localization process. However, accurate SSGL and its validation are still unresolved problems.

Here, we propose pGlyco3, a glycopeptide search engine that enables analysis of modified saccharide units and SSGL. pGlyco3 applies the glycan-first search strategy to accurately identify glycopeptides. It uses canonicalization-based glycan databases to support the modified saccharide unit analysis and implements a glycan ion-indexing technique to accelerate the search for glycans. For SSGL, we developed a dynamic programming algorithm termed pGlycoSite to efficiently localize site-specific glycans using ETxxD spectra. We emphasized validation for glycopeptide identification and SSGL. To validate the accuracy of pGlyco3, we designed several experiments to show that pGlyco3 outperforms other tools in terms of identification accuracies for both N- and O-glycopeptides, especially at the glycan level. We also designed four methods to validate the SSGL of pGlycoSite. Our validation shows that SSGL probabilities estimated by pGlycoSite are suitable to localize site-specific glycans. We used pGlyco3 to identify a modification on Hex (Hex with an ammonia adduct, simplified as ‘aH’) on N-glycopeptides and O-mannose (O-Man) glycopeptides in yeast samples. We validated the aH search results on yeast with N-glycome data and ^15^N-/^13^C-labeled glycopeptide data. This analysis further demonstrates the reliability and flexibility of pGlyco3 for intact glycopeptide and modified saccharide unit identification.

## Results

### Workflow of pGlyco3

**Fig. 1:**
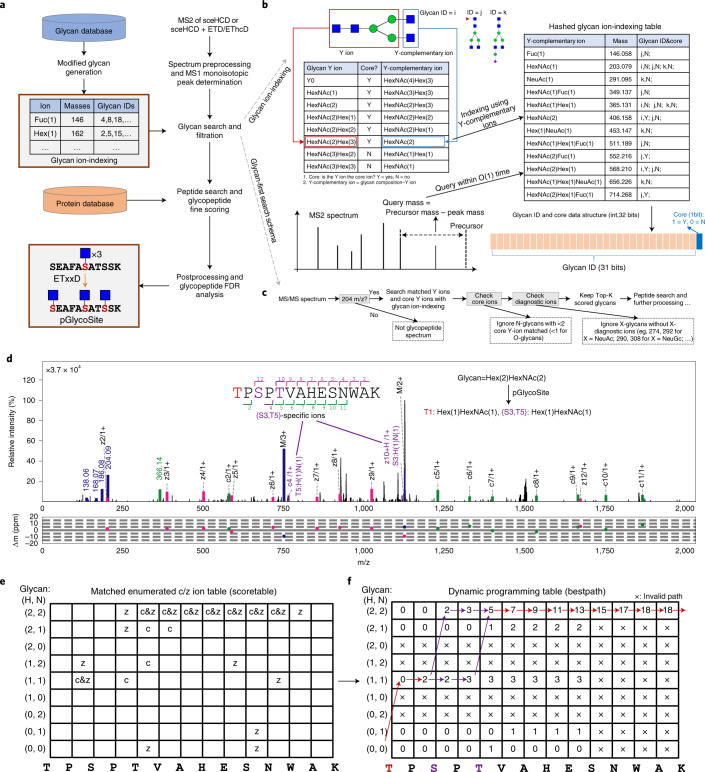
Workflow of pGlyco3 and its algorithms. **a**, Software schema. **b**, Illustration of the glycan ion-indexing technique. More glycan ion-indexing information is illustrated in Supplementary Note 2. **c**, Glycan ion-indexing-based glycan-first search schema of pGlyco3. **d**–**f**, Representative example of pGlycoSite, showing an EThcD-GPSM (‘TPSPTVAHESNWAK + H(2)N(2)’, H = Hex, N = HexNAc) with localized sites using the pGlycoSite algorithm (**d**), all possible matched c/z ions against the EThcD spectrum (ScoreTable) (**e**) and the dynamic programming table from bottom left to top right (BestPath) (**f**). The arrows indicate the best-scored paths, and the purple lines show that two paths share the same score from S3 to T5. T1 is uniquely localized with Hex(1)HexNAc(1) and {S3:T5} is localized as a ‘site-group’ with Hex(1)HexNAc(1), as shown in **d**. Details of the calculation of the ScoreTable and BestPath are illustrated in Methods and Supplementary Note 3.

pGlyco3 uses sceHCD and ETxxD spectra to identify glycopeptides, analyze modified saccharide units, estimate glycan/peptide false discovery rates (FDRs) and localize site-specific glycans (Fig. 1a). For HCD-pd-ETxxD spectra, pGlyco3 merges HCD and ETxxD spectra before searching. The detailed workflow is described in the Methods. For glycan part identification, pGlyco3 provides several built-in N- and O-glycan databases, and can generate new glycan databases from GlycoWorkbench^36^ with expert knowledge. Benefitting from the flexible canonicalization-based glycan representation, pGlyco3 enables the convenient analysis of modified or labeled glycans in glycopeptides (Methods and Supplementary Note 1).

In contrast to Byonic, MetaMorpheus and MSFragger, pGlyco3 applies the glycan-first strategy as it first searches the glycan parts and filters out unreliable ones. For a fast glycan-first search, we designed a glycan ion-indexing technique to score all glycans as well as their core Y ions (core Y ions are defined in Supplementary Table 2) by matching the query spectrum only once within the linear search time (that is, O(#peaks); Supplementary Note 2). The key observation of glycan ion-indexing is that if a peak is a Y ion of a glycopeptide, then the precursor mass minus the peak mass would be the Y-complementary ion mass (Methods). As the Y-complementary ion mass does not contain the peptide mass, it enables us to search the glycans before peptides are identified. Therefore, the ion-indexing of glycans in pGlyco3 indexes Y-complementary masses instead of Y ions, enabling the fast glycan-first search (Fig. 1b, Methods and Supplementary Note 2). As shown in Fig. 1b, the hashed keys of the glycan ion-indexing table are the Y-complementary ion masses, and the values are the list of glycan identification numbers (IDs) from which the Y-complementary ions originate. As the core Y ions are very important in glycan identification, we use an extra bit in the glycan ID of the indexing table to indicate whether the corresponding Y ion of the Y-complementary ion is a core ion. Note that the Y0 ion is always considered as a core ion for an N- or O-glycopeptide in pGlyco3. The schema of the glycan-first search is shown in Fig. 1c. This method applies a few verification steps to ensure the reliability of the remaining glycans, including the number of matched core Y ions, the existence of glycospecific diagnostic ions and the rank of glycan scores. After glycan filtration, pGlyco3 performs the peptide search, glycan/peptide fine-scoring, postsearch processing and glycopeptide FDR estimation (Fig. 1a).

pGlyco3 integrates an algorithm termed pGlycoSite to localize the site-specific glycans and estimate the localization probabilities using c/z ions in ETxxD spectra. For a given spectrum with the identified peptide and the glycan composition, the complexity of enumerating all possible glycopeptide forms will exponentially increase as the number of candidate sites and monosaccharides increase (Methods and Supplementary Note 3). Instead of generating the glycopeptide forms, pGlycoSite enumerates a table of all possible c/z ions, matches the table against the ETxxD spectrum. Although the number of glycopeptide forms may be exponentially large, there are only *F* × (*L* − 1) possible c or z ions, where *F* is the number of subglycan compositions and *L* is the peptide length. The pGlycoSite algorithm is extremely fast, as its computational complexity is *O*(*L* × *F*^2^) (Methods). Figure 1d–f show an example of how pGlycoSite works. Based on the matched c/z ion table (ScoreTable in Fig. 1e), pGlycoSite uses a dynamic programming algorithm to obtain the best-scored path across the table from bottom left to top right. If multiple paths reach the same best score, pGlycoSite will regard the amino acids from the branching position to the merging position as a ‘site-group’. As shown in Fig. 3f, T1 with Hex(1)HexNAc(1) is uniquely localized, and {S3:T5} is a ‘site-group’ because either S3 or T5 has a supporting site-specific c/z ion (Fig. 3d), resulting in the same score. More details of the pGlycoSite algorithm, including calculation of the ScoreTable, BestPath and localization probability estimation, are illustrated in the Methods and Supplementary Note 3. After the SSGL probabilities are estimated, the SSGL-FDR can be deduced and used to validate the accuracies of the estimated SSGL probabilities (Methods).

### pGlyco3 for N-glycopeptide identification

To demonstrate the performance of pGlyco3, we compared pGlyco3 with Byonic, MetaMorpheus and MSFragger using two N-glycopeptide datasets.

**Fig. 2:**
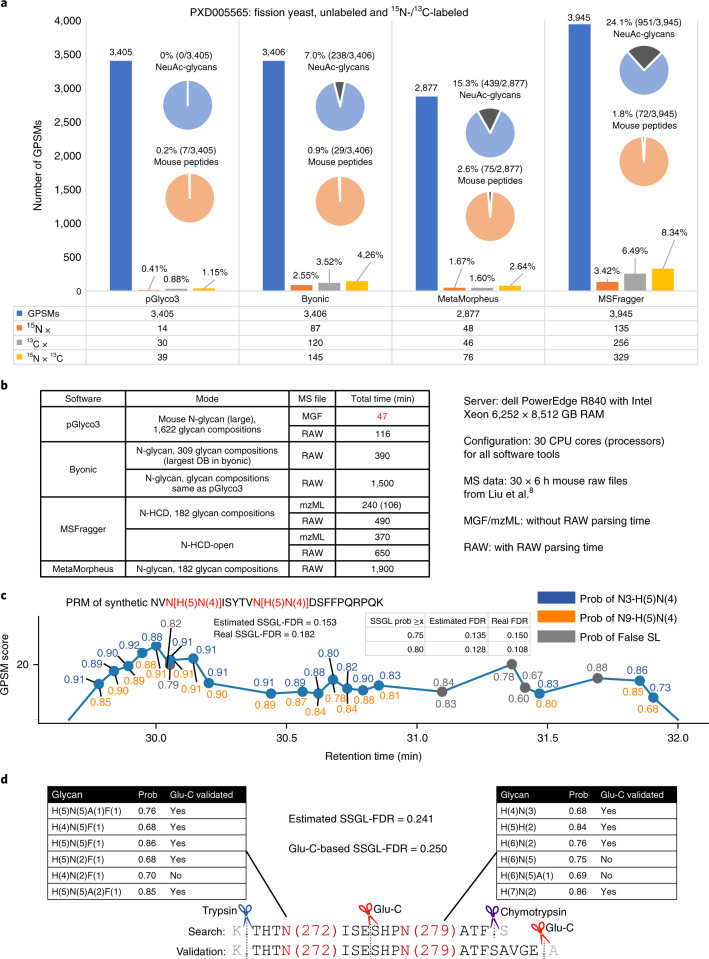
Analyzing N-glycopeptides using pGlyco3. **a**, Accuracy comparison using ^15^N-/^13^C-labeled fission yeast data (PXD005565). The element-level error rate (incorrect number of N or C elements) of the identified glycopeptides was tested via the ^15^N-/^13^C-labeled precursor signals. The potential glycan-level error rate was tested by the percentage of NeuAc-containing GPSMs and the potential peptide-level error rate was tested via GPSMs with mouse peptides. pGlyco3 shows the best accuracies at all three levels. **b**, Search speed comparison under N-glycopeptide data of five mouse tissues (Liu et al.^8^, 30 RAW files in total). The algorithm part (searching mascot generic format (MGF) files) of pGlyco3 is 5-40 times faster than that of the other tools, even when using larger glycan databases. MSFragger kernel takes only 106 min, but its postprocessing modules use too much time. **c**, Validation of pGlycoSite using the PRM of the synthetic double-site N-glycopeptide ‘NVN[H(5)N(4)]ISYTVN[H(5)N(4)]DSFFPQRPQK.’ **d**, Validation of pGlycoSite using double-site N-glycopeptides ‘K.THTN(272)ISESHPN(279)ATF.S’ of IGHM digested with chymotrypsin and trypsin. The SSGL results were validated using Glu-C and trypsin digestion with PRM. The example spectra are annotated in Supplementary Fig. 10. All annotated GPSMs for these 12 localized glycans are shown in Supplementary Data.

To compare the precision of identified glycopeptides, we used our previously published data of unlabeled, ^15^N-labeled and ^13^C-labeled fission yeast mixture samples (PXD005565 (ref. ^8^)) to test these software tools. The searched protein database was a concatenated proteome database of fission yeast and mouse, and the searched N-glycan database contained NeuAc-glycans that should not be identified in yeast samples. The search details are listed in the Methods and Supplementary Data. We analyzed three levels of identification errors. (1) The element-level error; the unlabeled glycopeptide-spectrum matches (GPSMs) may be identified with incorrect numbers of N or C elements if the GPSMs could not be verified by ^15^N- or ^13^C-labeled MS1 precursors. (2) The glycan-level error; the glycan parts tend to be incorrectly identified if the GPSMs contain NeuAc-glycans. (3) The peptide-level error; the peptide parts are false positives if they are from the mouse protein database. The testing results are shown in Fig. 2a. All tools showed low peptide-level error rates, implying that both peptide- and glycan-first searches are accurate at identifying the peptide parts of glycopeptides. However, the peptide-first-based tools showed high glycan-level error rates for yeast glycopeptides. MSFragger yielded the most identified GPSMs, but 24.1% contained NeuAc-glycans. The percentages of NeuAc-glycans obtained by MetaMorpheus and Byonic were 7.0% and 15.3%, respectively. On the other hand, benefitting from the essential glycan ion analyses and glycan FDR estimation, pGlyco3 showed good performance in controlling element-, glycan- and peptide-level error rates without losing the number of identified GPSMs. This does not mean that pGlyco3 is 100% accurate at the glycan level even if there are no NeuAc-identifications. With different glycan databases, the glycan-level error rate of pGlyco3 would be 0.8-4% (Supplementary Note 5), but it was still much better than the others. This comparison does not imply that the peptide-first strategy is not accurate. Instead, it suggests that glycan-level quality control is also necessary for the peptide-first search. pGlyco3 also showed good performance for glycopeptide identification on HCD-pd-EThcD spectra, as shown in Supplementary Fig. 7.

Next, we compared the runtime of pGlyco3 with that of other software tools on large-scale mouse N-glycopeptide data from our previous work^8^ (Supplementary Data). All these tools, including pGlyco3, use multiprocessors to accelerate the searches. We used 30 processors for all the tools to search the data on a Dell workstation with 64 central processing unit (CPU) cores and 512 gigabyte (GB) physical memories. The time comparisons are shown in Fig. 2b. Ignoring the RAW file parsing time (searching from mascot generic format (MGF) files), pGlyco3 took only 47 min to finish the search (~1.6 min per file, 1,622 glycan compositions in the database). The second-fastest tool, MSFragger, took 240 min (~8 min per file; 106 min for MSFragger kernel excluding its postprocessing modules, 3.53 min per file) with a glycan database that was seven times smaller (182 glycan compositions). The running time of pGlyco3 starting from RAW files was ~3.9 min per file, which was also faster than that of the other tools. The runtime comparisons provided strong evidence showing that the glycan-first search with glycan ion-indexing is very fast for glycopeptide identification.

Finally, we validated pGlycoSite on benchmarked double-site N-glycopeptides. We first synthesized an N-glycopeptide ‘NVN[H(5)N(4)]ISYTVN[H(5)N(4)]DSFFPQRPQK’ and used parallel reaction monitoring (PRM) to trigger its HCD and EThcD spectra. After identification and localization, we compared the SSGL results with synthetic templates to evaluate the accuracy of SSGL-FDR. As shown in Fig. 2e, among all the SSGL results, the estimated SSGL-FDR was 0.153, which was close to the real SSGL-FDR (0.182), indicating that the SSGL probabilities estimated by pGlycoSite did not have a large deviation. We also used pGlyco3 to identify and localize the double-site N-glycopeptide ‘K.THTN(272)ISESHPN(279)ATF.S’ of IGHM digested from chymotrypsin and trypsin and validated the localized double-site glycans by using Glu-C digestion and PRM (Fig. 2f). pGlyco3 localized 12 site-specific N-glycans on N272 and N279, with an estimated SSGL-FDR 0.241. Of these 12 N-glycans, 9 could be confirmed by further Glu-C digestion, showing a Glu-C-suggested SSGL-FDR of 0.250.

Overall, on the basis of glycan-first search and glycan-level quality control, pGlyco3 outperformed the other three software tools in terms of accuracy and search speed. Validation results on double-site N-glycopeptides showed that SSGL probabilities estimated by pGlycoSite were quite accurate.

### pGlyco3 for O-glycopeptide identification

**Fig. 3:**
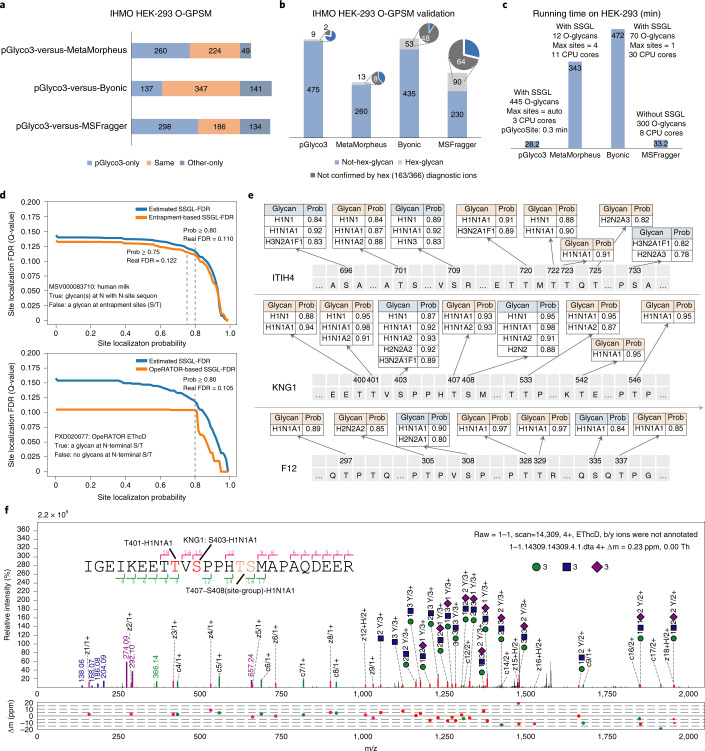
O-glycopeptide identification and SSGL of pGlyco3. **a**–**c**, Software comparisons of O-glycopeptide searches with IHMO HEK-293 cell line data. **a**, The overlaps of other tools with pGlyco3 on O-GPSMs. For glycans, only the total glycan compositions were compared; SSGL was not considered. ETD scans in the results of Byonic were mapped to their corresponding HCD scans for the comparisons. If an HCD spectrum and its sister ETD spectrum were identified as the same glycopeptide, we kept only one GPSM; otherwise, we kept both. **b**, The identified IHMO O-GPSMs were validated by Hex-containing results and further validated by Hex-diagnostic ions. **c**, The runtime comparison. **d**, Validation of the SSGL-FDR of pGlycoSite using the entrapment-based SSGL-FDR and OpeRATOR-based SSGL-FDR (Methods). **e**, Localized site-specific O-glycans of ITIH4, KNG1 and F12 proteins in human serum samples. Site-groups were discarded and SSGL assignments with maximal probability ≥0.75 are displayed. **f**, An annotated spectrum of the localized O-glycan and its SSGL probability for KNG1-S403. The HCD spectrum is annotated in Supplementary Fig. 11. The ScoreTable with BestPath is shown in Supplementary Data.

As the glycan-first search of pGlyco3 has been shown to be accurate and fast for N-glycopeptide identification, we then demonstrated the performance of pGlyco3 for O-glycopeptide identification. The workflow of O-glycopeptide is similar to that of N-glycopeptide, except that S/T are the candidate glycosylation sites, and the core Y ions are changed (Supplementary Table 2). We compared O-glycopeptide identification with pGlyco3 with tools using inhibitor-initiated homogenous mucin-type O-glycosylation (IHMO) cell line datasets (Fig. 3a–c and Supplementary Note 4). IHMO cells were almost inhibited with only truncated HexNAc(1) or HexNAc(1)NeuAc(1) O-glycans on the peptides (Supplementary Note 4). The identified O-GPSMs on the IHMO HEK-293 dataset (Fig. 3a) were then evaluated by Hex-containing GPSMs, which were further confirmed by checking the Hex-diagnostic ions (163.060 and 366.139 m/z; Fig. 3b and Methods). pGlyco3 obtained only 1.9% (9 of 484) of Hex-GPSMs, and only two of nine Hex-GPSMs could not be validated by the Hex-diagnostic ions, showing a very low Hex-suggested glycan-level error rate (2/484 ≈ 0.4%) for the IHMO HEK-293 data. The Hex-suggested glycan-level error rates of the three other software tools on the same dataset were: ~2.9% (8/273) for MetaMorpheus, ~10.0% (48/488) for Byonic and ~20.0% (64/320) for MSFragger. These results further demonstrate the accuracy of pGlyco3 in glycopeptide identification. The search speed of pGlyco3 was also higher than that of others on the same IHMO dataset.

To demonstrate the accuracy of pGlycoSite for SSGL on O-glycopeptides, we designed two experiments to validate the estimated SSGL-FDR: entrapment-based and OpeRATOR-based methods. The entrapment-based method applies pGlycoSite on N-GPSMs by regarding J/S/T (J is N with the N-glycosylation sequon) as the candidate sites, and SSGL would be false in an N-GPSM if a site is not localized at J. The OpeRATOR-based method suggests that SSGL would be false for a GPSM if no glycans are localized at the N-terminal S/T since the OpeRATOR recognizes O-glycans and cleaves O-glycopeptides at the N-termini of O-glycan-occupied S or T^37^. The entrapment-based SSGL-FDR and OpeRATOR-based SSGL-FDR could be deduced (Methods). SSGL-FDRs estimated by pGlycoSite were validated by entrapment-based SSGL-FDR and OpeRATOR-based SSGL-FDR, and the results showed that SSGL-FDRs estimated by pGlycoSite did not have much deviation (Fig. 3d and Supplementary Fig. 2). Coupled with the validation results of double-site N-glycopeptides (Fig. 2e–f), the entrapment-based SSGL-FDR and OpeRATOR-based SSGL-FDR (Fig. 3d) could verify the accuracies of pGlycoSite for SSGL on both N- and O-glycopeptides. Figures 2c and 3d also suggested that the real SSGL-FDR would be nearly 10% at a SSGL probability of ≥0.8, and the SSGL-FDR would be also acceptable at a SSGL probability of ≥0.75 (‘level-1’ SSGL in MetaMorpheus O-pair). Therefore, we recommend 0.8 or 0.75 as the SSGL probability cut-off value, but it should be noted that the real SSGL-FDRs may be different for different datasets at the same SSGL probability threshold.

Finally, we used pGlycoSite to analyze O-glycopeptides in human serum samples, and the localized site-specific O-glycans with probabilities for the proteins inter-alpha-trypsin inhibitor heavy chain H4 (ITIH4), kininogen-1 (KNG1) and coagulation factor XII (F12) are shown in Fig. 3e. All these localized O-glycosylation sites, except for S403 on KNG1, can be confirmed on https://www.uniprot.org/ or http://www.oglyp.org/, showing the reliability of these localized sites^38^. The O-glycosylation of S403 on KNG1 can be verified by EThcD data, as shown in Fig. 3f. Enlarged regions of the key c/z ions for SSGL of this GPSM, as well as a few other GPSMs, can be found in the Supplementary Data. It is worth mentioning that all GPSMs with ‘J’-containing peptides were removed to avoid the interference of N-glycosylation (‘J’ is ‘N’ with sequon N-X-S/T/C). Based on this dataset, inspired by the work of MetaMorpheus O-pair^21^, we also evaluated the search times and peptide FDRs using different entrapment protein databases (Supplementary Fig. 9). Results showed that pGlyco3 is fast and accurate even with a large number of entrapment peptide sequences.

### Analyses of aH-glycopeptides in yeast samples

In the previous sections, after pGlyco3 was shown to be reliable for glycopeptide identification, we applied pGlyco3 to analyze aH-glycopeptides in yeast samples. In this manuscript, ‘aH’ refers to the Hex with an ammonium adduct^39^.

**Fig. 4:**
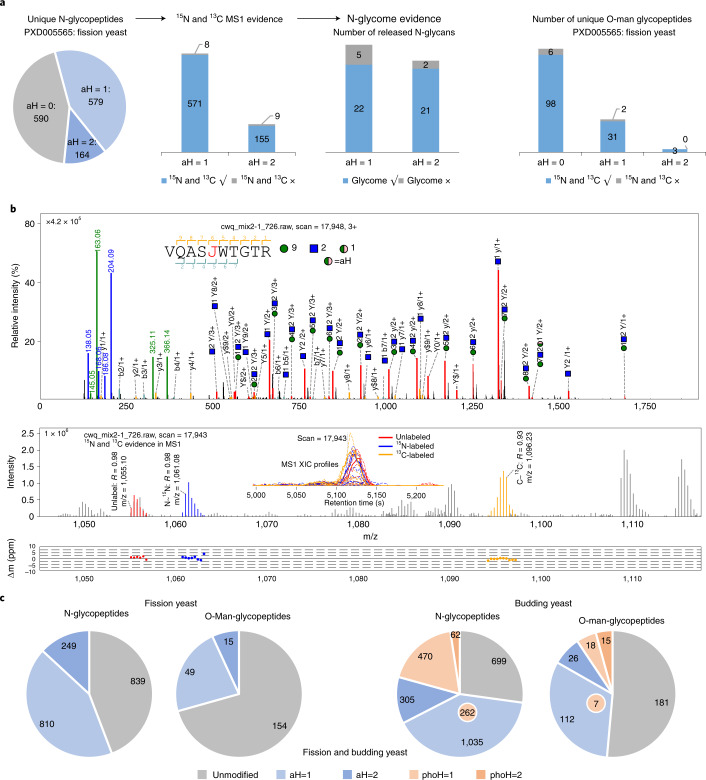
Analysis and verification of the ‘Hex+17 (aH)’. **a**, Unlabeled aH-N-glycopeptides are identified on fission yeast data (PXD005565) and verified by the ^15^N-/^13^C-labeled MS1 precursors and the N-glycome MS/MS data. Unlabeled aH-O-Man glycopeptides are also identified and verified by ^15^N-/^13^C-labeled MS1 evidence. **b**, MS/MS spectral annotation (top) and ^15^N and ^13^C MS1 evidence (bottom). In the MS1 annotation, *R* is the Pearson correlation coefficient between the theoretical and experimental isotope distributions. The blue square and green circle refer to HexNAc and Hex, respectively. The MS2 spectrum is a chimera spectrum of ‘VQASJ(N)WTGTR + Hex(9)HexNAc(12)aH(1)’ and ^15^N-labeled ‘VQASJ(N)WTGTR + Hex(10)HexNAc(2)’ (Supplementary Data). **c**, Deep analysis of aH-N-glycopeptides and aH-O-Man glycopeptides in fission yeast and budding yeast data. Phospho-Hex (phoH) glycopeptides are also found in budding yeast samples; 262 N-glycopeptides and seven O-Man glycopeptides in budding yeast contain both aH and phoH.

We first searched N-glycopeptides and O-Man glycopeptides in the ^15^N/^13^C-labeled fission yeast dataset (PXD005565)^8^ with Hex ‘modified’ by aH (Methods). Then, the identified aH-glycopeptides were confirmed at the element level by ^15^N- and ^13^C-labeled precursors. As shown in Fig. 4a, we found 579 unique aH-N-glycopeptides with one aH and 164 aH-N-glycopeptides with two aHs. In total, 571 of 579 and 155 of 164 aH-glycopeptides could be confirmed by the ^15^N and ^13^C MS1 evidence for aH×1 and aH×2, respectively. A total of 86% (22 + 21 of 50) of aH-N-glycans identified in glycopeptides could be confirmed by MS/MS data of released N-glycans (Fig. 4a). aH could also be identified on O-Man glycopeptides in PXD005565 and confirmed by ^15^N and ^13^C MS1 evidence (Fig. 4a, right).

Figure 4b and Supplementary Fig. 3d show the MS/MS, ^15^N and ^13^C MS1 and N-glycome evidence of the aH-N-glycopeptide ‘VQASJ(N)WTGTR + Hex(9)HexNAc(2)aH(1).’ This glycopeptide was identified at MS/MS scan 17,948 and was validated by its unlabeled, ^15^N-labeled and ^13^C-labeled MS1 precursors at scan 17,943. The MS/MS annotation showed that the aH-glycan was confidently identified with many continuous Y ions matched (Fig. 4b, top). MS1 precursor evidence showed high Pearson correlation coefficients (*R*) and low matching mass errors for all the unlabeled, ^15^N-labeled and ^13^C-labeled precursors (Fig. 4b, bottom).

Figures 4a,b show that pGlyco3 can confidently identify aH-glycopeptides. We then generated large-scale fission yeast and budding yeast datasets to further analyze the aH-glycopeptides (Supplementary Note 4; the search parameters are shown in Supplementary Data) and the results are shown in Fig. 4c. We found large proportions of aH-glycopeptides in both fission yeast and budding yeast samples. In total, 55.8% (1059 of 1898) of aH-N-glycopeptides and 29.4% (64 of 218) of aH-O-Man glycopeptides were identified in fission yeast; the corresponding percentages were 58.0% and 40% in budding yeast. pGlyco3 also identified several phoH-N-glycopeptides and phoH-O-Man glycopeptides in the budding yeast dataset. We analyzed site-specific N-glycans and O-Man glycans of the protein O-mannosyltransferase (ogm1) in fission yeast, and the O-Man sites were localized by pGlycoSite on EThcD data, as displayed in Supplementary Fig. 5. aH-glycopeptides were also found in other public glycopeptide datasets (Supplementary Fig. 8), demonstrating the commonality and importance of aH.

## Discussion

In this work, we emphasize the importance of glycan-level quality control for the development of glycopeptide search engines to ensure glycan-level accuracy. A glycan is not just a simple PTM, and its mass is sometimes so large that traditional PTM searches may obtain different glycan compositions or even different glycan and amino acid combinations^23^. Unexpected peptide-level modifications could also lead to incorrect glycan identifications^40^. Fortunately, glycans have their own fragment ions and diagnostic ions, allowing search engines to achieve more accurate glycan identification. Strict glycan-level quality control may reduce the sensitivity, but accuracy should be the first priority for any search engine. Therefore, even for peptide-first search engines, we recommend performing glycan-level quality control after the peptides are identified.

SSGL is important, especially for O-glycosylation. MetaMorpheus uses a graph-based algorithm to localize sites and estimate site probabilities, but it needs to build different graphs for different combinations of glycans. pGlycoSite provides a one-step algorithm to deduce the sites on the basis of the ScoreTable with a dynamic programming algorithm, making the SSGL step extremely fast. As heuristic algorithms for glycosylation SSGL have been developed only in the last few years, there is still plenty of room to improve the scoring schema, SSGL probability estimation and result validation.

pGlyco3 provides a convenient way to analyze modified saccharide units on the basis of canonicalization-based glycan representations, making the analysis of modified saccharide units similar to that of peptide modifications for users. This new feature enables us to identify aH-glycopeptides, which could be validated through N-glycome data and ^15^N/^13^C-labeled data, on N-glycopeptides and O-Man glycopeptides in yeast samples. aH may be not easy to avoid as it could also be found in many other public glycopeptide datasets.

In Supplementary Note 5, on the basis of the PXD005565 yeast dataset, we further discuss how different glycan databases influenced the identification; why Byonic obtained different glycopeptides and, more importantly, how to improve our glycopeptide identifications. The results also show the pros and cons of searching for aH during glycopeptide identification; the identification interferences caused by aH should be considered in the routine glycoproteomic analyses. We also found commonly encountered errors for different search engines (for example, precursor detection error), suggesting possible future improvements for all software tools including pGlyco3.

## Methods

### Spectrum preprocessing

pGlyco3 can process HCD and ‘HCD+ETxxD’ (HCD-pd-ETxxD, or HCD followed by ETxxD) data for N-/O-glycopeptide identification; here, ETxxD could be either ETD, EThcD or ETciD. Note that pGlyco3 is optimized for both glycan and peptide fragment analysis using sceHCD; hence, sceHCD is always recommended, as discussed in our previous work^8^. If HCD and ETxxD spectra are generated for the same precursor, pGlyco3 will automatically merge them into a single spectrum for searching. Peaks from HCD and EThcD spectra are merged on the basis of a specified mass tolerance set by the users (±20 ppm by default) in the spectrum preprocessing step. pGlyco3 then deisotopes and deconvolutes all MS2 spectra and removes the precursor ions. pGlyco3 uses pParse to determine the precursor mono ions and to export chimera spectra. The pParse module has been also used in peptide, glycopeptide and crosslinked peptide identification^41,42^. pGlyco3 filters out nonglycopeptide spectra by checking glycopeptide-diagnostic ions, then searches the glycan parts with the glycan ion-indexing technique. The diagnostic ions can be defined by the users, the default ion is 204.087 m/z for both N-glycosylation and O-glycosylation.

### Glycan-first search and glycan ion-indexing

In pGlyco3, each glycan structure is represented by a canonical string in the glycan database. pGlyco3 provides quite a few built-in N- and O-glycan databases, and it supports the conversion of the glycan structures of GlycoWorkbench^36^ into the canonical strings of pGlyco3. For modified saccharide units, pGlyco3 will automatically substitute one or several unmodified monosaccharides in each canonical string into modified forms, making it very convenient for the modified saccharide unit analysis (Supplementary Note 1).

For each peak in an MS2 spectrum, pGlyco3 assumes it is a Y ion of a glycopeptide with the peptide attached to the database search. In pGlyco 2.0, we calculated the peptide mass for each glycan in the glycan database by ‘precursor mass − glycan mass’, and theoretical Y ion masses of each glycan could be deduced and matched against the MS2 spectrum. This glycan search algorithm has to match the same spectrum for N times (O(N), N is the number of glycans in the glycan database). But pGlyco3 searches for Y-complementary ions (‘precursor mass − Y ion mass’) instead of Y ions; the key observation was that:

$${\it{\mathrm{peak}}}\,{\it{\mathrm{mass}}} = {\it{\mathrm{peptide}}}\,{\it{\mathrm{mass}}} + {\it{\mathrm{glycan}}}\,{\it{\mathrm{Y}}}\,{\it{\mathrm{ion}}}\,{\it{\mathrm{mass}}} + {\it{\mathrm{peak}}}\,{\it{\mathrm{mass}}}\,{\it{\mathrm{error}}}\left( { + {\it{\mathrm{proton}}}} \right)$$

$${\it{\mathrm{precursor}}}\,{\it{\mathrm{mass}}} = {\it{\mathrm{peptide}}}\,{\it{\mathrm{mass}}} + {\it{\mathrm{glycan}}}\,{\it{\mathrm{mass}}} + {\it{\mathrm{precursor}}}\,{\it{\mathrm{mass}}}\,{\it{\mathrm{error}}}\left( { + {\it{\mathrm{proton}}}} \right)$$

$$\begin{array}{*{20}{c}} { \Rightarrow {\mathrm{precursor}}\,{\mathrm{mass}} - {\mathrm{peak}}\,{\mathrm{mass}}} & = & {\mathrm{glycan}\,{\mathrm{mass}} - {\mathrm{glycan}}\,{\mathrm{Y}}\,{\rm{ion}}\,{\mathrm{mass}} + {\mathrm{mass}}\,{\mathrm{error}}} \\ {} & = & {\mathrm{Y-}}{{\mathrm{complementary}}\,{\mathrm{mass}} + {\rm{mass}}\,{\mathrm{error}}} \end{array}$$

To accelerate the Y-complementary ion search, pGlyco3 builds the ion-indexing table for Y-complementary ions of all possible Y ions for the glycan database (Supplementary Note 2). The Y-complementary ion composition is defined as ‘full glycan composition – Y-ion glycan composition.’ A table of all possible unique Y-complementary ion compositions is generated, and the list of glycan IDs where the Y-complementary compositions originate is also recorded in the table. For the glycan-first search, core Y ions (for example, trimannosyl core Y ions in N-glycans; Supplementary Table 2) are the key ions for N-glycan scoring. Hence, pGlyco3 encodes the glycan ID using 31 bits of the 32-bit integer and uses the extra bit to record whether the corresponding Y ion is the core ion of the glycan (Supplementary Note 2). The table is then sorted and hashed by the masses for a fast query (within the O(1) query time). As a result, it takes only O(#Peak) time to obtain the matched ion counting scores as well as the matched core ion counting scores of all glycans for every spectrum; here, #Peak refers to the number of peaks in a spectrum. pGlyco3 retains the glycans with ≥ *n* core Y ions matched (*n* = 2 for an N-glycan and n = 1 for an O-glycan). Glycans are further filtered out if they contain a specific monosaccharide but are not supported by corresponding monosaccharide-diagnostic ions (Supplementary Table 1). Finally, pGlyco3 retains the top 100 candidate glycan compositions (sum of the ion counting score and the core ion counting score) for the peptide search. pGlyco3 also retains all small glycans (number of monosaccharides ≤ 3) for the peptide search because small glycans have too few Y ions to obtain high glycan scores.

### Peptide search and glycopeptide FDR estimation

In protein sequence processing, every Asn (N) with the sequon ‘N-X-S/T/C (X is not P)’ in all protein sequences is converted to ‘J’ while keeping the same mass and chemical elements as N. J is then the candidate N-glycosylation site, and S/T are the candidate O-glycosylation sites. Proteins are digested into peptide sequences, and peptide modifications are added to the peptide sequences. Modified peptides are also indexed by their masses for O(1) time access. For the given spectrum and each candidate glycan, the peptide mass is deduced by ‘precursor mass − glycan mass’. pGlyco3 then queries the peptide mass from the mass-indexed peptides. For the peptide search, pGlyco3 considers b/y ions and ‘b/y + HexNAc’ in the HCD mode. pGlyco3 further considers c/z ions as well as their hydrogen rearrangement in the merged spectra for the HCD+ETxxD mode. The candidate glycan is also fine-scored by the matched Y ions. The glycan and peptide scoring schemes of pGlyco3 are the same as those of pGlyco 2.0, but some parameters were tuned in pGlyco3 to obtain better identification performance (Supplementary Fig. 6). Only the top-ranked glycopeptide is retained as the final result for each spectrum. For potential chimeric spectra, pGlyco3 removes unreliable mixed glycopeptides by determining whether one’s precursor is another’s isotope. For example, if NeuAc(1) and Fuc(2) are simultaneously identified in the same MS2 scan but with different precursors, the Fuc(2)-glycopeptide will be removed because ‘NeuAc(1) + 1 Da = Fuc(2).’ pGlyco3 also uses the pGlyco 2.0 method to estimate the FDRs for all GPSMs at the glycan, peptide and glycopeptide levels. pGlyco3 skips the glycan FDR estimation step for small glycans.

### Fast glycosylation site localization with pGlycoSite

pGlyco3 determines not only the glycosylation sites but also the composition of the glycan attached to each site. For a given spectrum with the identified peptide and glycan composition, enumerating all possible glycopeptide forms and generating their c/z ions for site localization is not computationally easy. The worst computation complexity could be $O\left( {L \times \mathop {\prod }\limits_{i = 1}^T C_{S + G_i - 1}^{G_i - 1}} \right)$, where *L* is the peptide length, *T* is the number of monosaccharide types, *S* is the number of candidate glycosylation sites, and *G*_*i*_ is the number of the *i*th monosaccharide type (Supplementary Note 3). The enumeration complexity would be exponentially large as *S* and *G*_*i*_ increase, as illustrated in Supplementary Note 3.

In pGlyco3, the pGlycoSite algorithm is designed to avoid enumeration. The key observation for the pGlycoSite algorithm is that, regardless of how many glycopeptide forms there are for a given peptide and glycan composition, the number of all possible c or z ions is. at most. *F* × (*L* − 1). Here, *F* is the number of subglycan compositions for the identified glycan composition, and the subglycan is defined in Supplementary Note 3. pGlyco3 generates a c/z ion table in which each cell contains the glycan-attached c/z ions (Supplementary Note 3). After removing all Y and b/y ions from the given spectrum, the ion table with c/z ions is then matched and scored against the spectrum (called the ScoreTable; Fig. 3b and Supplementary Note 3). pGlycoSite currently uses c/z ion counting scores for each cell of the table, but other comprehensive scoring schemes could be supported in the table if they could achieve better performance.

The best-scored path starting from bottom left [*g*_0_,0] to top right [*G*,*L*] (Fig. 3c and Supplementary Note 3) is then calculated by a dynamic programming algorithm:

$$\begin{array}{l}\mathrm{BestPath}\left[ {g,p} \right] =\\ \left\{ {\begin{array}{*{20}{c}} {\begin{array}{*{20}{c}} {\mathop {{\max }}\limits_{\forall g_s \le g} \mathrm{BestPath}\left[ {g_s,p - 1} \right] + \mathrm{ScoreTable}\left[ {g,p} \right]} & {\mathrm{if}\,\mathrm{IsValidPath}\left( {g_s,g,p} \right)} \end{array}} \\ {\begin{array}{*{20}{c}} \times & {\mathrm{if}\,\mathrm{not}\,\exists g_s \le g\,\mathrm{IsValidPath}\left( {g_s,g,p} \right)} \end{array}} \end{array}} \right.\end{array}$$

where *G* is the identified full glycan composition, *g* refers to a subglycan composition of *G*, *g*_0_ refers to the zero-glycan composition and *p* refers to *p*th position of the peptide sequence. Here, all glycan compositions (from *g*_0_ to *G*) are represented as vectors and hence can be compared with each other. Therefore, *g*_*s*_ ≤ *g* means that *g*_*s*_ is the subglycan of *g*. $\mathrm{IsValidPath}\left( {g_s,g,p} \right)$ is designed to check whether the path starting from $\left[ {g_s,p - 1} \right]$ to [*g*,*p*] is valid (Supplementary Note 3). pGlycoSite sets BestPath[*g*,0]=0 ($\forall g:g_0 \le g \le G$) and iteratively calculates the BestPath table for all *g*_0_ *≤g ≤G* and 0 <*p ≤ L*. BestPath[*G*,*L*] is then the final best path score that will be solved. Finally, pGlycoSite deduces all the paths that can reach the BestPath[*G*,*L*] score by backtracking the BestPath table from [*g*_0_,0] to [*G*,*L*]. If the best-scored path contains the cell [*g*_*s*_,*p* − 1] and [*g*,*p*] with *g*_*s*_ < *g*, then the *p*th amino acid is localized as a site with glycan *g* *− g*_*s*_. pGlycoSite introduces the ‘site-group’ if multiple paths can achieve the same BestPath[*G*,*L*] score (Fig. 3c and Supplementary Note 3). The time complexity of SSGL in pGlycoSite, including the dynamic programming and backtracking for a GPSM, is only *O*(*L* × *F*^2^) (Supplementary Note 3).

### Site localization probability estimation with pGlycoSite

Glycosylation site probability refers to the probability that a site is correctly localized. As the peptide and glycan compositions have been identified for a given MS2 spectrum, an incorrect localization would result from the random assignment of randomly selected subglycans to random sites for the same peptide and glycan compositions. To simulate the incorrect localization for each localized site, pGlycoSite randomly samples 1000 paths from bottom left to top right on the ScoreTable. For a given site or site-group to be estimated, the random paths could overlap with the BestPath, but they must not contain the path that can determine this site or site-group (that is, path from [*g*_*s*_,*p*_*i*_] to [*g*,*p*_*j*_] for site *p*_*i*_(*j* = *i* + 1) or site-group {*p*_*i*_,*p*_*j*–1_} (*j* > *i* + 1)). pGlycoSite then calculates 1,000 ion counting scores of these paths and estimates a Poisson distribution from these random scores. It estimates the *P* values on the basis of the Poisson distribution for the BestPath[*G*,*L*] and the best random score (denoted as RandomBest) and then estimates the probability as follows:

$${\mathrm{Prob}}_{\mathrm{Poisson}} = \frac{{{{{\mathrm{log}}}}\left( {P{\mathrm{value}}\left( {{\mathrm{BestPath}}\left[ {G,L} \right]} \right)} \right)}}{{\log \left( {P{\mathrm{value}}\left( {{\mathrm{BestPath}}\left[ {G,L} \right]} \right)} \right) + {{{\mathrm{log}}}}\left( {P{\mathrm{value}}\left( {\mathrm{RandomBest}} \right)} \right)}}$$

To ensure the localized glycopeptide-spectrum-matching quality, pGlycoSite adds a regularization factor to the estimated Prob_Poisson_, and the final localized probability becomes

$${\mathrm{Prob}} = {\mathrm{Prob}}_{\mathrm{Poisson}} \times r = {\mathrm{Prob}}_{\mathrm{Poisson}} \times \left( {\frac{{\mathrm{BestPath}\left[ {G,L} \right]}}{{2\left( {L - 1} \right)}}} \right)^\alpha ,$$

where *α* is set as a small value (0.05) to ensure that it does not affect the value of Prob_Poisson_. However, when BestPath[*G*,*L*] obtains a very small score, *r* will be close to zero, hence limiting the final Prob value. *L* − 1 is the number of considered c/z ions.

### Validation of the N-glycopeptide search with ^15^N-/^13^C-labeled fission yeast data

The protein sequence database used was the fission yeast protein sequence database (*Schizosaccharomyces pombe*, Swiss-Prot, 2018_08) concatenated with the mouse protein sequence database (*Mus musculus*, Swiss-Prot, 2018_08). Identified GPSMs with mouse peptides would be falsely identified and, hence, mouse peptide GPSMs could be used to test the peptide-level error rates. The N-glycan database for MetaMorpheus, MSFragger and Byonic is the 182-glycan database, which includes 74 NeuAc-containing N-glycan compositions. The N-glycan database for pGlyco3 is the built-in mouse N-glycan database, which contains 1,234 N-glycan compositions (6,662 structures) and has 659 NeuAc-contained compositions. NeuAc-containing N-glycan compositions identified in fission yeast data would be falsely identified and thus could be used to test glycan-level error rates. The detailed search parameters are listed in Supplementary Data. For each software tool, all spectra were regarded as unlabeled spectra while searching, and the identified GPSMs were then validated by using their ^15^N-/^13^C-labeled precursor signals in the MS1 spectra (Fig. 2a). This validation method was also used in our previous works for peptide, glycopeptide and crosslinked peptide identification^8,41,42^. Peptide-level and glycan-level FDRs were also tested by using mouse peptides and NeuAc-containing glycans, respectively (Fig. 2a).

### Validation of the O-glycopeptide search with IHMO data

In inhibitor-initiated homogenous mucin-type O-glycosylation (IHMO), an O-glycan elongation inhibitor, benzyl-N-acetyl-galactosaminide (GalNAc-O-bn), was applied to truncate the O-glycan elongation pathway during cell culture, generating cells with only truncated HexNAc(1) or HexNAc(1)NeuAc(1) O-glycans. sceHCD-pd-EThcD spectra were generated after O-glycopeptides were enriched by FASP^43^, and experimental details are shown in Supplementary Note 4. IHMO in HEK-293 cells was then verified by laser confocal microscopy, as displayed in Supplementary Fig. 4. Spectra were then searched by pGlyco3, MetaMorpheus, MSFragger and Byonic, and the search parameters are listed in Supplementary Data. For all software tools, Hex-containing O-glycopeptides could be still identified due to the inhibitor’s imperfect efficiencies. The Hex-contained O-GPSMs were further validated by the summed intensities of the Hex-diagnostic ions (163.060 and 366.139 m/z) in their HCD spectra. The summed intensity threshold was set as 10% of the base peak.

### Validation of the pGlycoSite algorithm

For the given SSGL probabilities (Prob) of all identified GPSMs, the SSGL-FDR could be estimated as follows:

$$\widehat {\mathrm{FDR}}_{SL}\left( x \right) = \frac{{\mathop {\sum }\nolimits_{\forall i:1 \le i \le N,\,\mathrm{Prob}_i \ge x} \left( {1 - \mathrm{Prob}_i} \right)}}{{\mathop {\sum }\nolimits_{i = 1}^N I\left( {\mathrm{Prob}_i \ge x} \right)}},$$

where $\widehat {\mathrm{FDR}}_{SL}(x)$ is the estimated SSGL-FDR for a given probability threshold *x*, *N* is the total number of localized sites and *I*(bool) is the indicator function that returns 1 when bool is true and 0 otherwise. It is not easy to validate the estimated SSGL probability for a given site, but we can validate the accuracy of $\widehat {\mathrm{FDR}_{SL}}\left( x \right)$, enabling SSGL probability validation from another perspective. In this work, we designed four methods to validate $\widehat {\mathrm{FDR}}_{SL}(x)$: synthetic double-site N-glycopeptide validation, multienzyme-based validation, entrapment-based validation and OpeRATOR-based validation.

A double-site N-glycopeptide ‘NVN[H(5)N(4)]ISYTVN[H(5)N(4)]DSFFPQRPQK’ was synthesized and its 3+ and 4+ HCD+ETxxD spectra were continuously acquired by using PRM. To enable SSGL validation, we searched the spectra against the human glycan database and a protein database with only the synthetic peptide sequence. Thus, we could always identify the same peptide sequence with different localized glycans. SSGL would be true if the localized glycan was H(5)N(4); otherwise, it was false. The real SSGL-FDR could then be calculated as ${\mathrm{FDR}}_{syn} = \frac{\# {\mathrm{False}}}{\# {\mathrm{False}} \space + \space \# {\mathrm{True}}}$.

To validate SSGL for double-site N-glycopeptides under more comprehensive situations, we used pGlyco3 to identify and localize the double-site N-glycopeptides with the peptide sequence ‘K.THTN(272)ISESHPN(279)ATF.S’ of IGHM digested with chymotrypsin and trypsin. For all identified site-specific N-glycans, the theoretical masses of further ‘trypsin+Glu-C’-digested glycopeptides were calculated. Then, we used PRM to trigger the HCD spectra of ‘trypsin+Glu-C’-digested glycopeptides and identified these single-site spectra to verify the double-site N-glycopeptides. The SSGL would be true if the localized N-glycan on ‘K.THTN(272)ISESHPN(279)ATF.S’ could be identified by the single-site spectra; otherwise, it was false. The Glu-C-suggested SSGL-FDR could then be calculated as ${\mathrm{FDR}}_{\mathrm{GluC}} = \frac{\# {\mathrm{False}}}{\# {\mathrm{False}} \space + \space \# {\mathrm{True}}}$.

For entrapment-based validation, after N-glycopeptide data were searched, the sites of GPSMs were localized using pGlycoSite by regarding the candidate sites as ‘J/S/T’, which could be enabled by setting ‘glycosylation_sites=JST’ in the search parameter file. For a given GPSM, it would be a true positive (TP_trap_) if J were the only localized sites; otherwise, all sites were false positives (FP_trap_). The entrapment-based SSGL-FDR could be calculated as

$${\mathrm{FDR}}_{\mathrm{trap}}\left( x \right) = \frac{{\# {\mathrm{FP}}_{\mathrm{trap}}\left( {\mathrm{Prob}}\space \ge\space x \right)}}{{\# {\mathrm{FP}}_{\mathrm{trap}}\left( {{\mathrm{Prob}}\space \ge\space x} \right)\space +\space {\# {\mathrm{TP}}_{\mathrm{trap}}}\left( {{\mathrm{Prob}}\space \ge \space x} \right)}}$$

for a given probability threshold *x*. Then, the SSGL probabilities of pGlycoSite could be validated by comparing $\widehat {\mathrm{FDR}}_{\mathrm{SL}}\left( x \right)$ with FDR_trap_(*x*).

For OpeRATOR-based validation, we used the data digested by OpeRATOR^37^. Only the GPSMs with their peptides starting with ST at the N-terminal were used for the validation. Then, for a given GPSM, we regarded it as the true positive (TP_OpR_) if localized sites contained a site that was at the N-terminal S/T otherwise, it was regarded as a false positive (FP_OpR_). The OpeRATOR-based SSGL-FDR (FDR_OpR_(*x*)) could be calculated from TP_OpR_(*x*) and FP_OpR_(*x*), which is similar to FDR_trap_(*x*).

Comparisons of the $\widehat {\mathrm{FDR}}_{\mathrm{SL}}\left( x \right)$ of pGlycoSite with FDR_trap_(*x*) and FP_OpR_(*x*) are displayed in Fig. 3d and Supplementary Fig. 2. We also compared $\widehat {\mathrm{FDR}}_{\mathrm{SL}}\left( x \right)$ of MetaMorpheus with FDR_OpR_(*x*), as shown in Supplementary Fig. 2.

### Analysis of aH-glycopeptides in yeast samples

‘aH’ is defined as a Hex with an ammonia adduct. Peptides were searched by the yeast protein sequence databases (*S. pombe* for fission yeast and *Saccharomyces cerevisiae* for budding yeast, Swiss-Prot, 2018_08). N-glycan parts were searched against the high-mannose-only N-glycan database, and O-Man glycan parts were searched against the Hex-only glycan database. aH was regarded as a modified Hex for the pGlyco3 search, and the maximal number of aHs was set as two per glycan. For the O-Man-glycopeptide search, the glycopeptide-diagnostic ion was set as Hex (163.060 m/z). The ^15^N-/^13^C-labeled fission yeast (PXD005565) results were also validated by the ^15^N-labeled and ^13^C-labeled precursor signals in the MS1 spectra. For the ^15^N/^13^C validation of aH identifications, as the ammonia adduct may be introduced during sample processing or MS steps, it could not be labeled by ^15^N; hence, we computationally designed a new element called ‘14N’, which would not be converted into ^15^N for MS1 signal extraction. The element composition is recorded in the ‘element.ini’ file in the software package, demonstrating the flexibility of pGlyco3 for the analysis of new monosaccharides or modified saccharide units. We also verified the aH-N-glycans by analyzing the MS data of released N-glycans in fission yeast samples (Supplementary Note 4).

O-Man glycopeptides of fission yeast were also analyzed by HCD followed by EThcD to investigate the O-mannosylation sites. The data were searched by the ‘HCD+EThcD’ mode of pGlyco3, and the sites were localized by pGlycoSite. See Supplementary Note 4 for details.

### Reporting Summary

Further information on research design is available in the Nature Research Reporting Summary linked to this article.

## Online content

Any methods, additional references, Nature Research reporting summaries, source data, extended data, supplementary information, acknowledgements, peer review information; details of author contributions and competing interests; and statements of data and code availability are available at 10.1038/s41592-021-01306-0.

## Supplementary information

Supplementary Information Supplementary Tables 1–3, Figs. 1–11 and Notes 1–5.

Reporting Summary

Supplementary Data All additional data files.

## Data Availability

Data generated in this work, including yeast glycoproteomic data, yeast N-glycomic data, IHMO O-glycoproteomic data and human serum O-glycoproteomic data, can be downloaded from MassIVE (https://massive.ucsd.edu/) with identifier MSV000086771. sceHCD RAW files of mixed unlabeled, ^15^N-labeled, and ^13^C-labeled fission yeast glycopeptide samples were downloaded from PXD005565 on PRIDE^8^. 30×6 h sceHCD RAW files of five mouse tissues were downloaded from PXD005411, PXD005412, PXD005413, PXD005553, and PXD005555 on PRIDE^8^. sceHCD-pd-EThcD RAW files of human milk and Chinese hamster ovary cell samples were obtained from MassIVE (dataset MSV000083710)^7^. RAW files of OpeRATOR-processed O-glycopeptide data were obtained from PXD020077 on PRIDE^10^. Detailed search parameters for all these RAW data files are listed in Supplementary Data. All the pGlyco3 result files can also be found in Supplementary Data.

## Code availability

pGlyco3 can be downloaded from https://github.com/pFindStudio/pGlyco3/releases. The pGlyco3 version used in this manuscript can be downloaded from Zenodo (10.5281/zenodo.5517102). Analysis results and Python Notebooks to reproduce the comparison results can be downloaded from https://figshare.com/projects/Searched_results_and_python_notebooks_for_pGlyco3_manuscript/97592. The versions of other search engines are displayed in the Reporting Summary.
