# Supplementary material for: Precise, fast and comprehensive analysis of intact glycopeptides and modified glycans with pGlyco3
Source: Nat Methods. 2021 Nov 25;18(12):1515–23. doi: 10.1038/s41592-021-01306-0 (PMC8648562; doi:10.1038/s41592-021-01306-0)
Supplement: Supplementary file 2 — Reporting Summary [file 41592_2021_1306_MOESM2_ESM.pdf]

nature research

Corresponding author(s): Wen-Feng Zeng
Last updated by author(s): Aug 30, 2021

Reporting Summary

Nature Research wishes to improve the reproducibility of the work that we publish. This form provides structure for consistency and transparency in reporting. For further information on Nature Research policies, see our Editorial Policies and the Editorial Policy Checklist.

Statistics

For all statistical analyses, confirm that the following items are present in the figure legend, table legend, main text, or Methods section.

n/a	Confirmed	
☒	☐	The exact sample size (*n*) for each experimental group/condition, given as a discrete number and unit of measurement
☐	☒	A statement on whether measurements were taken from distinct samples or whether the same sample was measured repeatedly
☒	☐	The statistical test(s) used AND whether they are one- or two-sided<br>*Only common tests should be described solely by name; describe more complex techniques in the Methods section.*
☒	☐	A description of all covariates tested
☒	☐	A description of any assumptions or corrections, such as tests of normality and adjustment for multiple comparisons
☒	☐	A full description of the statistical parameters including central tendency (e.g. means) or other basic estimates (e.g. regression coefficient) AND variation (e.g. standard deviation) or associated estimates of uncertainty (e.g. confidence intervals)
☒	☐	For null hypothesis testing, the test statistic (e.g. *F*, *t*, *r*) with confidence intervals, effect sizes, degrees of freedom and *P* value noted<br>*Give P values as exact values whenever suitable.*
☒	☐	For Bayesian analysis, information on the choice of priors and Markov chain Monte Carlo settings
☒	☐	For hierarchical and complex designs, identification of the appropriate level for tests and full reporting of outcomes
☐	☒	Estimates of effect sizes (e.g. Cohen's *d*, Pearson's *r*), indicating how they were calculated

Our web collection on statistics for biologists contains articles on many of the points above.

Software and code

Policy information about availability of computer code

Data collection	No software was used in the data collection.
Data analysis	pGlyco3 is freely available on https://github.com/pFindStudio/pGlyco3. The software package could be downloaded via https://github.com/pFindStudio/pGlyco3/releases, and the license is available upon request. The pGlyco3 version used in this manuscript was pGlyco3.0_build20210615.<br><br>Analysis results and Python Notebooks to reproduce the comparison results could be downloaded from https://figshare.com/projects/Searched_results_and_python_notebooks_for_pGlyco3_manuscript/97592.<br><br>Other software tools for comparison: MetaMorpheus (v0.0.312, downloaded in 2020.10), MSFragger (v3.1.1 with FragPipe v14.0 and philosopher v3.3.11, downloaded in 2020.10), and Byonic (v3.10). GlycoWorkbench (v2.1 build 146) and Python (v3.8) were used in this work.

For manuscripts utilizing custom algorithms or software that are central to the research but not yet described in published literature, software must be made available to editors and reviewers. We strongly encourage code deposition in a community repository (e.g. GitHub). See the Nature Research guidelines for submitting code & software for further information.

Data

Policy information about availability of data

All manuscripts must include a data availability statement. This statement should provide the following information, where applicable:

- Accession codes, unique identifiers, or web links for publicly available datasets
- A list of figures that have associated raw data
- A description of any restrictions on data availability

Data generated in this work, including yeast glycoproteomic data, yeast N-glycomic data, IHMO O-glycoproteomic data, and human serum O-glycoproteomic data, could be downloaded from MassIVE (https://massive.ucsd.edu/) with identifier MSV000086771. sceHCD RAW files of mixed unlabeled, 15N-labeled, and 13C-labeled fission yeast glycopeptide samples were downloaded from PXD0055658 on PRIDE. 30×6 h sceHCD RAW files of five mouse tissues were downloaded from PXD005411, PXD005412, PXD005413, PXD005553, and PXD0055558 on PRIDE. sceHCD-pd-EThcD RAW files of human milk and Chinese hamster ovary cell (CHO) samples were obtained from MassIVE (dataset MSV0000837107). RAW files of OpeRATOR-processed O-glycopeptide data were obtained from PXD02007710 on PRIDE. Detailed search parameters for all these RAW data files are listed in Supplementary Data. All the pGlyco3 result files can also be found in Supplementary Data.

Field-specific reporting

Please select the one below that is the best fit for your research. If you are not sure, read the appropriate sections before making your selection.

☒ Life sciences ☐ Behavioural & social sciences ☐ Ecological, evolutionary & environmental sciences

For a reference copy of the document with all sections, see nature.com/documents/nr-reporting-summary-flat.pdf

Life sciences study design

All studies must disclose on these points even when the disclosure is negative.

Sample size	The mixtures from ten healthy human serum samples were used as complex samples to demonstrate the ability of the software pGlyco3 for site-specific O-glycosylation identification. The collected samples were mainly used to generate MS data for demonstration of the software performance. Since no biological conclusion were draw, the sample size is sufficient,
Data exclusions	No data were excluded from the analyses.
Replication	Triplicates of LC-MS/MS were performed in yeast glycoproteome, IHMO and human serum O-glycoproteome. All attempts at replication were successful.
Randomization	The serum mixtures, which were used to generate MS data for demonstration of the software performance, were collected from ten healthy people, including five women and five men aged from 25 to 45. No biological conclusions were draw. The results presented was to demonstrate the performance of the software for site-specific O-glycosylation identification.
Blinding	Not applicable. The results presented were the output of the software. No blinding is required.

Reporting for specific materials, systems and methods

We require information from authors about some types of materials, experimental systems and methods used in many studies. Here, indicate whether each material, system or method listed is relevant to your study. If you are not sure if a list item applies to your research, read the appropriate section before selecting a response.

Materials & experimental systems

n/a	Involved in the study	
☐	☒	Antibodies
☐	☒	Eukaryotic cell lines
☒	☐	Palaeontology and archaeology
☒	☐	Animals and other organisms
☐	☒	Human research participants
☒	☐	Clinical data
☒	☐	Dual use research of concern

Methods

n/a	Involved in the study	
☒	☐	ChIP-seq
☒	☐	Flow cytometry
☒	☐	MRI-based neuroimaging

Antibodies

Antibodies used	The following product was used in this experiment: Sialosyl-Tn Antigen Monoclonal Antibody (STn 219) from Thermo Fisher Scientific, catalog # MA1-90577, RRID AB_2264594； Tn Antigen Monoclonal Antibody (Tn 218) from Thermo Fisher Scientific, catalog #

	MA1-90544, RRID AB_1961080; CD176 Monoclonal Antibody (A78-G/A7) from Thermo Fisher Scientific, catalog # MA5-13466, RRID AB_10986703.
Validation	MA1-90577 targets Sialosyl-Tn Antigen in immunocytochemistry, immunofluorescence, immunohistochemistry and western blot and shows reactivity with Human species. MA1-90544 targets Tn Antigen in immunocytochemistry, immunofluorescence and shows reactivity with Human species, immunohistochemistry and western blot. MA5-13466 targets Thomsen-Friedenreich Antigen in immunofluorescence and immohistochemistry (Paraffin) applications and shows reactivity with Human and Rat samples.

Eukaryotic cell lines

Policy information about cell lines

Cell line source(s)	HEK-293, Jurkat Clone E6-1, MCF-7, Hela, and Hep 3B were purchased from National Collection of Authenticated Cell Cultures of China
Authentication	All cell lines were detected by STR
Mycoplasma contamination	Cell lines were tested negative for mycoplasma contamination
Commonly misidentified lines (See ICLAC register)	No commonly misidentified cell lines were used in the study.

Human research participants

Policy information about studies involving human research participants

Population characteristics	The mixtures from ten healthy human serum samples were used as complex samples only to demonstrate the ability of the software pGlyco3 for site-specific O-glycosylation identification. The ten samples were collected from ten physically healthy volunteers. The ten volunteers were five women and five men, aged from 25 to 45.
Recruitment	Ten healthy participants, from whom serum samples were collected, were volunteers recruited by Zhongshan hospital, Fudan university. The collected samples were mainly used to generate MS data for demonstration of the software performance. No biological conclusion were draw, and there were no potential self-selection biases or other biases to impact the results.
Ethics oversight	Informed consent was obtained under protocols that were approved by an institutional review board. The research followed the tenet of the Declaration of Helsinki and was approved by the Ethics Committee of the Fudan University.

Note that full information on the approval of the study protocol must also be provided in the manuscript.
