# Supplementary material for: Precise, fast and comprehensive analysis of intact glycopeptides and modified glycans with pGlyco3
Source: Nat Methods. 2021 Nov 25;18(12):1515–23. doi: 10.1038/s41592-021-01306-0 (PMC8648562; doi:10.1038/s41592-021-01306-0)
Supplement: Supplementary file 3 — All additional data files. [file 41592_2021_1306_MOESM3_ESM.zip › Supplementary Data/pGlycoSite ScoreTable Examples.pdf]

ScoreTable of Fig. 1d-f

H N (H=Hex, N=HexNAc)

2 2			z	cz	cz	cz	cz	cz	cz	cz	z		
2 1			z	c	c								
2 0													
1 2	z			c				z					
1 1	cz		c							z			
1 0													
0 2													
0 1									z				
0 0				z					z				

T P S P T V A H E S N W A K

2 2	0	0	2	3	5	7	9	11	13	15	17	18	18
2 1	0	0	0	0	1	2	2	2	2	×	×	×	×
2 0	×	×	×	×	×	×	×	×	×	×	×	×	×
1 2	×	×	×	×	×	×	×	×	×	×	×	×	×
1 1	0	2	2	3	3	3	3	3	3	×	×	×	×
1 0	×	×	×	×	×	×	×	×	×	×	×	×	×
0 2	×	×	×	×	×	×	×	×	×	×	×	×	×
0 1	0	0	0	0	0	1	1	1	1	×	×	×	×
0 0	0	0	0	0	1	0	0	0	0	×	×	×	×

T P S P T V A H E S N W A K

× The glycan g or $G - g$ is not a legal glycan. In this case, g is N(2) and G is H(2)N(2). $G - g$=H(2) is not a legal O-glycan (not in the glycan DB), thus this cell is invalid

× There are no candidate sites in the prefix sequence, thus no glycans can be localized

× There are no candidate sites in the suffix sequence, all glycans must be localized before

Invalid cell which contains random/false matches. It will be removed during the dynamic programming in pGlycoSite

G is the full glycan composition. Here G is H(2)N(2)

g is the glycan composition of the given row

ScoreTable of Fig. 3f

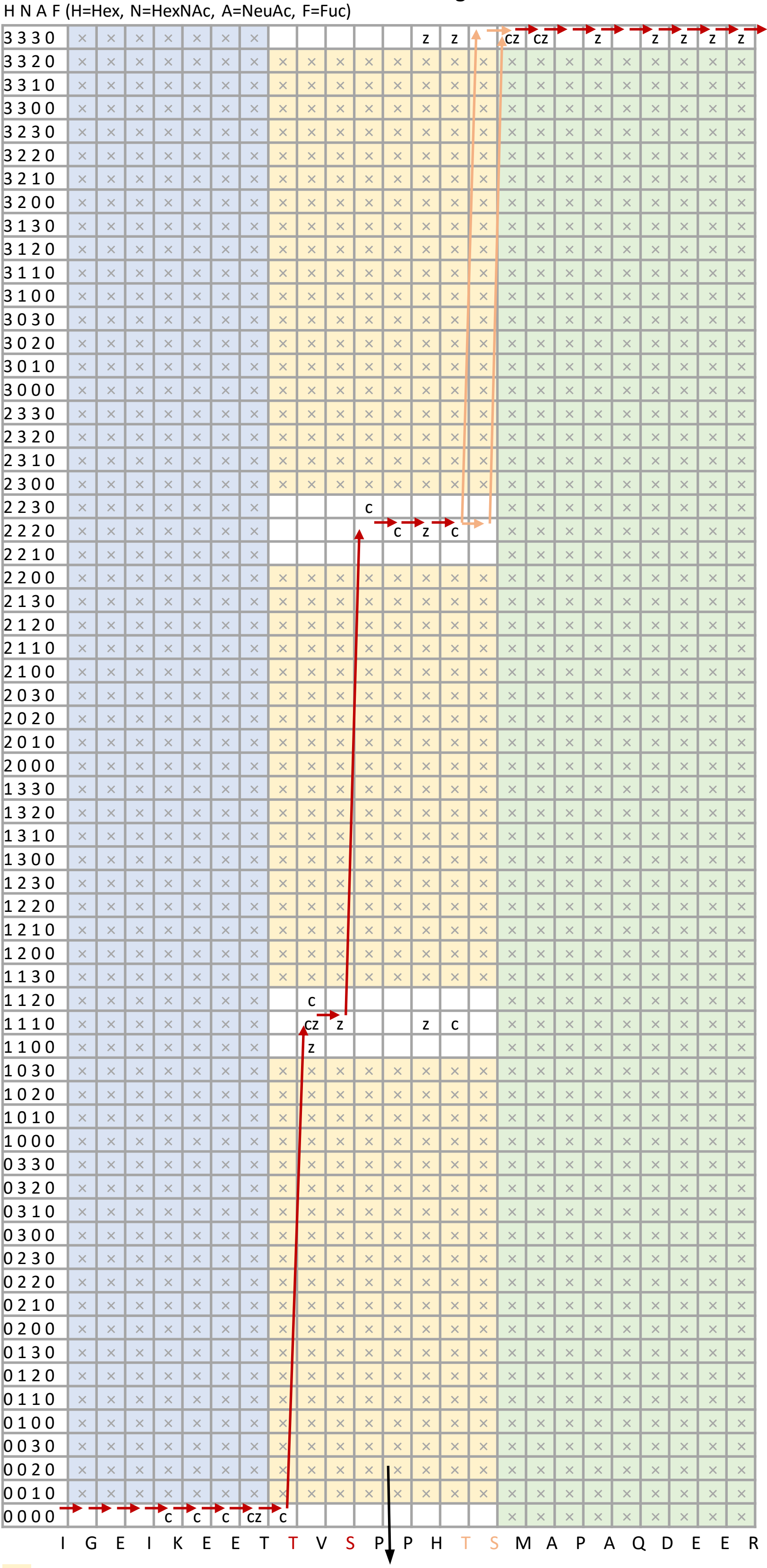

× The glycan g or $G - g$ is not a legal glycan. In this case, g is A(2) and G is H(3)N(3)A(3). g is not a legal O-glycan (not in the glycan DB), thus this cell is invalid

× There are no candidate sites in the prefix sequence, thus no glycans can be localized

× There are no candidate sites in the suffix sequence, all glycans must be localized before
