# Supplementary material for: Precise, fast and comprehensive analysis of intact glycopeptides and modified glycans with pGlyco3
Source: Nat Methods. 2021 Nov 25;18(12):1515–23. doi: 10.1038/s41592-021-01306-0 (PMC8648562; doi:10.1038/s41592-021-01306-0)

## Slide 1
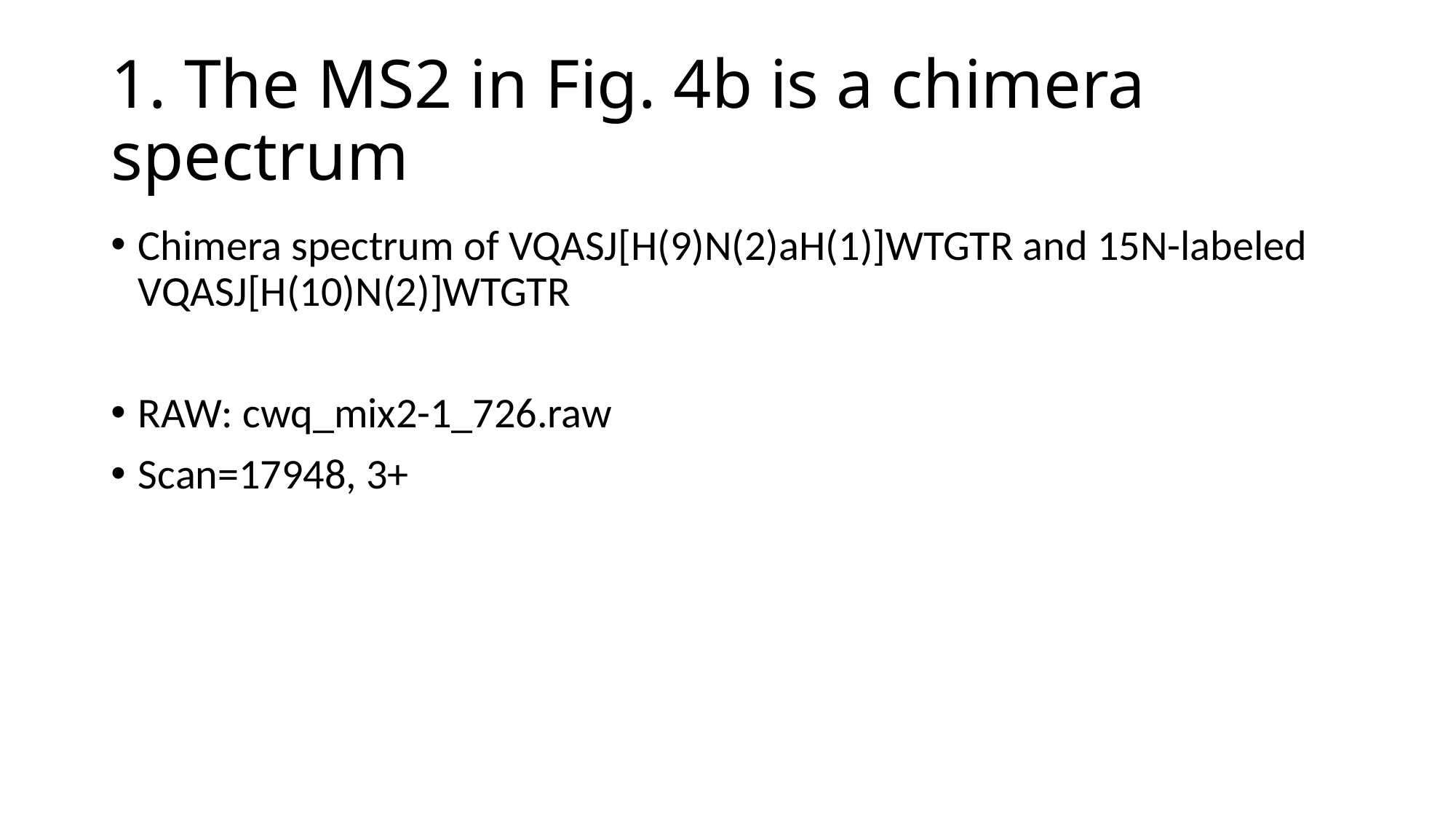

# 1. The MS2 in Fig. 4b is a chimera spectrum
Chimera spectrum of VQASJ[H(9)N(2)aH(1)]WTGTR and 15N-labeled VQASJ[H(10)N(2)]WTGTR
RAW: cwq_mix2-1_726.raw
Scan=17948, 3+

## Slide 2
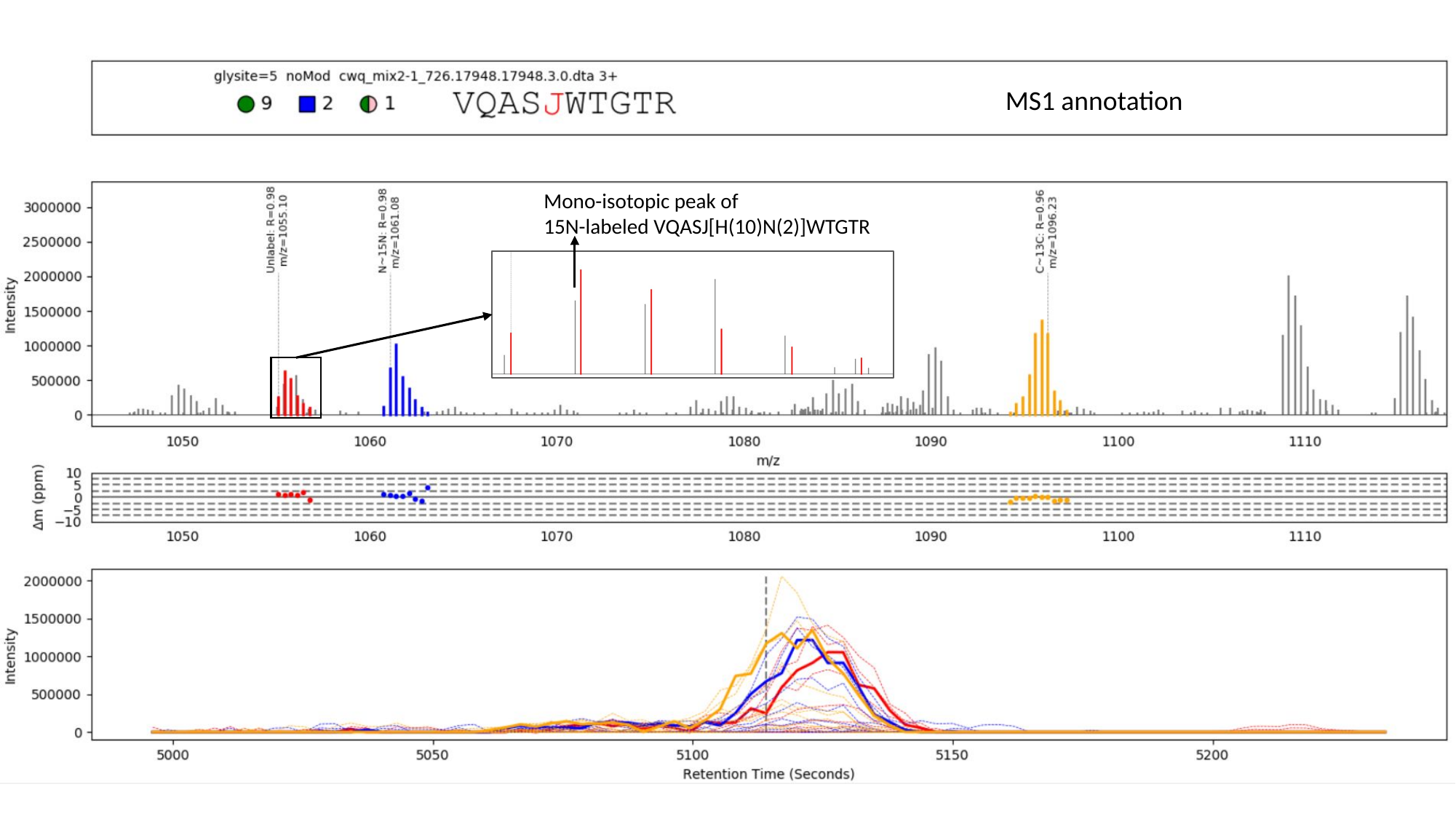

MS1 annotation
Mono-isotopic peak of
15N-labeled VQASJ[H(10)N(2)]WTGTR

## Slide 3
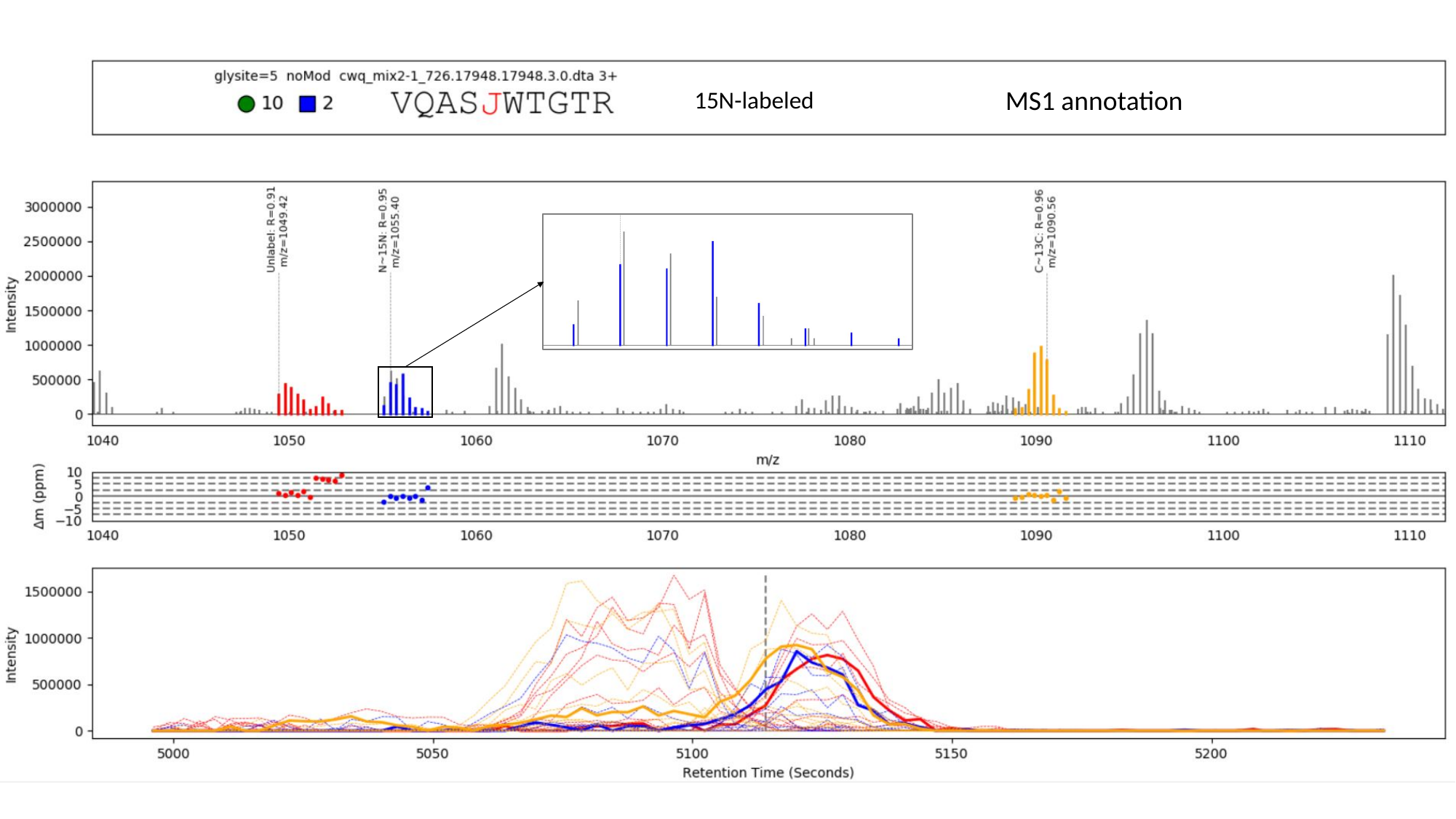

MS1 annotation
15N-labeled

## Slide 4
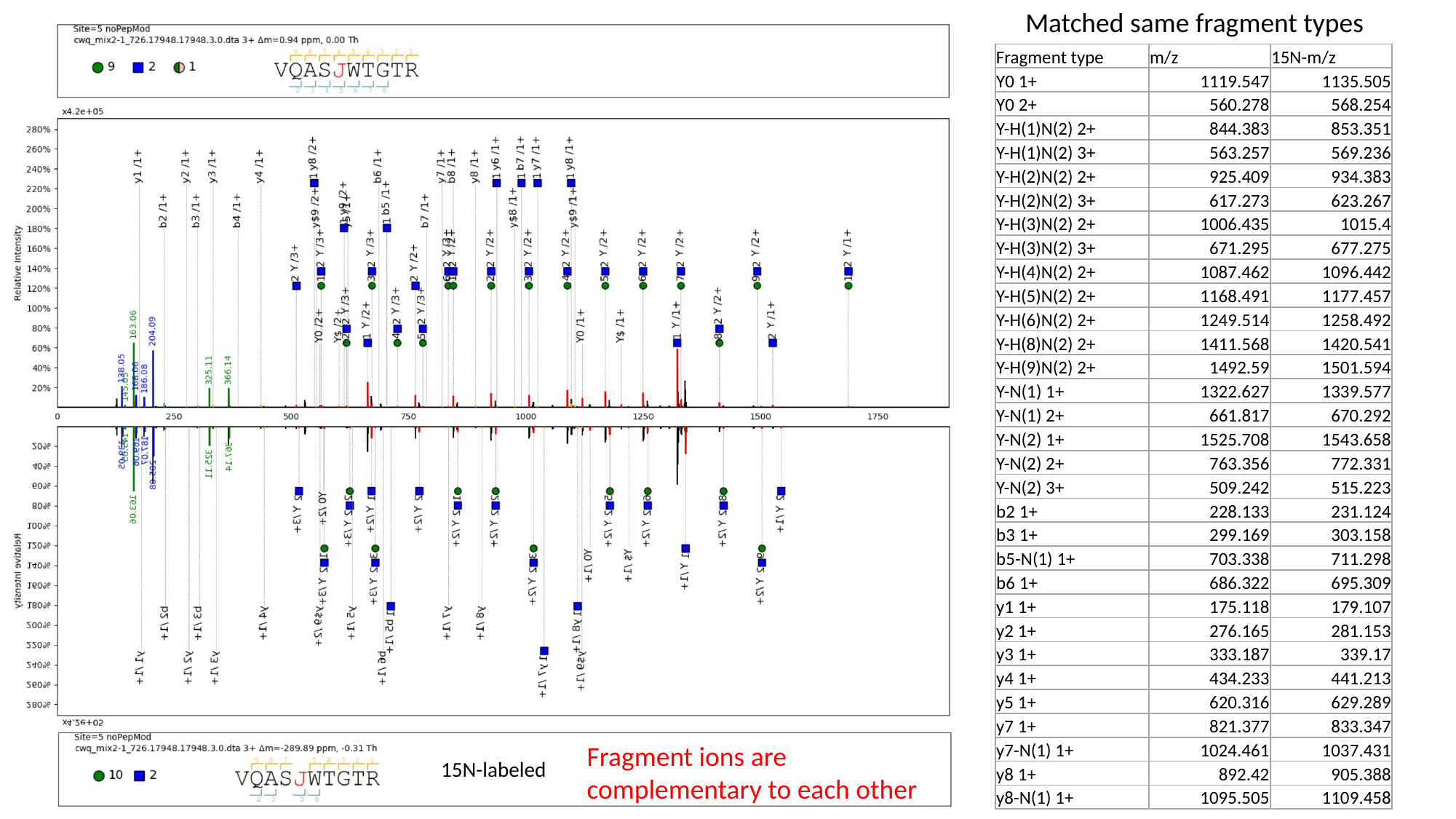

Matched same fragment types
| Fragment type | m/z | 15N-m/z |
| --- | --- | --- |
| Y0 1+ | 1119.547 | 1135.505 |
| Y0 2+ | 560.278 | 568.254 |
| Y-H(1)N(2) 2+ | 844.383 | 853.351 |
| Y-H(1)N(2) 3+ | 563.257 | 569.236 |
| Y-H(2)N(2) 2+ | 925.409 | 934.383 |
| Y-H(2)N(2) 3+ | 617.273 | 623.267 |
| Y-H(3)N(2) 2+ | 1006.435 | 1015.4 |
| Y-H(3)N(2) 3+ | 671.295 | 677.275 |
| Y-H(4)N(2) 2+ | 1087.462 | 1096.442 |
| Y-H(5)N(2) 2+ | 1168.491 | 1177.457 |
| Y-H(6)N(2) 2+ | 1249.514 | 1258.492 |
| Y-H(8)N(2) 2+ | 1411.568 | 1420.541 |
| Y-H(9)N(2) 2+ | 1492.59 | 1501.594 |
| Y-N(1) 1+ | 1322.627 | 1339.577 |
| Y-N(1) 2+ | 661.817 | 670.292 |
| Y-N(2) 1+ | 1525.708 | 1543.658 |
| Y-N(2) 2+ | 763.356 | 772.331 |
| Y-N(2) 3+ | 509.242 | 515.223 |
| b2 1+ | 228.133 | 231.124 |
| b3 1+ | 299.169 | 303.158 |
| b5-N(1) 1+ | 703.338 | 711.298 |
| b6 1+ | 686.322 | 695.309 |
| y1 1+ | 175.118 | 179.107 |
| y2 1+ | 276.165 | 281.153 |
| y3 1+ | 333.187 | 339.17 |
| y4 1+ | 434.233 | 441.213 |
| y5 1+ | 620.316 | 629.289 |
| y7 1+ | 821.377 | 833.347 |
| y7-N(1) 1+ | 1024.461 | 1037.431 |
| y8 1+ | 892.42 | 905.388 |
| y8-N(1) 1+ | 1095.505 | 1109.458 |
Fragment ions are complementary to each other
15N-labeled

## Slide 5
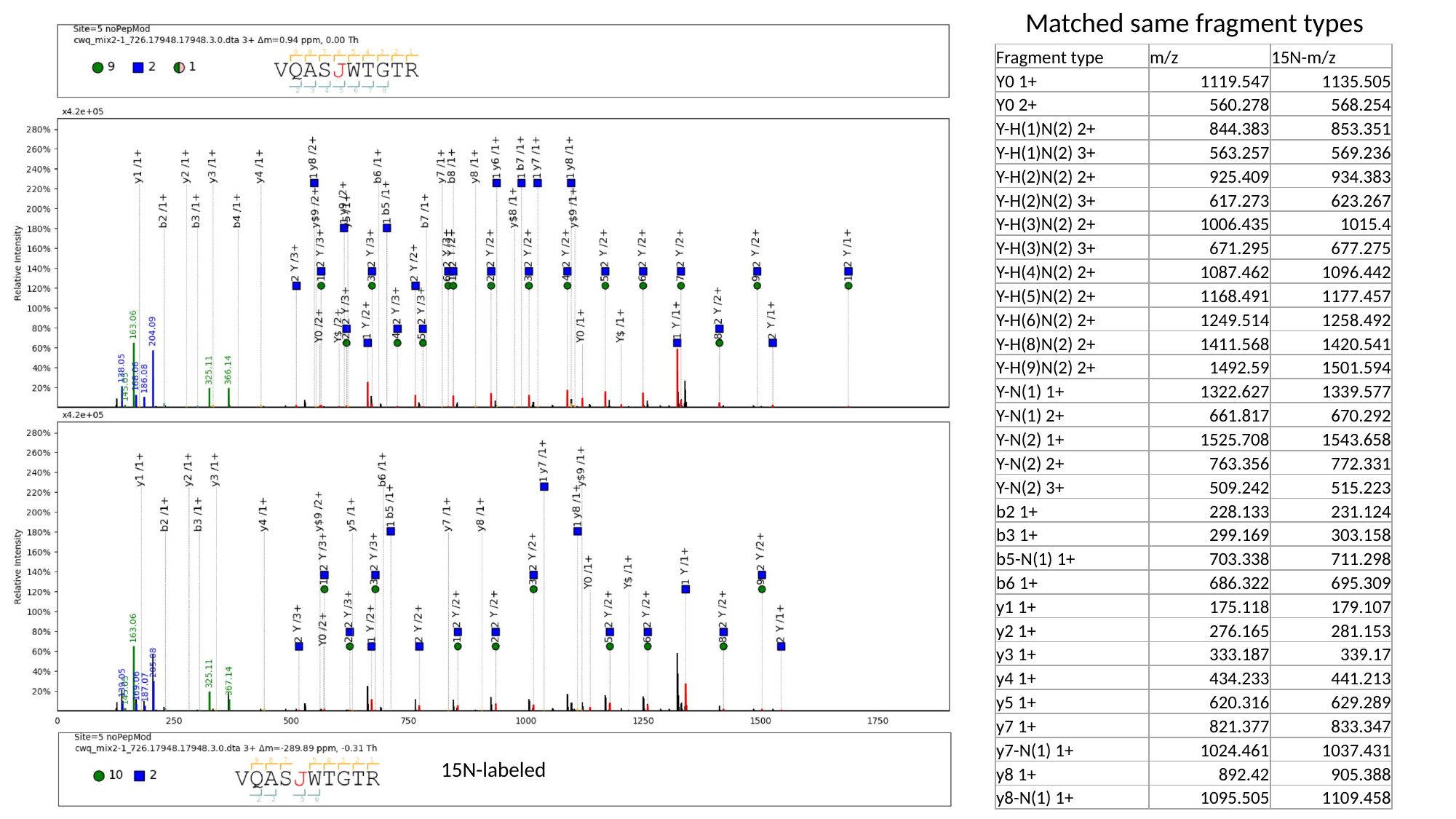

Matched same fragment types
| Fragment type | m/z | 15N-m/z |
| --- | --- | --- |
| Y0 1+ | 1119.547 | 1135.505 |
| Y0 2+ | 560.278 | 568.254 |
| Y-H(1)N(2) 2+ | 844.383 | 853.351 |
| Y-H(1)N(2) 3+ | 563.257 | 569.236 |
| Y-H(2)N(2) 2+ | 925.409 | 934.383 |
| Y-H(2)N(2) 3+ | 617.273 | 623.267 |
| Y-H(3)N(2) 2+ | 1006.435 | 1015.4 |
| Y-H(3)N(2) 3+ | 671.295 | 677.275 |
| Y-H(4)N(2) 2+ | 1087.462 | 1096.442 |
| Y-H(5)N(2) 2+ | 1168.491 | 1177.457 |
| Y-H(6)N(2) 2+ | 1249.514 | 1258.492 |
| Y-H(8)N(2) 2+ | 1411.568 | 1420.541 |
| Y-H(9)N(2) 2+ | 1492.59 | 1501.594 |
| Y-N(1) 1+ | 1322.627 | 1339.577 |
| Y-N(1) 2+ | 661.817 | 670.292 |
| Y-N(2) 1+ | 1525.708 | 1543.658 |
| Y-N(2) 2+ | 763.356 | 772.331 |
| Y-N(2) 3+ | 509.242 | 515.223 |
| b2 1+ | 228.133 | 231.124 |
| b3 1+ | 299.169 | 303.158 |
| b5-N(1) 1+ | 703.338 | 711.298 |
| b6 1+ | 686.322 | 695.309 |
| y1 1+ | 175.118 | 179.107 |
| y2 1+ | 276.165 | 281.153 |
| y3 1+ | 333.187 | 339.17 |
| y4 1+ | 434.233 | 441.213 |
| y5 1+ | 620.316 | 629.289 |
| y7 1+ | 821.377 | 833.347 |
| y7-N(1) 1+ | 1024.461 | 1037.431 |
| y8 1+ | 892.42 | 905.388 |
| y8-N(1) 1+ | 1095.505 | 1109.458 |
15N-labeled

## Slide 6
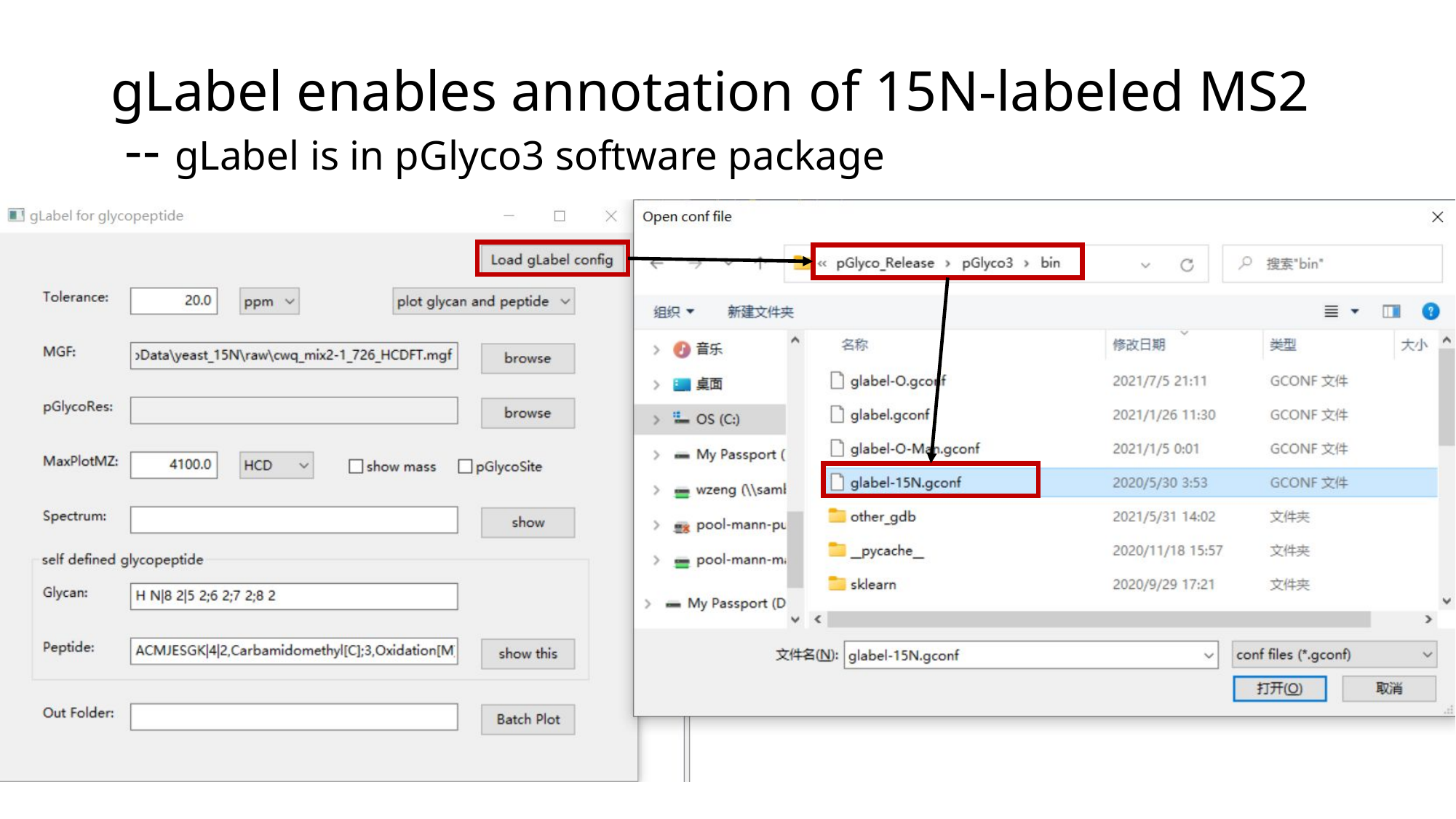

# gLabel enables annotation of 15N-labeled MS2 -- gLabel is in pGlyco3 software package

## Slide 7
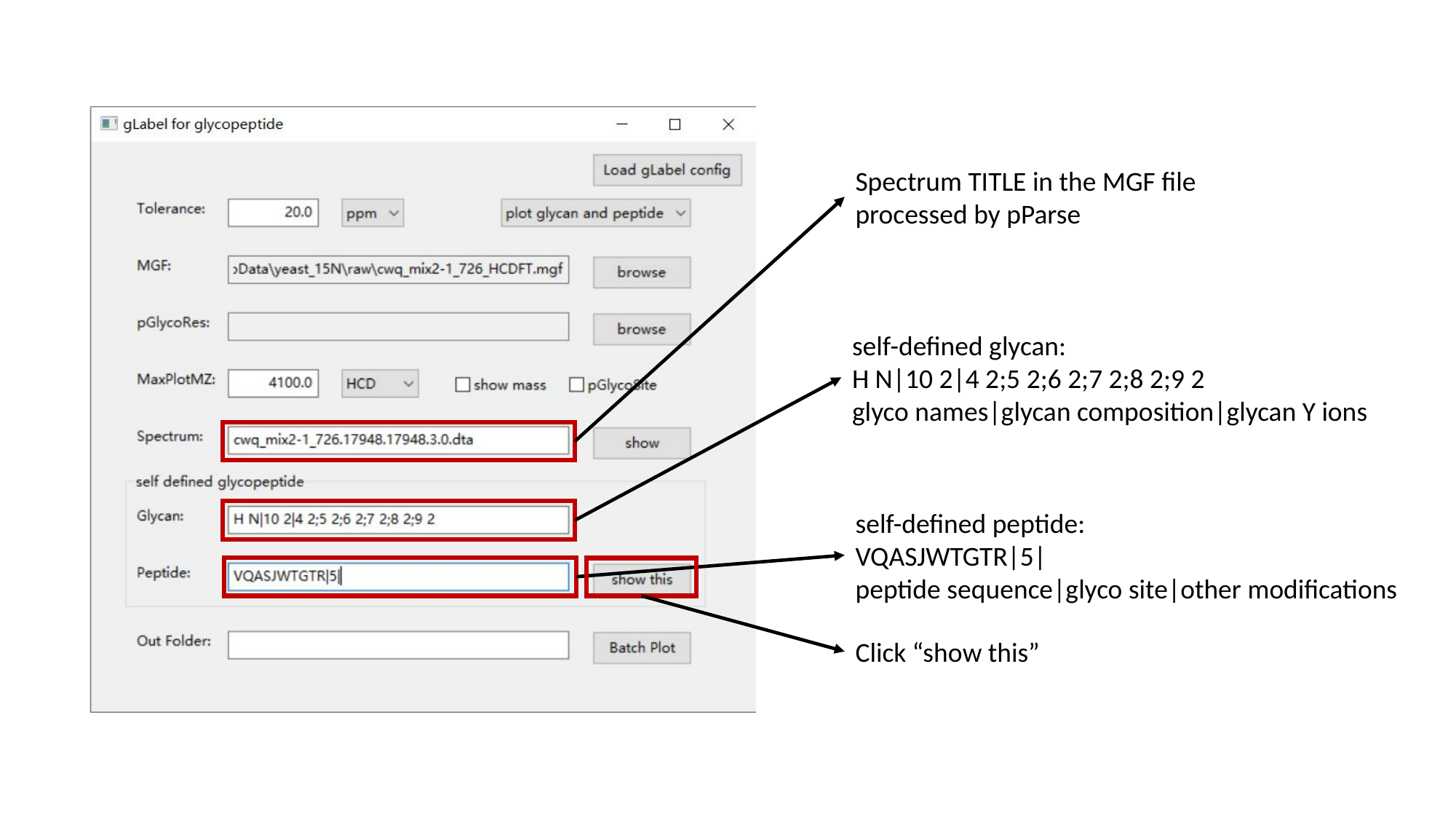

Spectrum TITLE in the MGF file processed by pParse
self-defined glycan:
H N|10 2|4 2;5 2;6 2;7 2;8 2;9 2
glyco names|glycan composition|glycan Y ions
self-defined peptide:
VQASJWTGTR|5|
peptide sequence|glyco site|other modifications
Click “show this”

## Slide 8
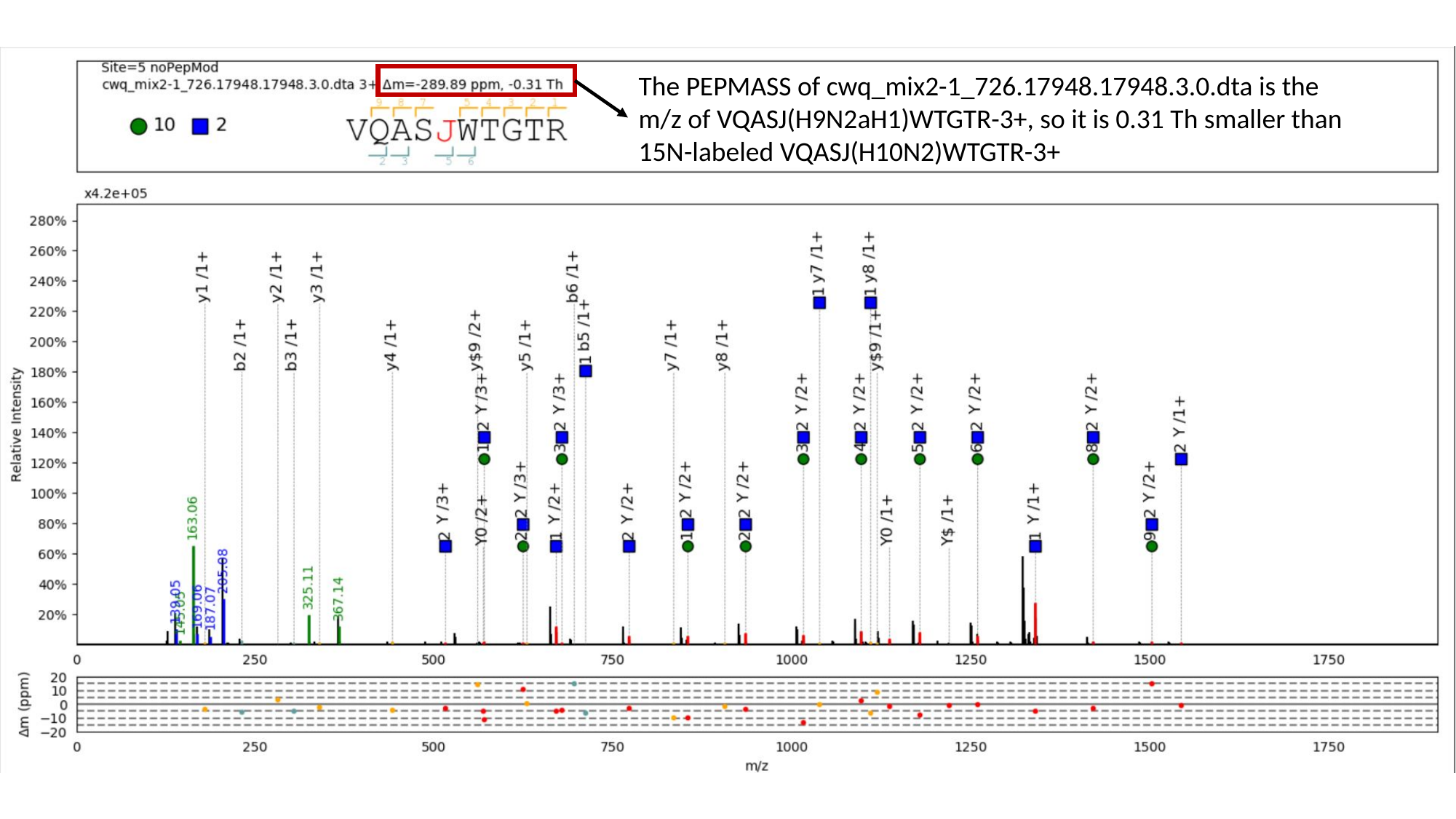

The PEPMASS of cwq_mix2-1_726.17948.17948.3.0.dta is the m/z of VQASJ(H9N2aH1)WTGTR-3+, so it is 0.31 Th smaller than 15N-labeled VQASJ(H10N2)WTGTR-3+

## Slide 9
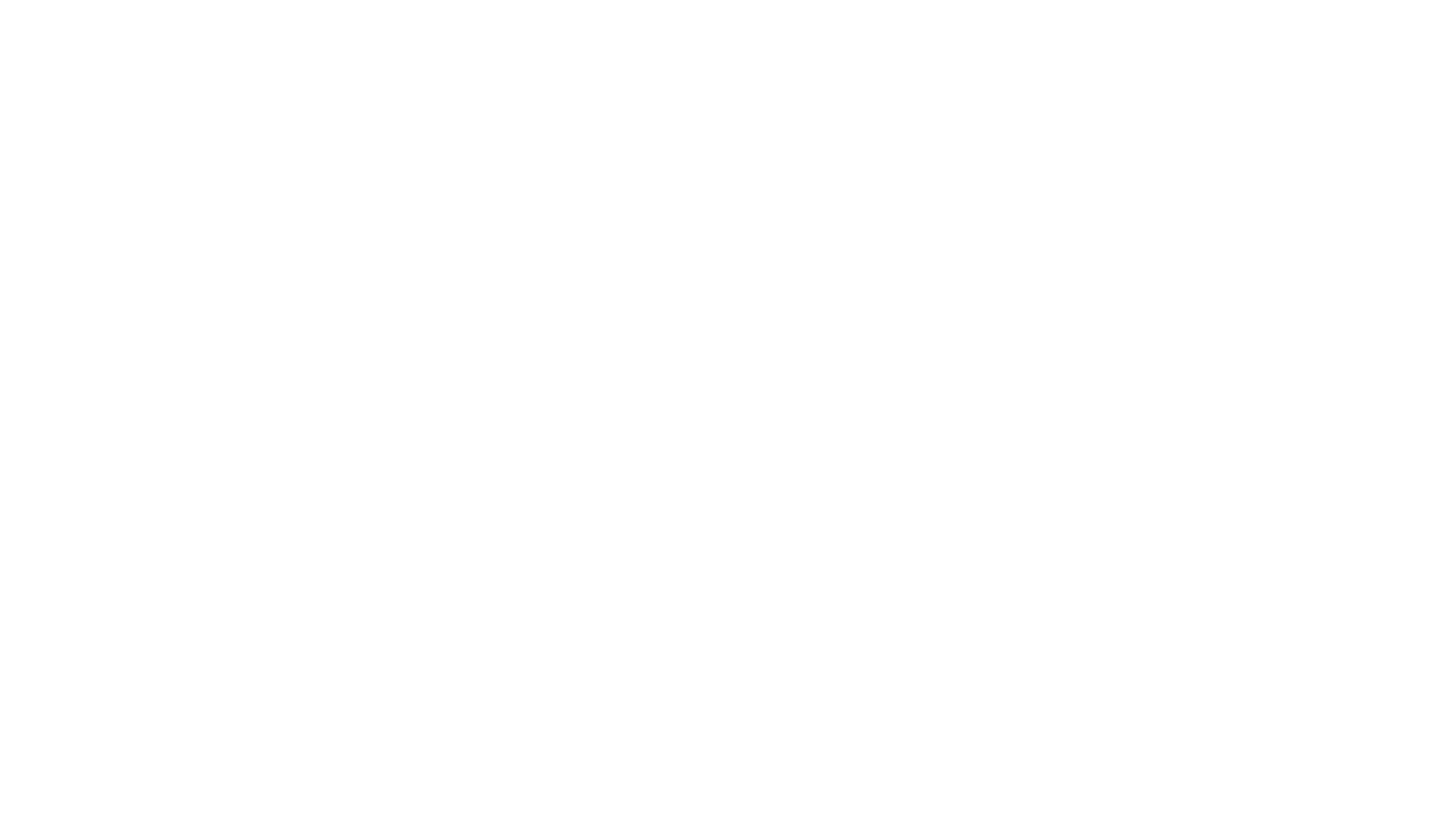

## Slide 10
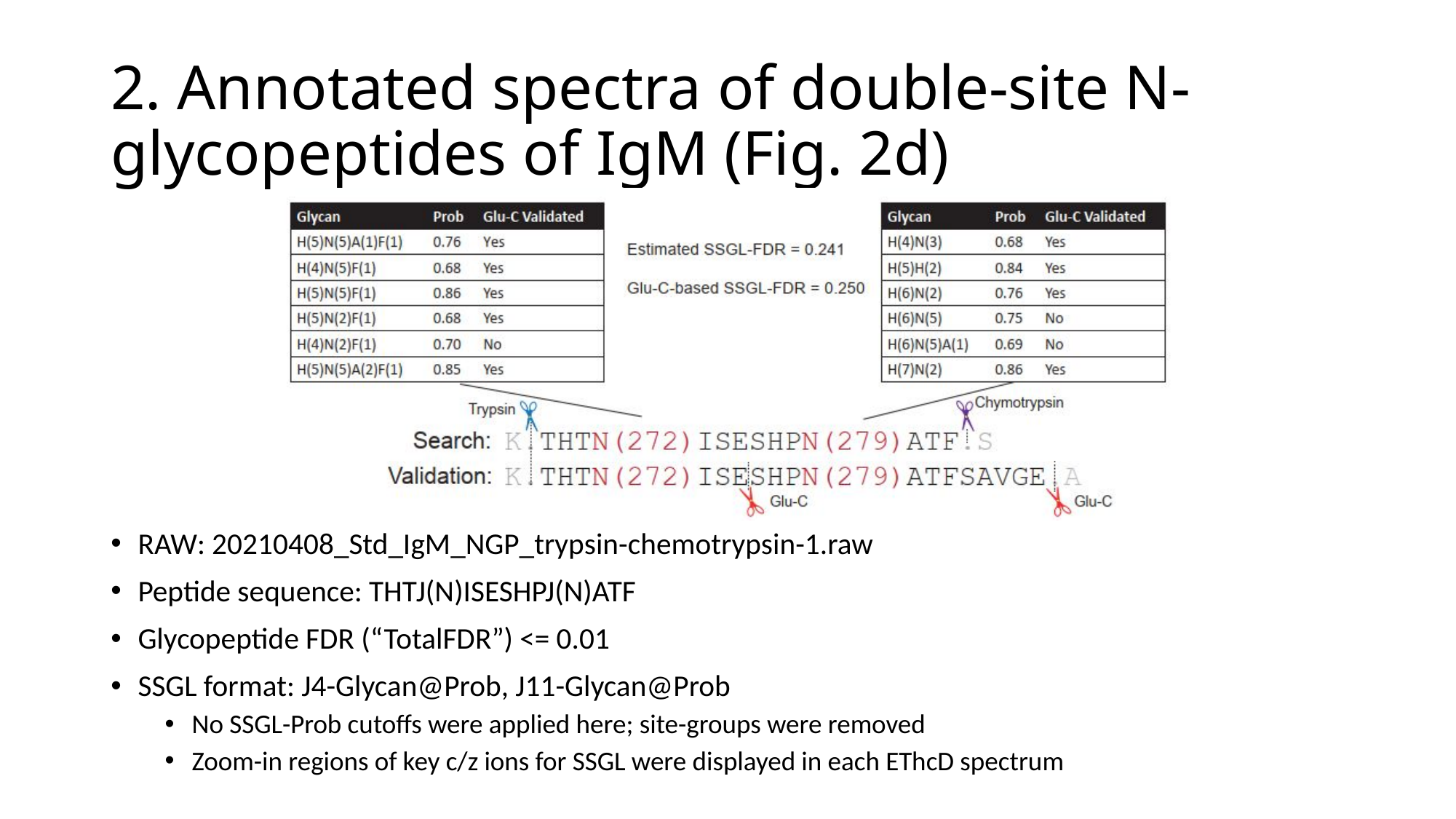

# 2. Annotated spectra of double-site N-glycopeptides of IgM (Fig. 2d)
RAW: 20210408_Std_IgM_NGP_trypsin-chemotrypsin-1.raw
Peptide sequence: THTJ(N)ISESHPJ(N)ATF
Glycopeptide FDR (“TotalFDR”) <= 0.01
SSGL format: J4-Glycan@Prob, J11-Glycan@Prob
No SSGL-Prob cutoffs were applied here; site-groups were removed
Zoom-in regions of key c/z ions for SSGL were displayed in each EThcD spectrum

## Slide 11
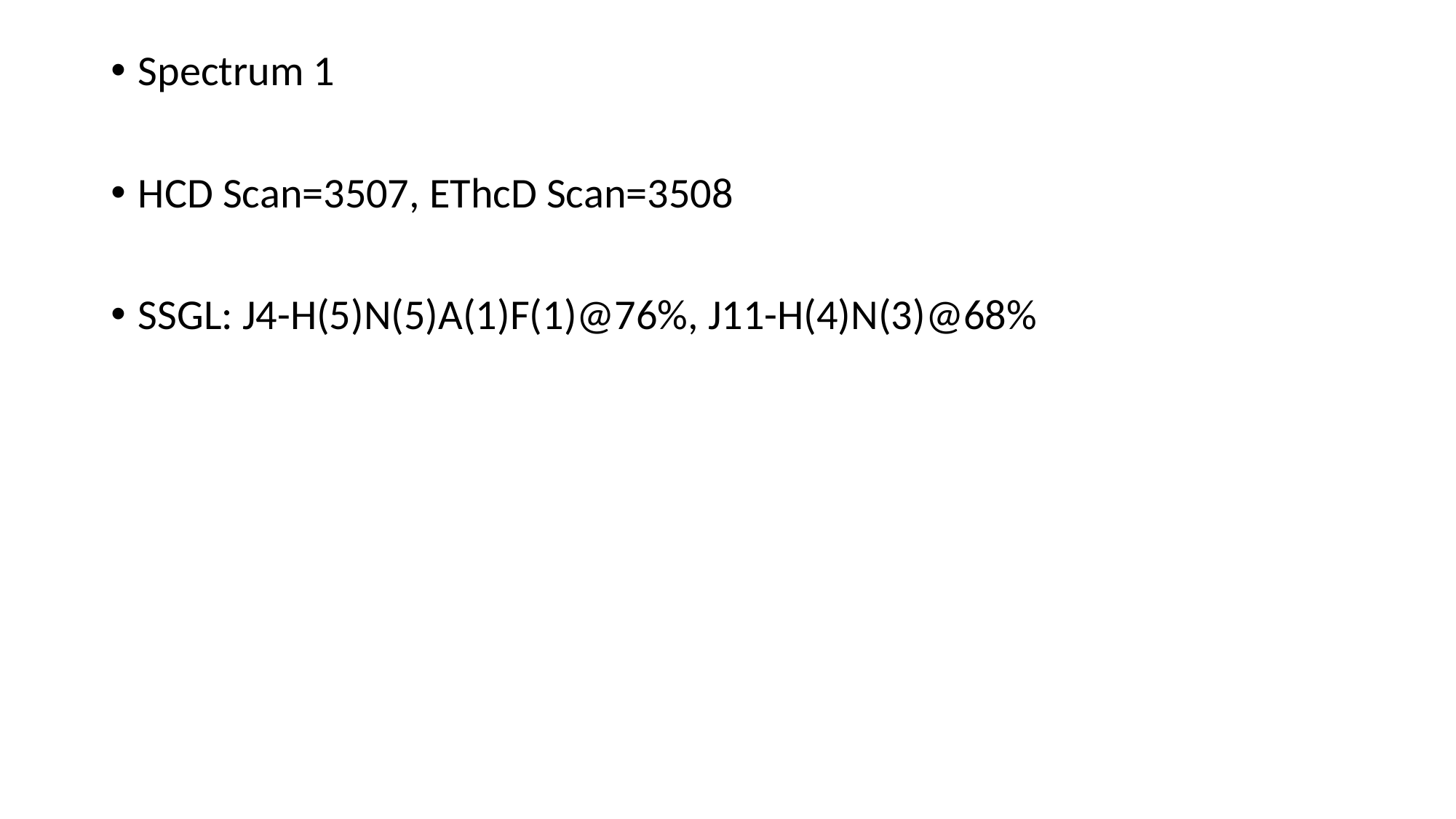

Spectrum 1
HCD Scan=3507, EThcD Scan=3508
SSGL: J4-H(5)N(5)A(1)F(1)@76%, J11-H(4)N(3)@68%

## Slide 12
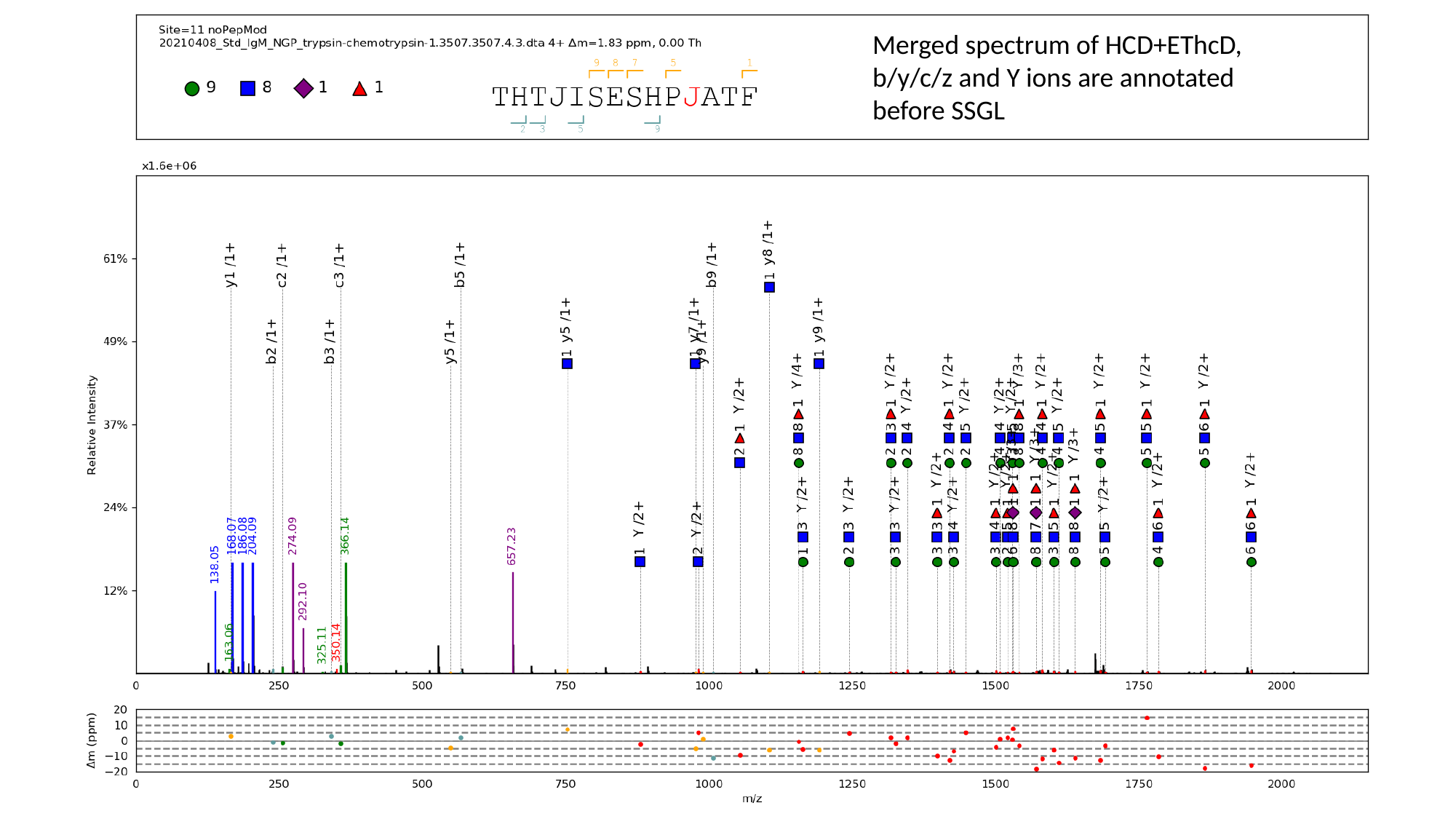

Merged spectrum of HCD+EThcD,
b/y/c/z and Y ions are annotated
before SSGL

## Slide 13
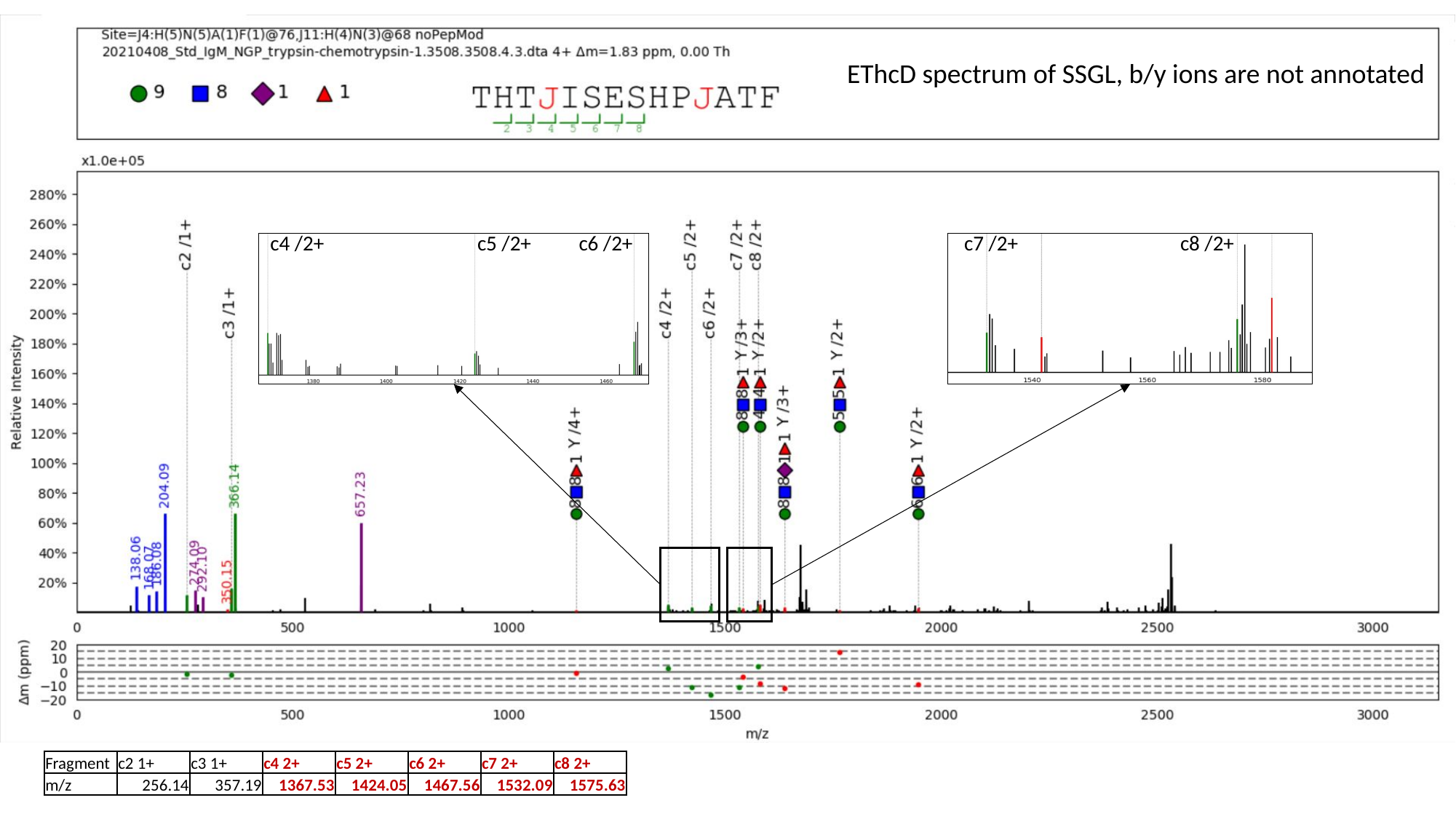

EThcD spectrum of SSGL, b/y ions are not annotated
c8 /2+
c4 /2+
c5 /2+
c6 /2+
c7 /2+
| Fragment | c2 1+ | c3 1+ | c4 2+ | c5 2+ | c6 2+ | c7 2+ | c8 2+ |
| --- | --- | --- | --- | --- | --- | --- | --- |
| m/z | 256.14 | 357.19 | 1367.53 | 1424.05 | 1467.56 | 1532.09 | 1575.63 |

## Slide 14
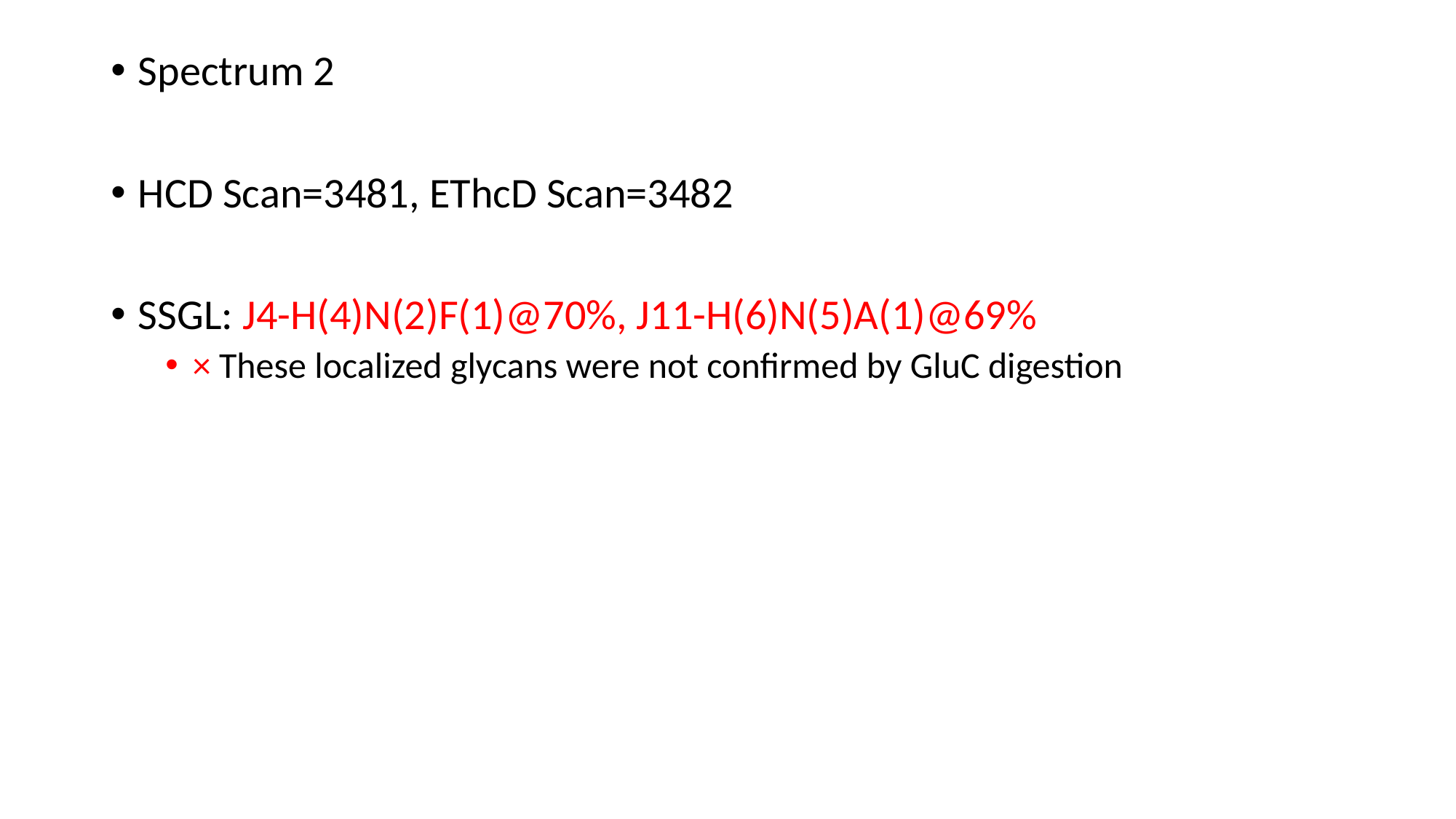

Spectrum 2
HCD Scan=3481, EThcD Scan=3482
SSGL: J4-H(4)N(2)F(1)@70%, J11-H(6)N(5)A(1)@69%
× These localized glycans were not confirmed by GluC digestion

## Slide 15
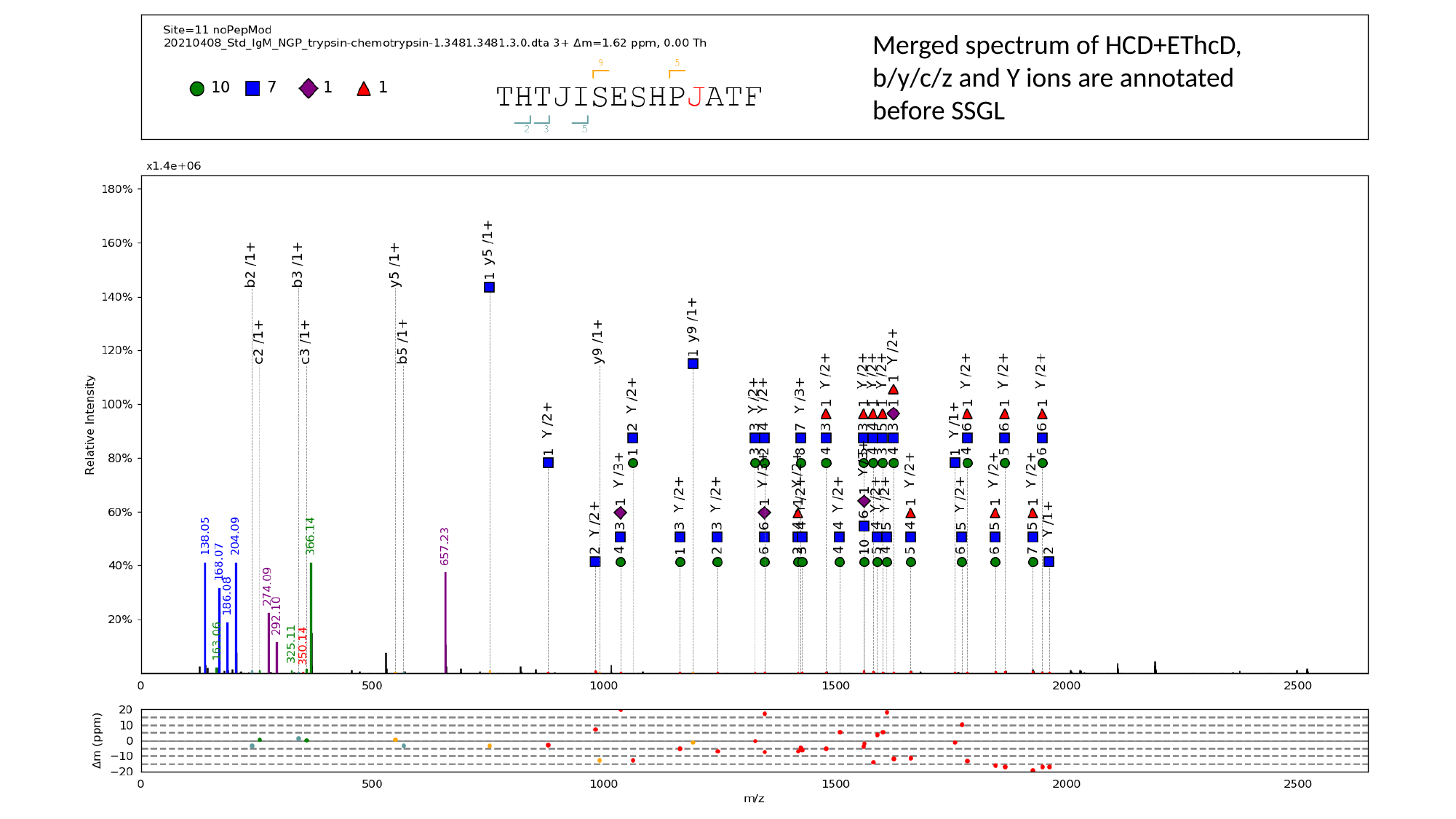

Merged spectrum of HCD+EThcD,
b/y/c/z and Y ions are annotated
before SSGL

## Slide 16
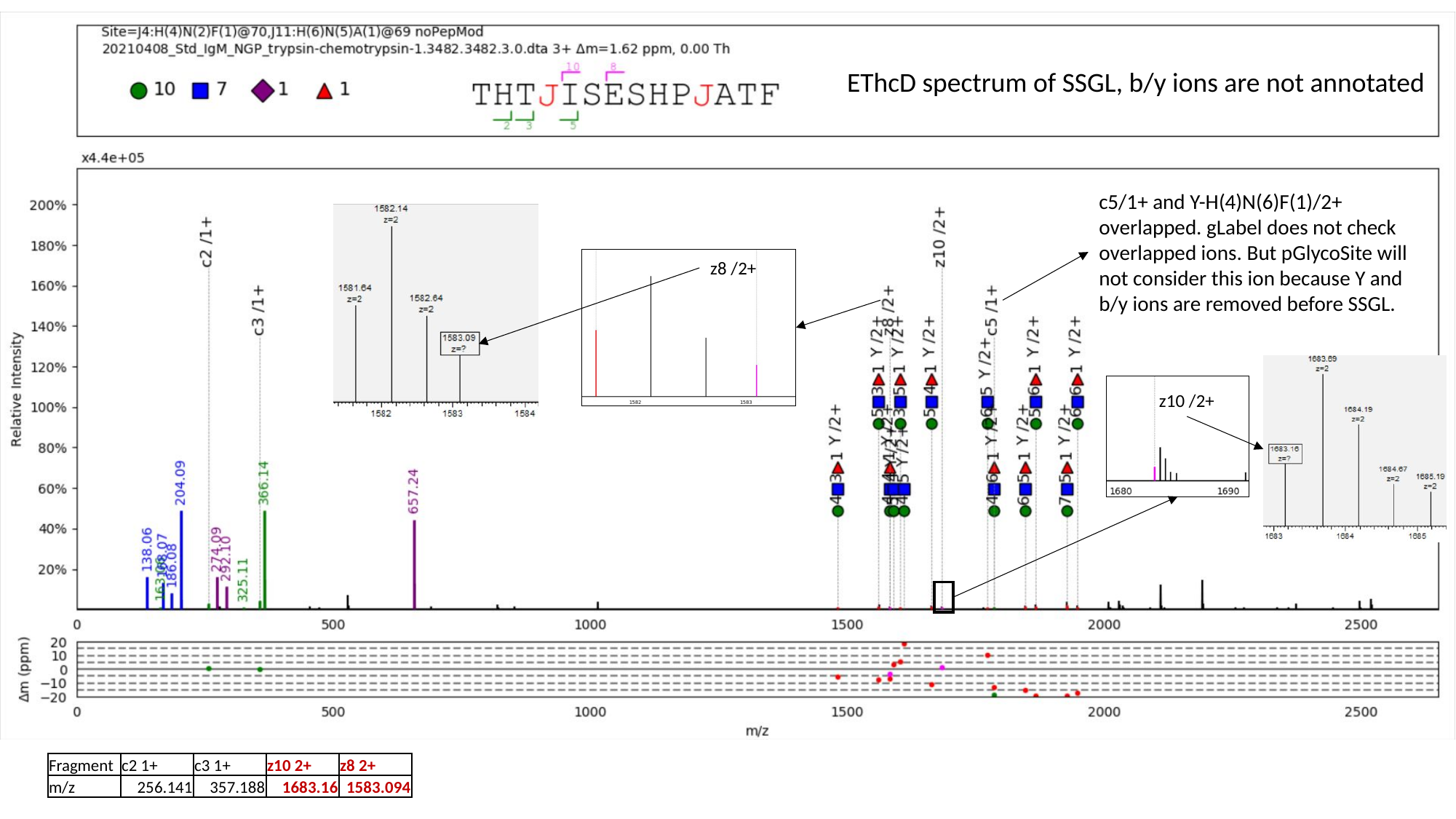

EThcD spectrum of SSGL, b/y ions are not annotated
c5/1+ and Y-H(4)N(6)F(1)/2+ overlapped. gLabel does not check overlapped ions. But pGlycoSite will not consider this ion because Y and b/y ions are removed before SSGL.
z8 /2+
z10 /2+
| Fragment | c2 1+ | c3 1+ | z10 2+ | z8 2+ |
| --- | --- | --- | --- | --- |
| m/z | 256.141 | 357.188 | 1683.16 | 1583.094 |

## Slide 17
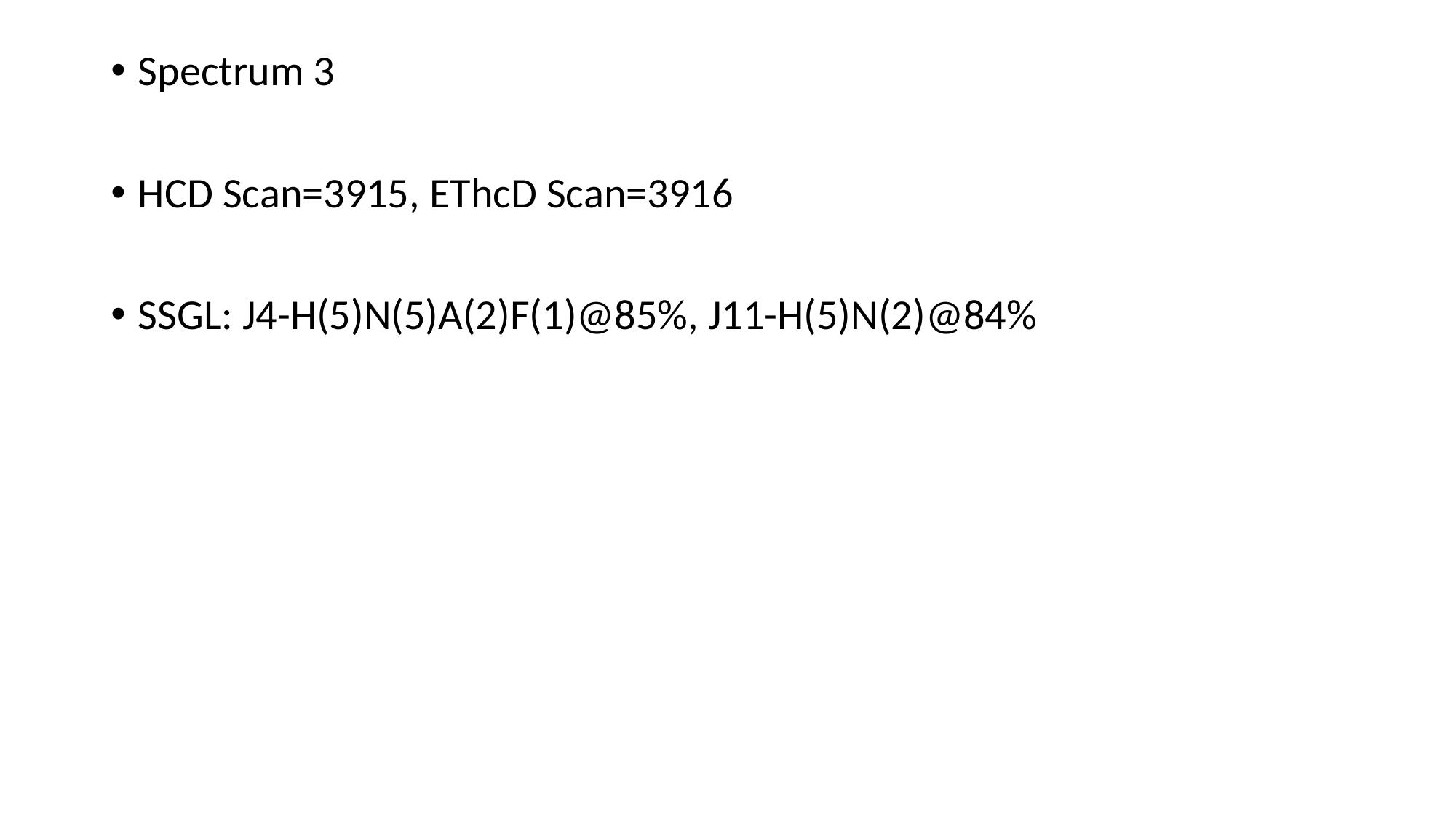

Spectrum 3
HCD Scan=3915, EThcD Scan=3916
SSGL: J4-H(5)N(5)A(2)F(1)@85%, J11-H(5)N(2)@84%

## Slide 18
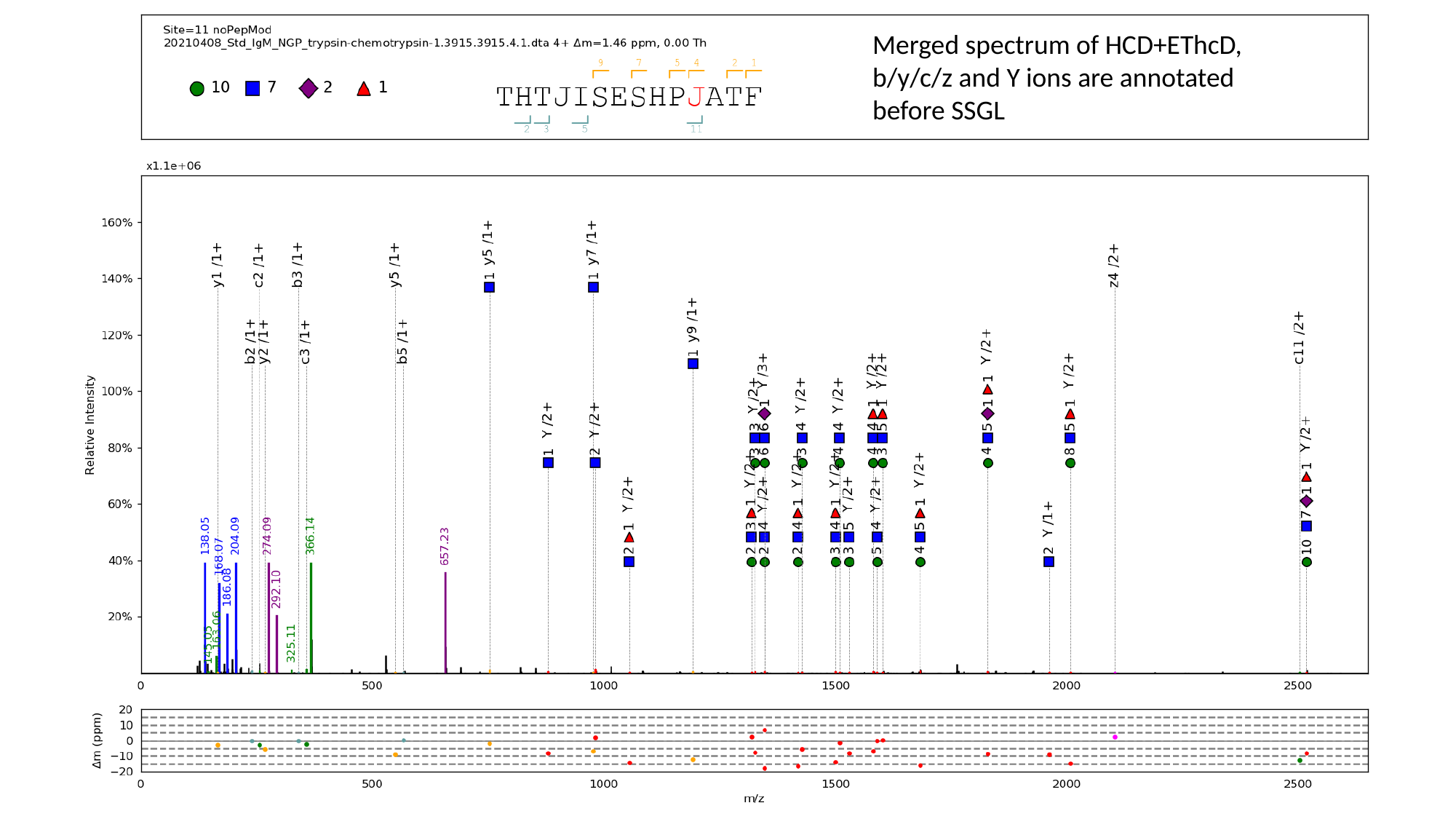

Merged spectrum of HCD+EThcD,
b/y/c/z and Y ions are annotated
before SSGL

## Slide 19
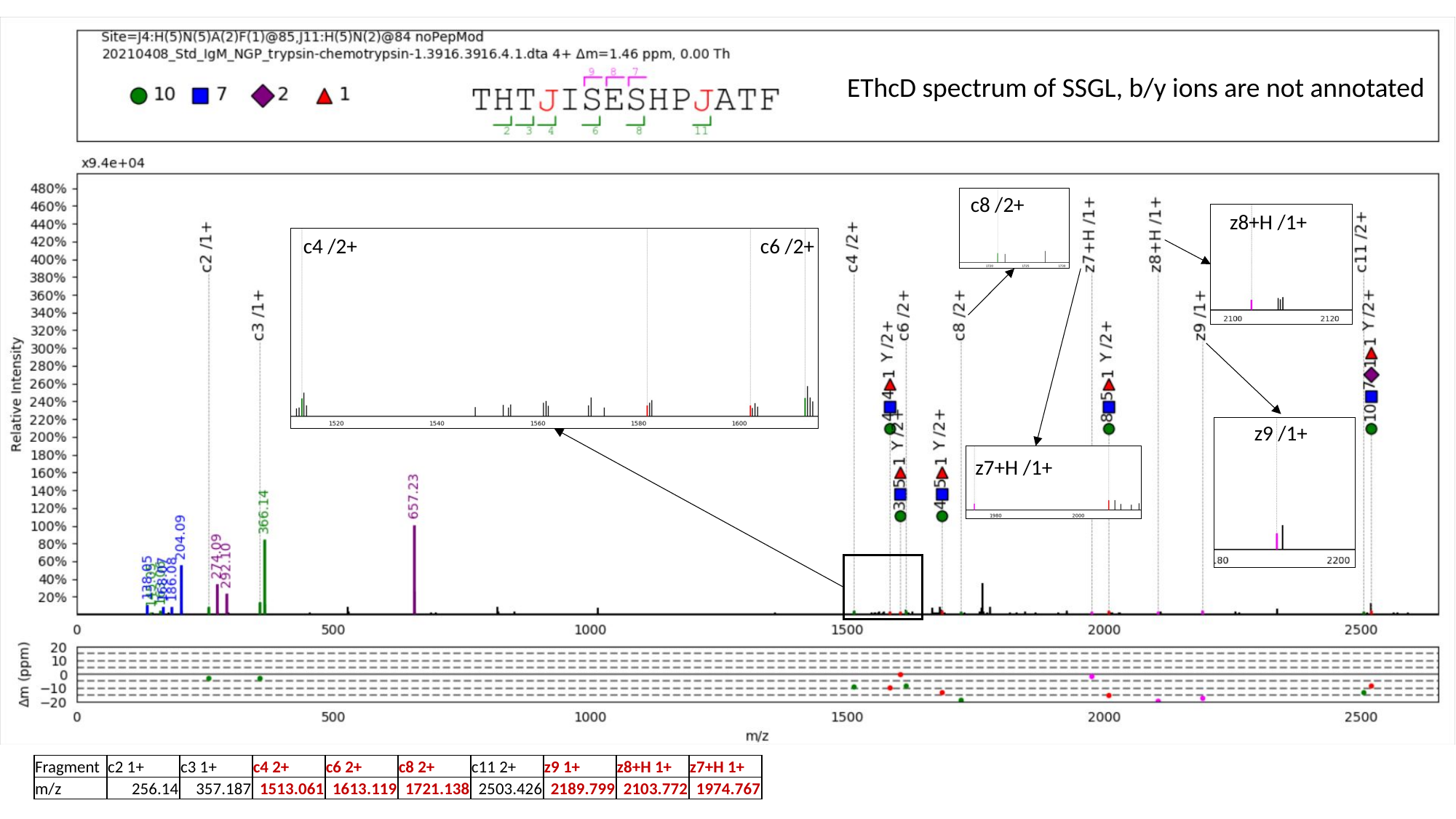

EThcD spectrum of SSGL, b/y ions are not annotated
c8 /2+
z8+H /1+
c4 /2+
c6 /2+
z9 /1+
z7+H /1+
| Fragment | c2 1+ | c3 1+ | c4 2+ | c6 2+ | c8 2+ | c11 2+ | z9 1+ | z8+H 1+ | z7+H 1+ |
| --- | --- | --- | --- | --- | --- | --- | --- | --- | --- |
| m/z | 256.14 | 357.187 | 1513.061 | 1613.119 | 1721.138 | 2503.426 | 2189.799 | 2103.772 | 1974.767 |

## Slide 20
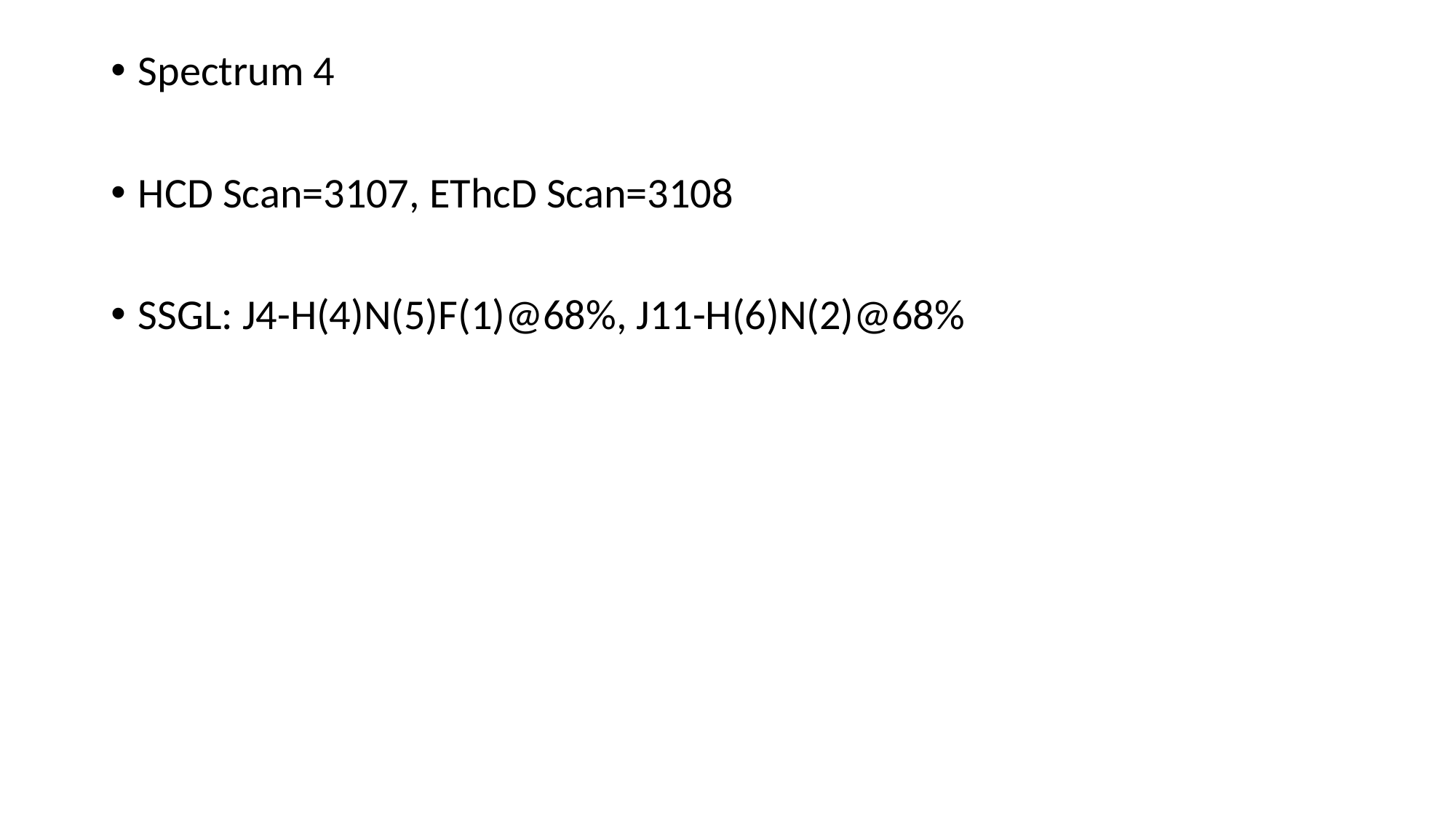

Spectrum 4
HCD Scan=3107, EThcD Scan=3108
SSGL: J4-H(4)N(5)F(1)@68%, J11-H(6)N(2)@68%

## Slide 21
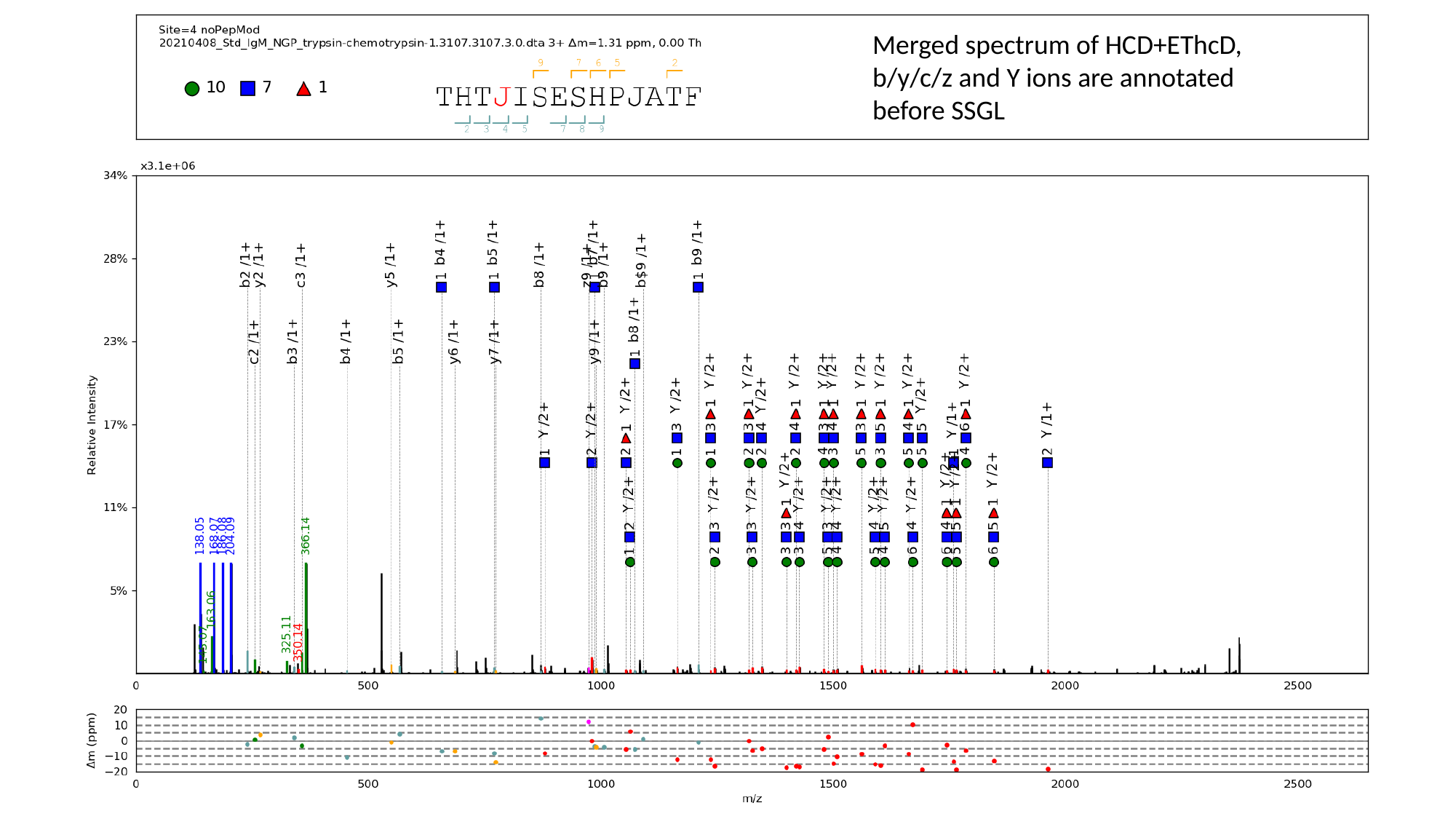

Merged spectrum of HCD+EThcD,
b/y/c/z and Y ions are annotated
before SSGL

## Slide 22
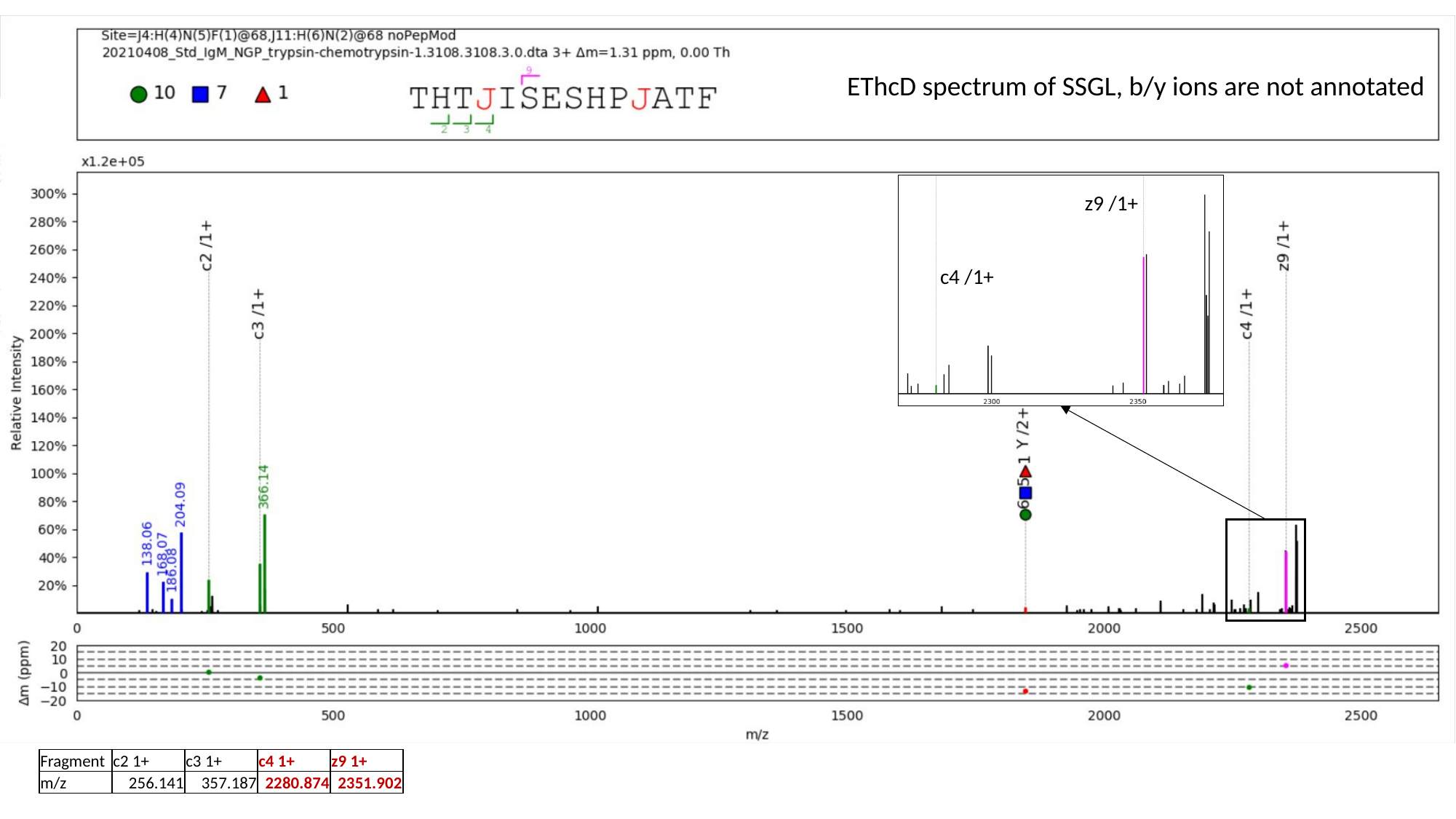

EThcD spectrum of SSGL, b/y ions are not annotated
z9 /1+
c4 /1+
| Fragment | c2 1+ | c3 1+ | c4 1+ | z9 1+ |
| --- | --- | --- | --- | --- |
| m/z | 256.141 | 357.187 | 2280.874 | 2351.902 |

## Slide 23
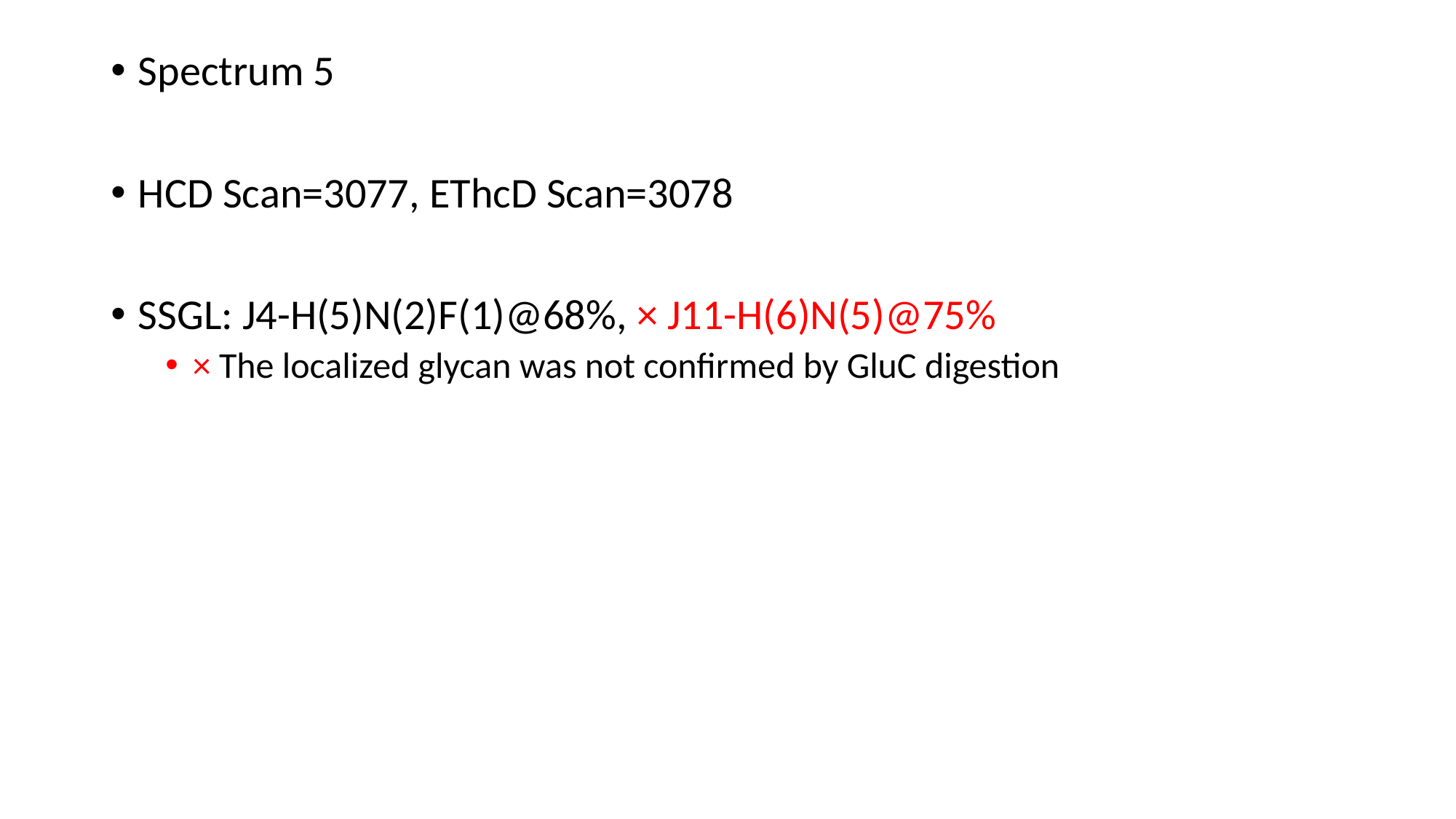

Spectrum 5
HCD Scan=3077, EThcD Scan=3078
SSGL: J4-H(5)N(2)F(1)@68%, × J11-H(6)N(5)@75%
× The localized glycan was not confirmed by GluC digestion

## Slide 24
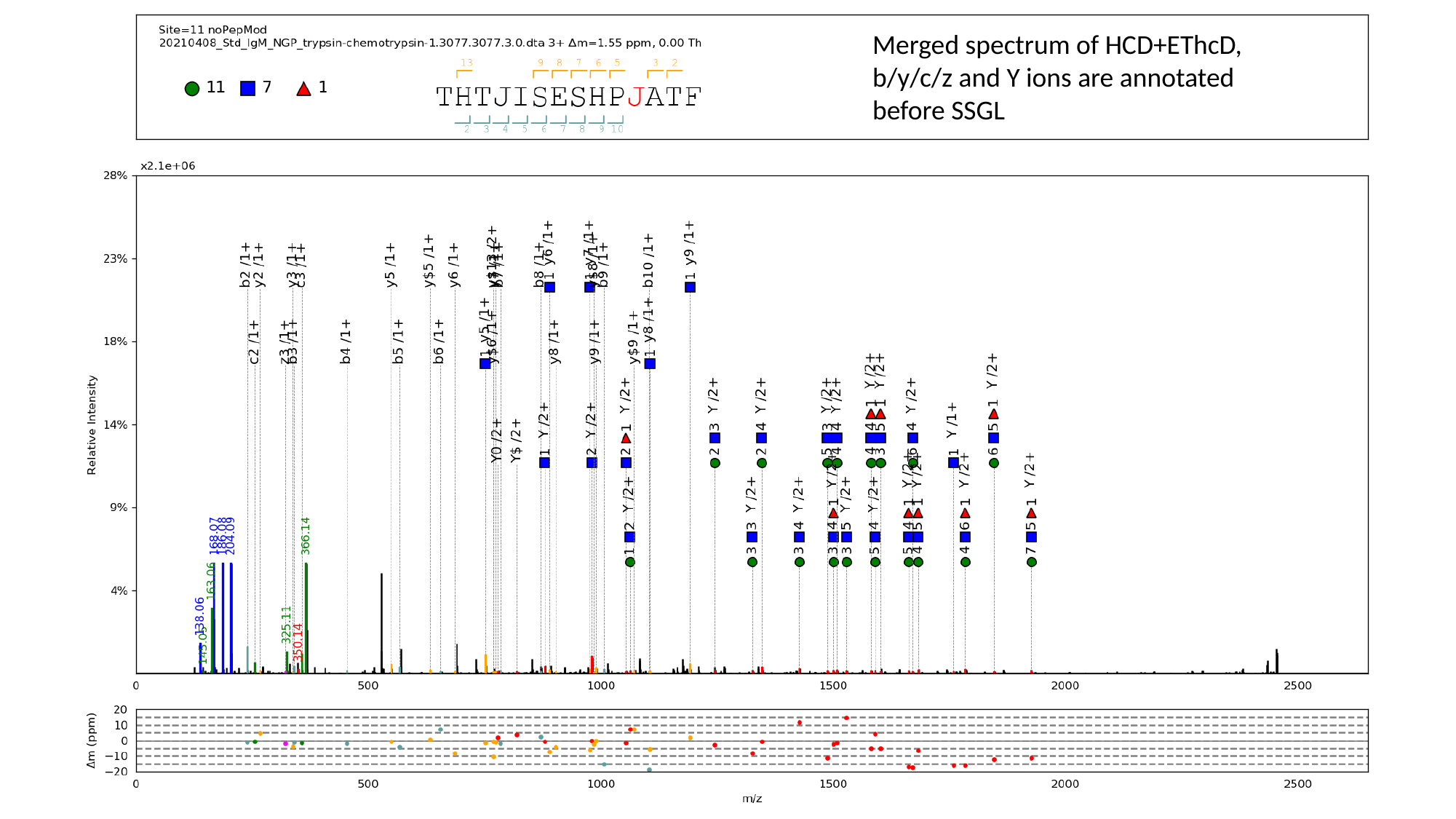

Merged spectrum of HCD+EThcD,
b/y/c/z and Y ions are annotated
before SSGL

## Slide 25
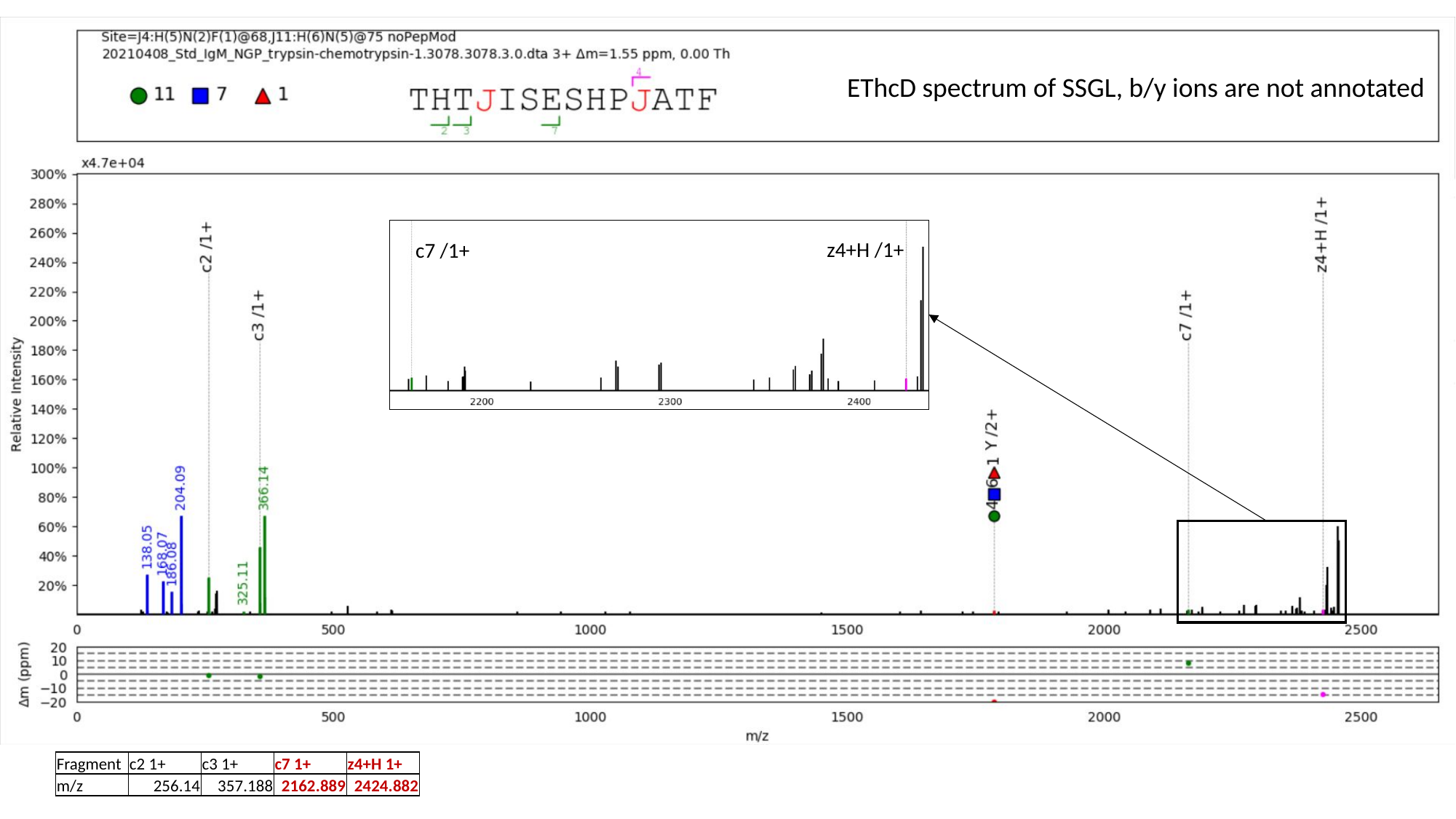

EThcD spectrum of SSGL, b/y ions are not annotated
z4+H /1+
c7 /1+
| Fragment | c2 1+ | c3 1+ | c7 1+ | z4+H 1+ |
| --- | --- | --- | --- | --- |
| m/z | 256.14 | 357.188 | 2162.889 | 2424.882 |

## Slide 26
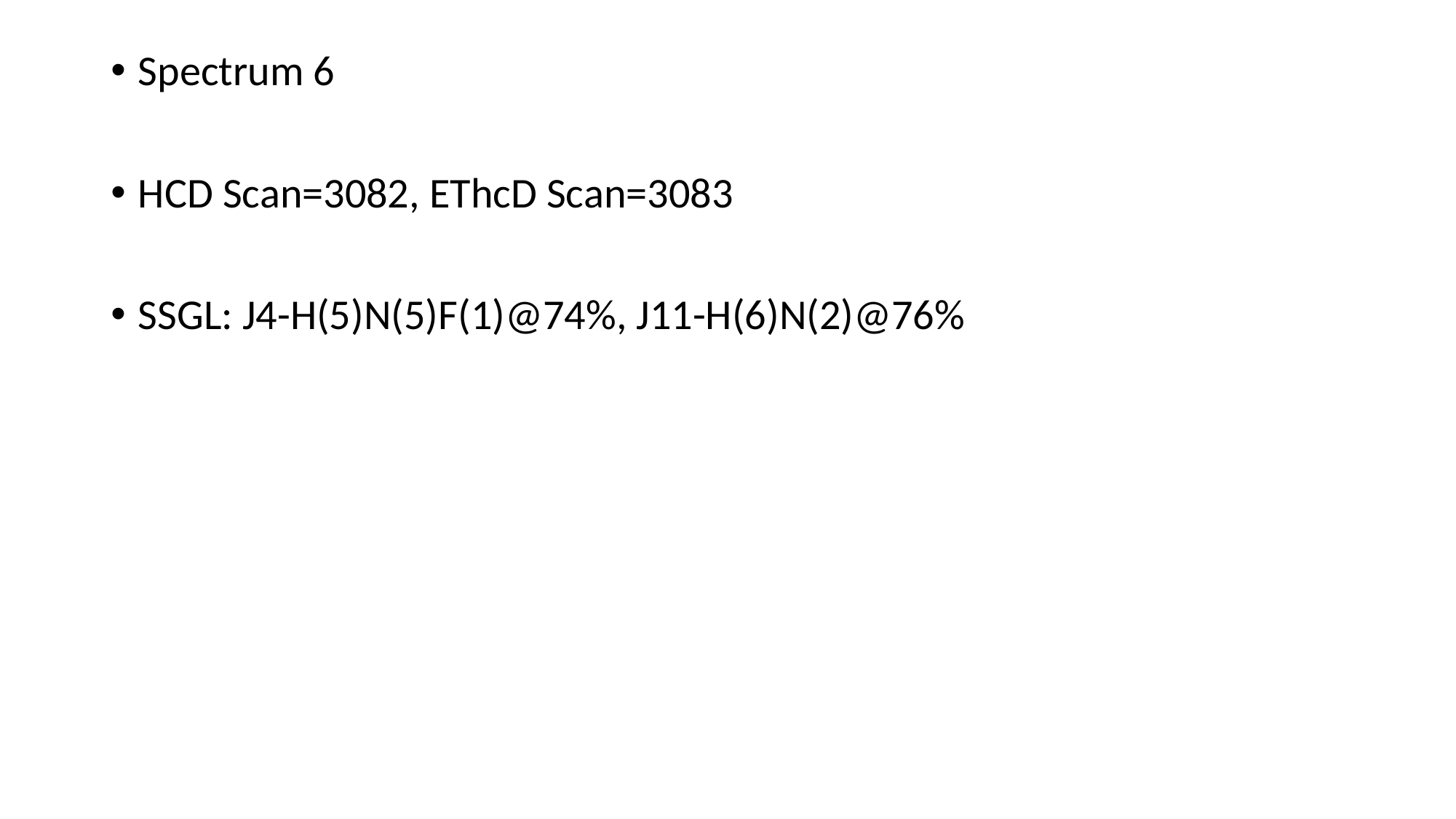

Spectrum 6
HCD Scan=3082, EThcD Scan=3083
SSGL: J4-H(5)N(5)F(1)@74%, J11-H(6)N(2)@76%

## Slide 27
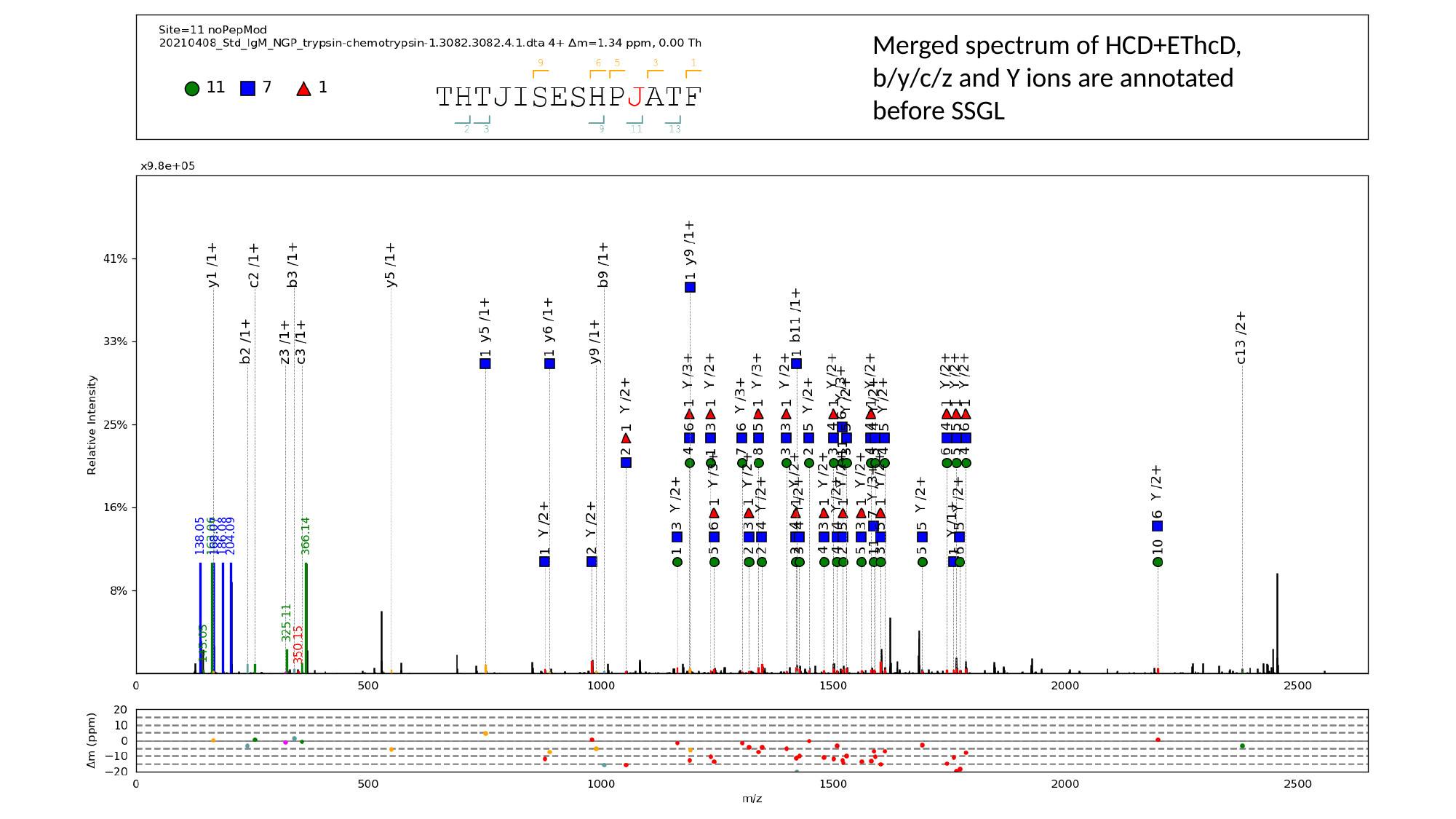

Merged spectrum of HCD+EThcD,
b/y/c/z and Y ions are annotated
before SSGL

## Slide 28
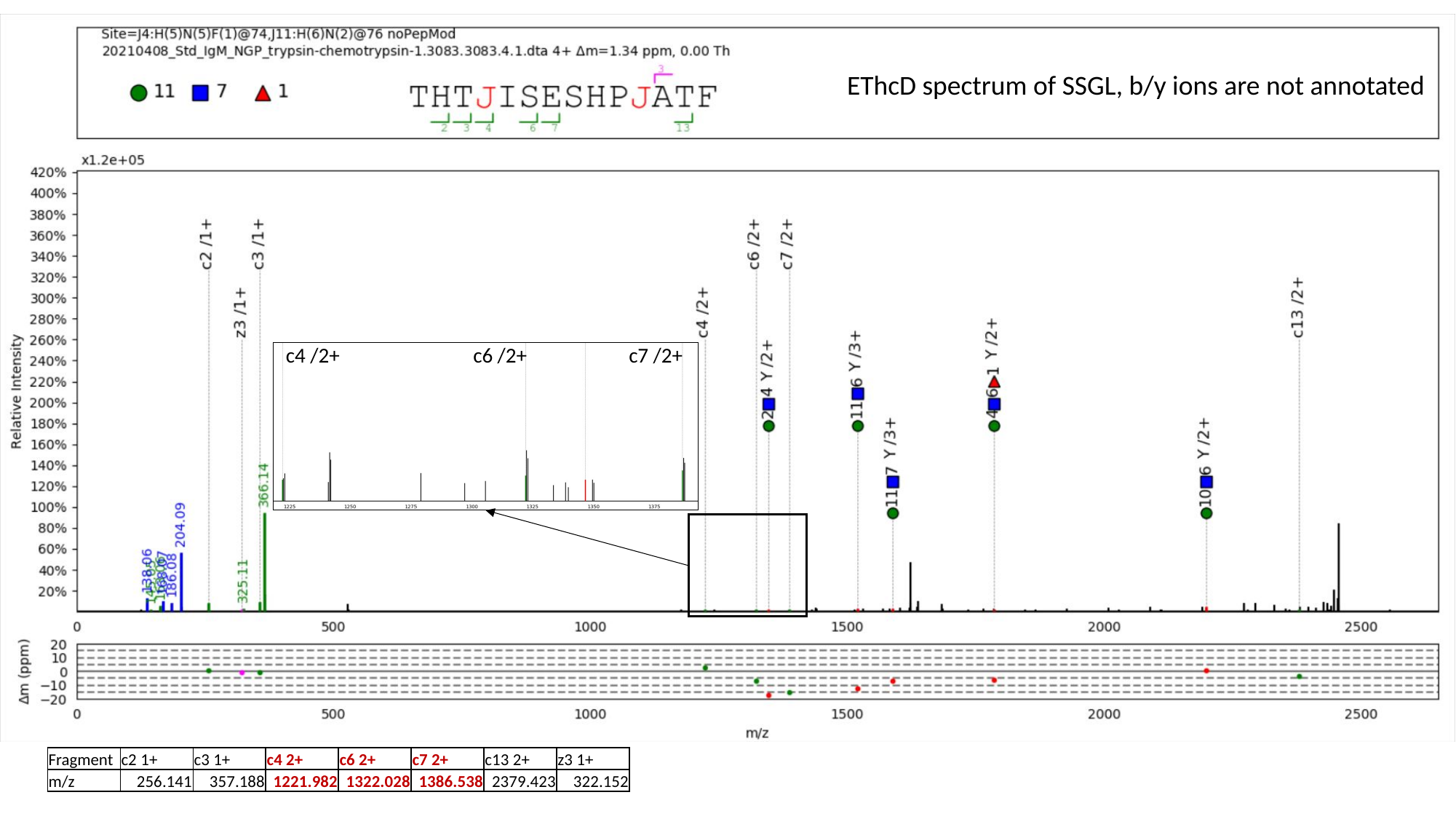

EThcD spectrum of SSGL, b/y ions are not annotated
c7 /2+
c4 /2+
c6 /2+
| Fragment | c2 1+ | c3 1+ | c4 2+ | c6 2+ | c7 2+ | c13 2+ | z3 1+ |
| --- | --- | --- | --- | --- | --- | --- | --- |
| m/z | 256.141 | 357.188 | 1221.982 | 1322.028 | 1386.538 | 2379.423 | 322.152 |

## Slide 29
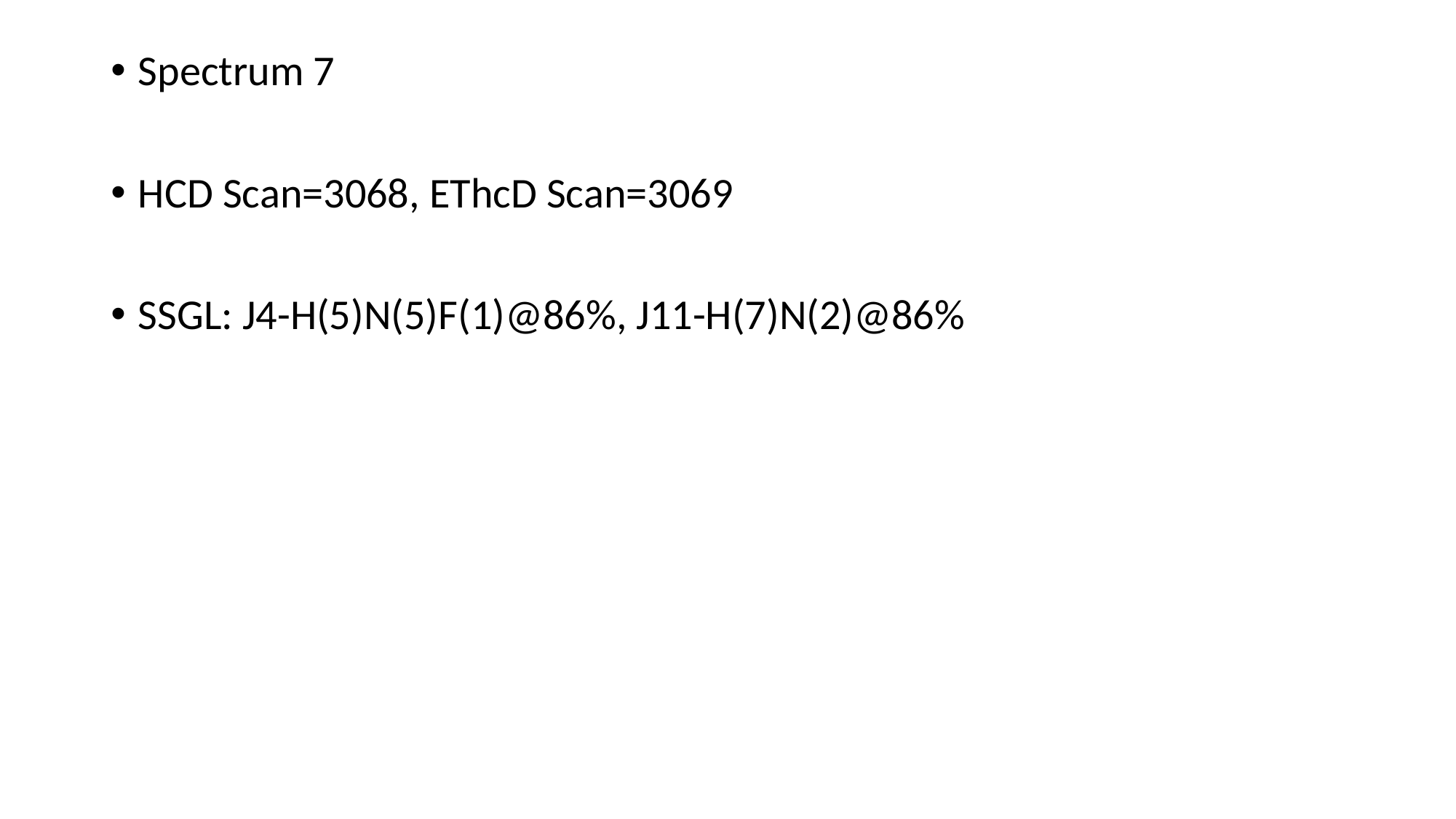

Spectrum 7
HCD Scan=3068, EThcD Scan=3069
SSGL: J4-H(5)N(5)F(1)@86%, J11-H(7)N(2)@86%

## Slide 30
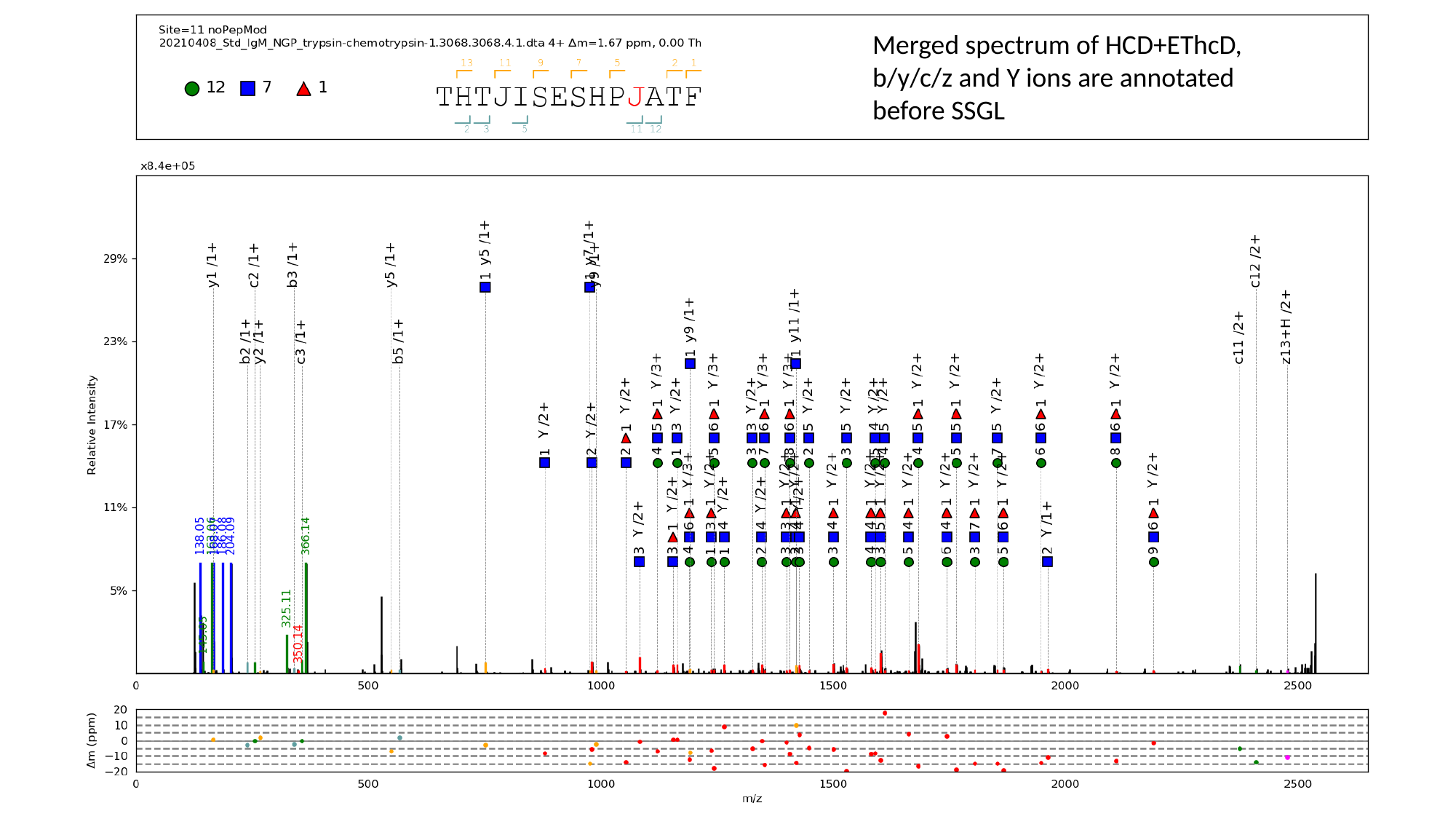

Merged spectrum of HCD+EThcD,
b/y/c/z and Y ions are annotated
before SSGL

## Slide 31
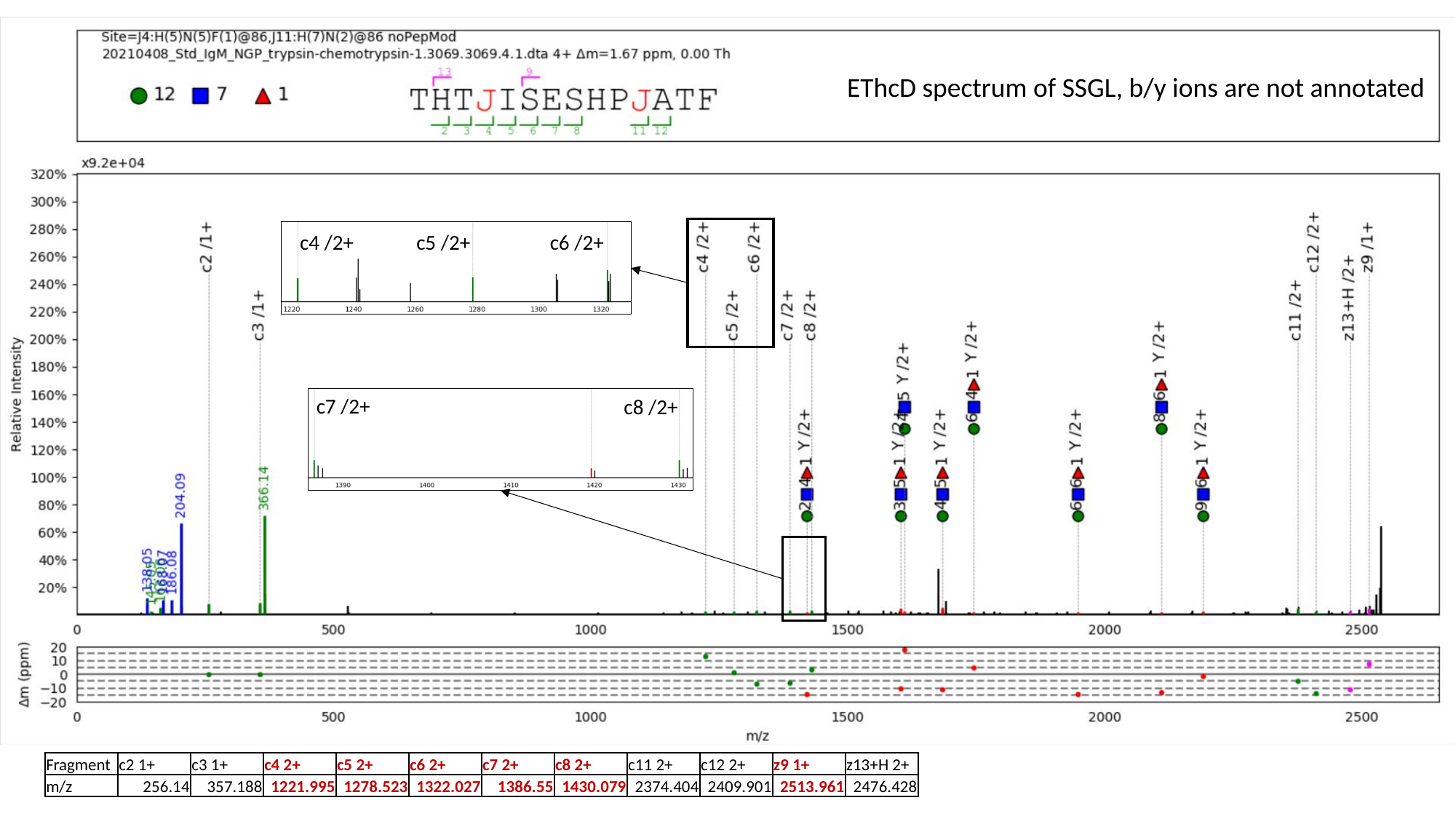

EThcD spectrum of SSGL, b/y ions are not annotated
c6 /2+
c5 /2+
c4 /2+
c7 /2+
c8 /2+
| Fragment | c2 1+ | c3 1+ | c4 2+ | c5 2+ | c6 2+ | c7 2+ | c8 2+ | c11 2+ | c12 2+ | z9 1+ | z13+H 2+ |
| --- | --- | --- | --- | --- | --- | --- | --- | --- | --- | --- | --- |
| m/z | 256.14 | 357.188 | 1221.995 | 1278.523 | 1322.027 | 1386.55 | 1430.079 | 2374.404 | 2409.901 | 2513.961 | 2476.428 |

## Slide 32
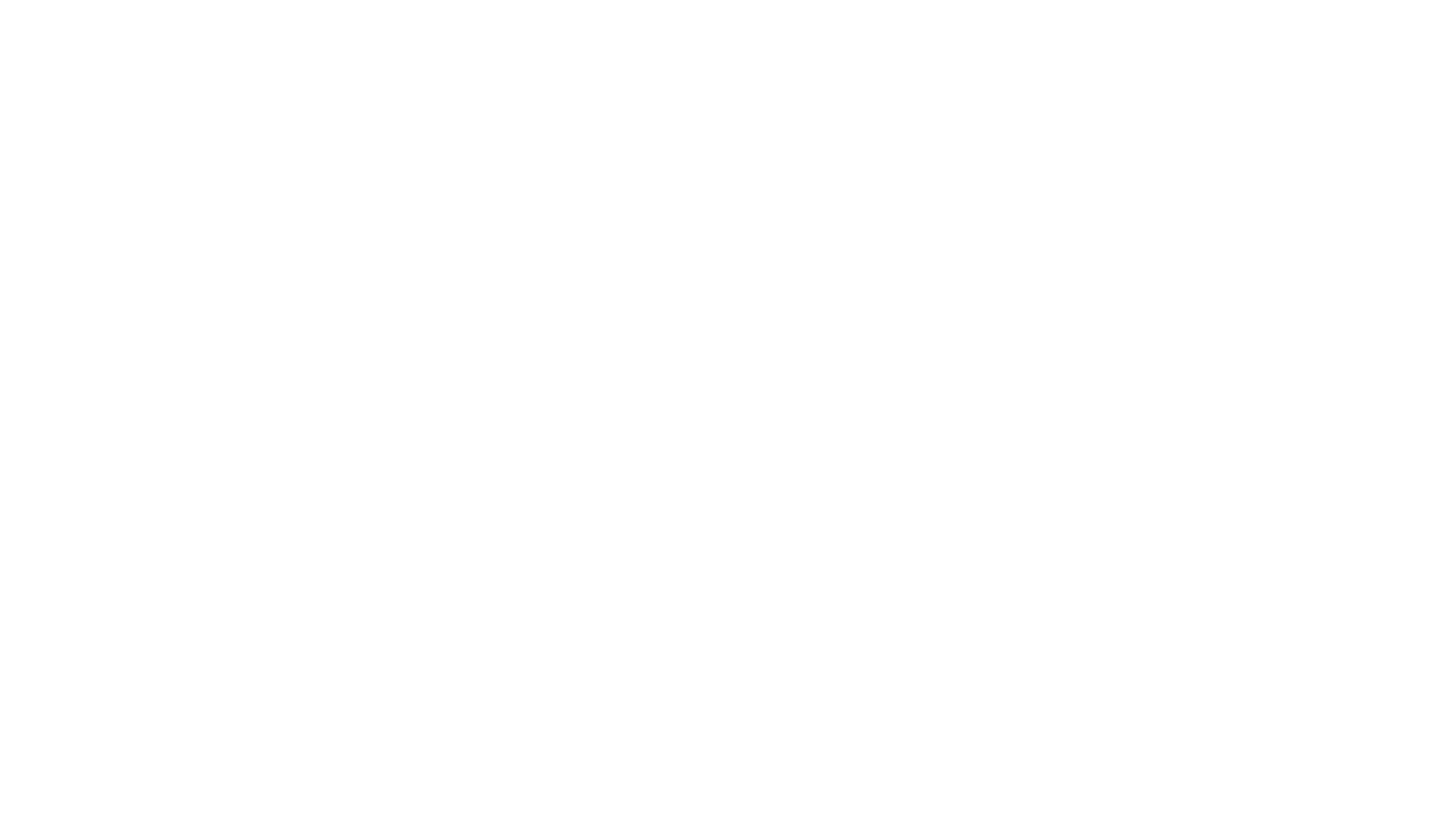

## Slide 33
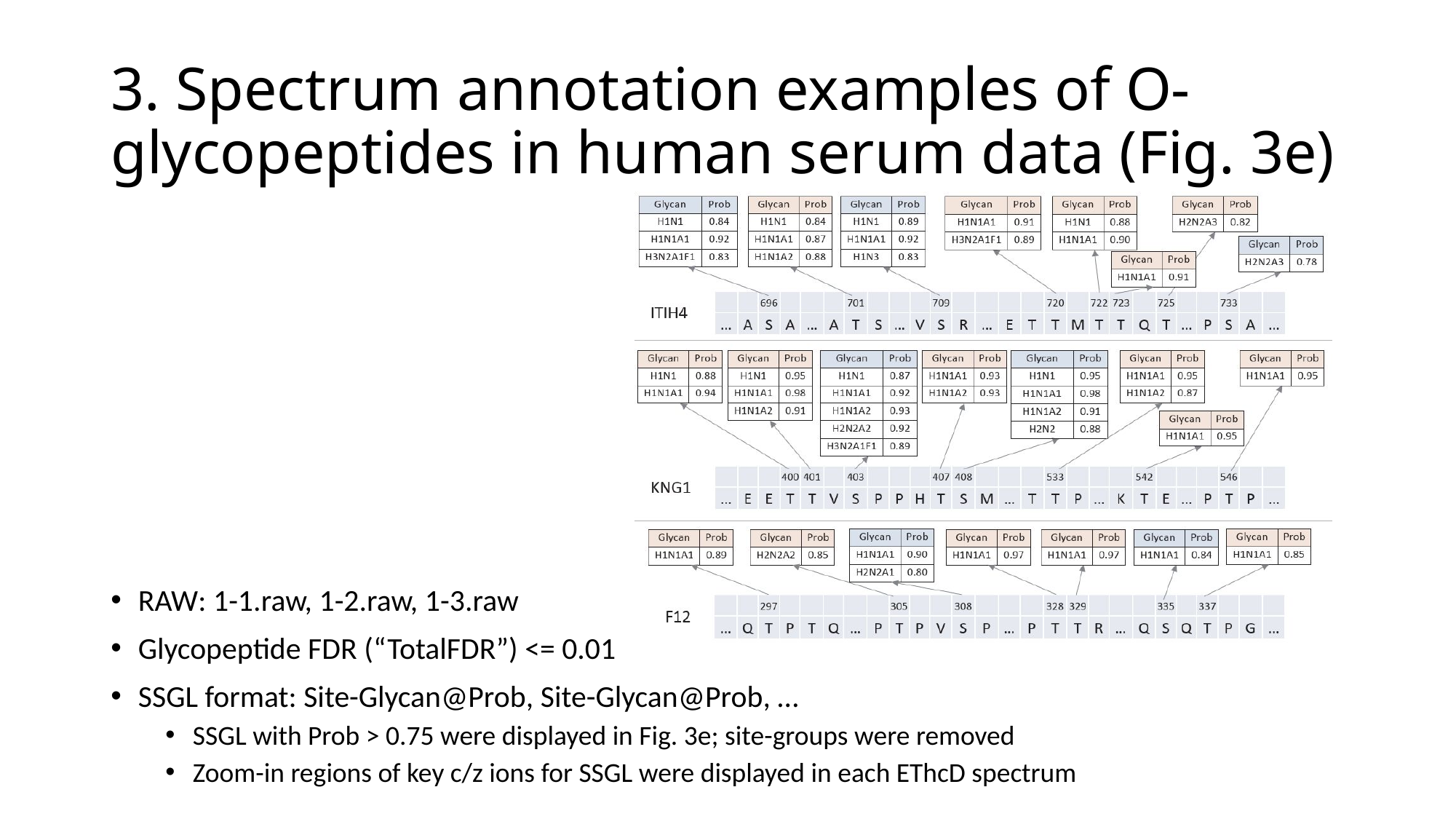

# 3. Spectrum annotation examples of O-glycopeptides in human serum data (Fig. 3e)
RAW: 1-1.raw, 1-2.raw, 1-3.raw
Glycopeptide FDR (“TotalFDR”) <= 0.01
SSGL format: Site-Glycan@Prob, Site-Glycan@Prob, …
SSGL with Prob > 0.75 were displayed in Fig. 3e; site-groups were removed
Zoom-in regions of key c/z ions for SSGL were displayed in each EThcD spectrum

## Slide 34
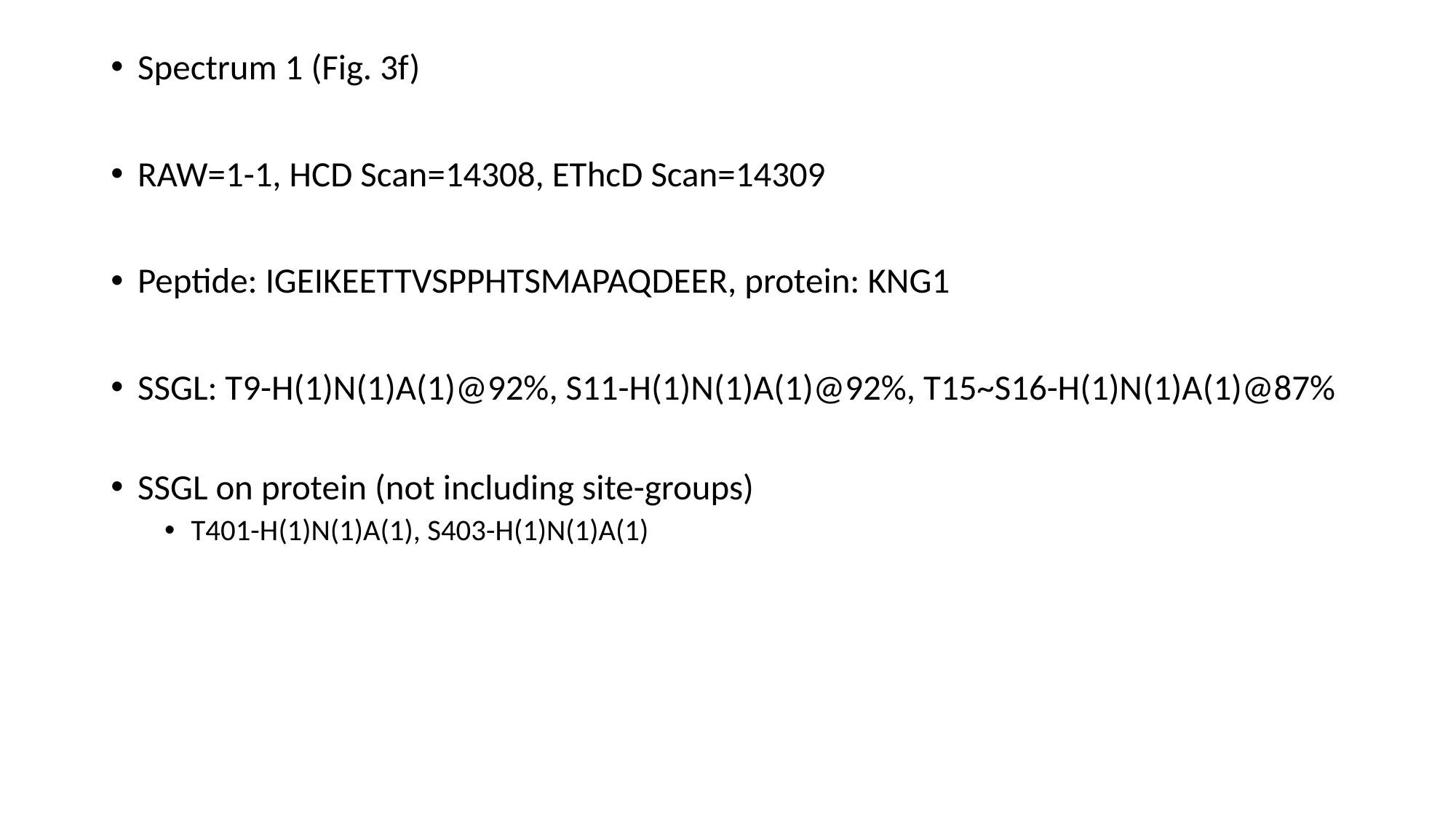

Spectrum 1 (Fig. 3f)
RAW=1-1, HCD Scan=14308, EThcD Scan=14309
Peptide: IGEIKEETTVSPPHTSMAPAQDEER, protein: KNG1
SSGL: T9-H(1)N(1)A(1)@92%, S11-H(1)N(1)A(1)@92%, T15~S16-H(1)N(1)A(1)@87%
SSGL on protein (not including site-groups)
T401-H(1)N(1)A(1), S403-H(1)N(1)A(1)

## Slide 35
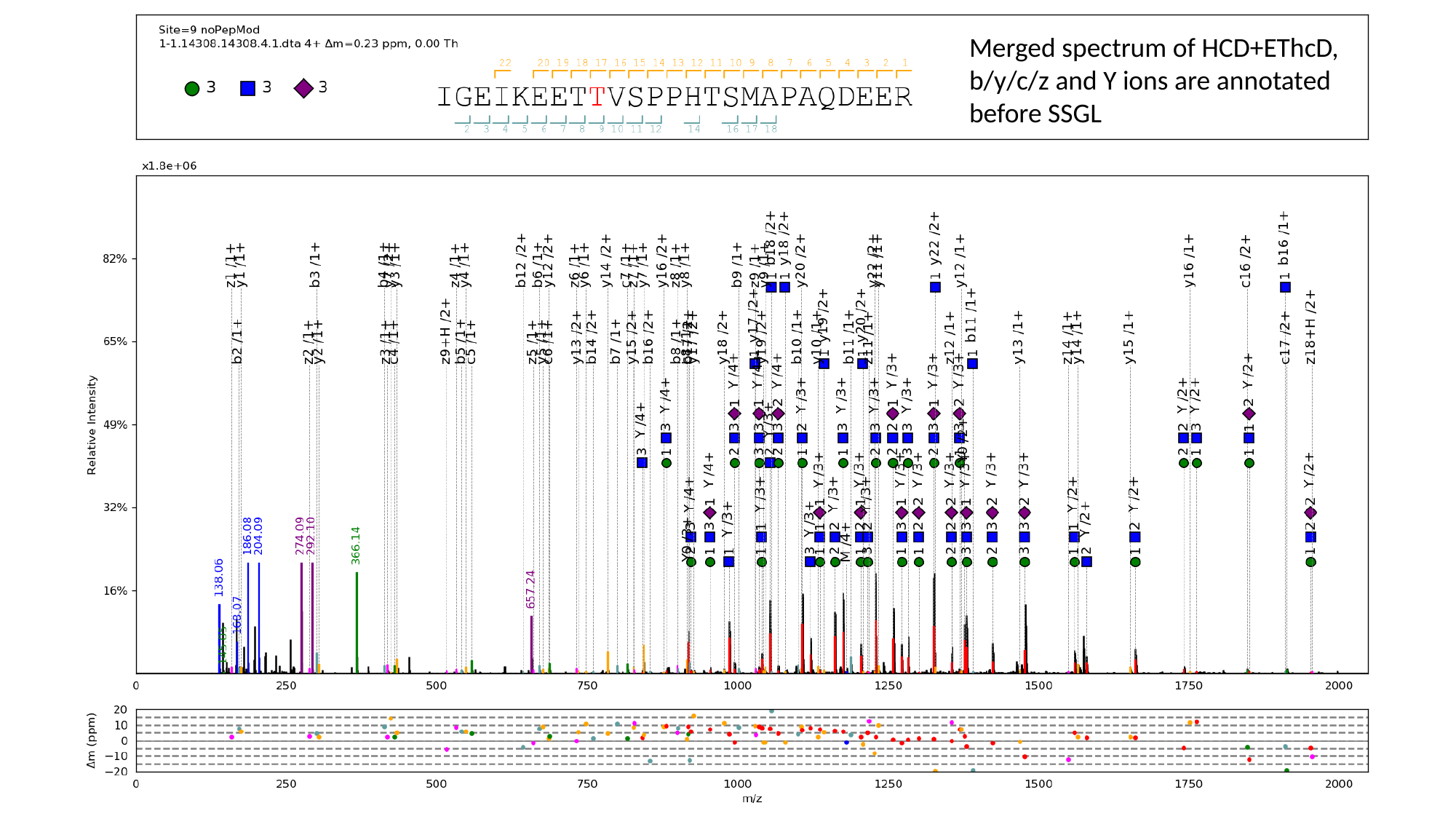

Merged spectrum of HCD+EThcD,
b/y/c/z and Y ions are annotated
before SSGL

## Slide 36
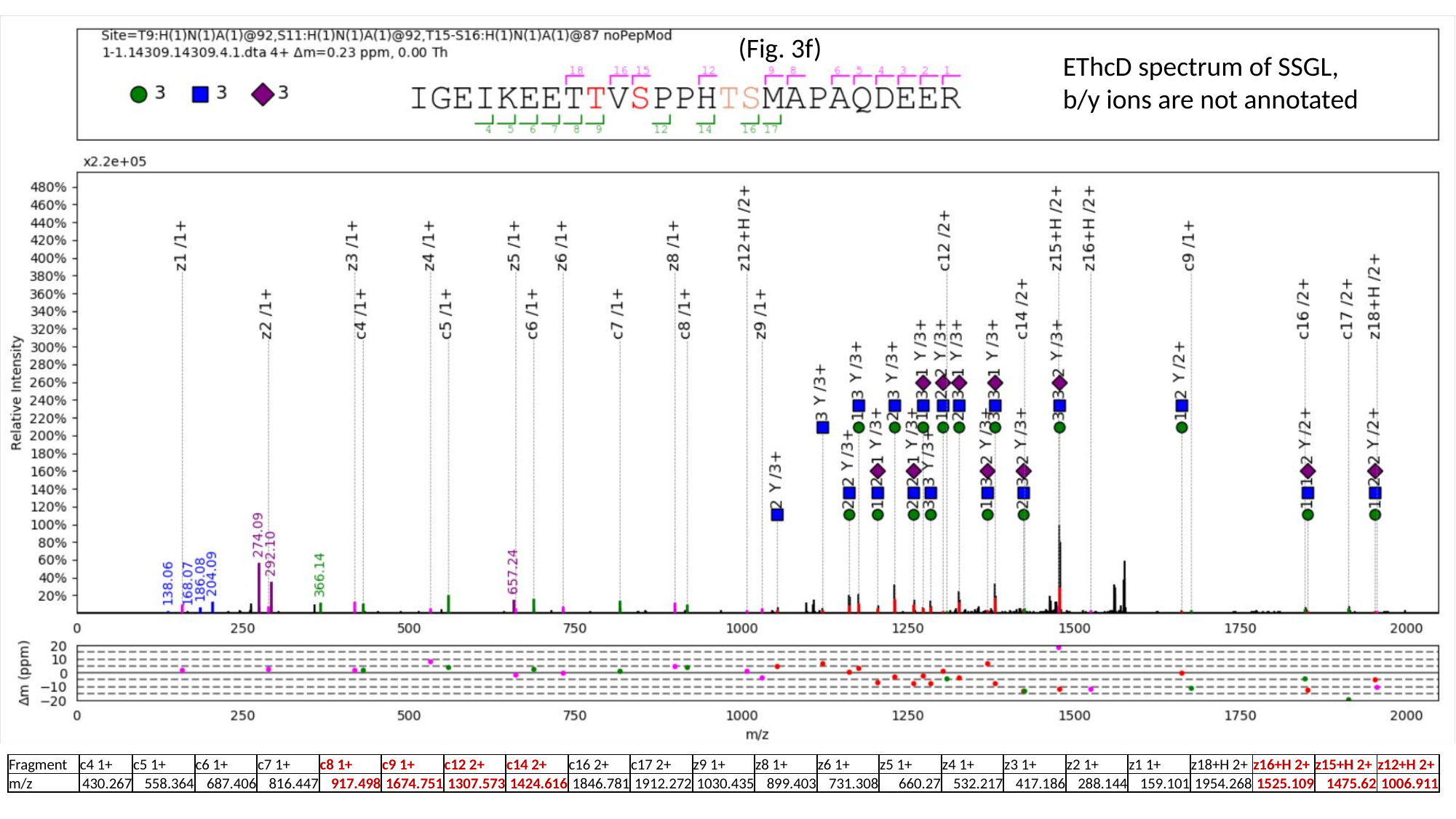

(Fig. 3f)
EThcD spectrum of SSGL, b/y ions are not annotated
| Fragment | c4 1+ | c5 1+ | c6 1+ | c7 1+ | c8 1+ | c9 1+ | c12 2+ | c14 2+ | c16 2+ | c17 2+ | z9 1+ | z8 1+ | z6 1+ | z5 1+ | z4 1+ | z3 1+ | z2 1+ | z1 1+ | z18+H 2+ | z16+H 2+ | z15+H 2+ | z12+H 2+ |
| --- | --- | --- | --- | --- | --- | --- | --- | --- | --- | --- | --- | --- | --- | --- | --- | --- | --- | --- | --- | --- | --- | --- |
| m/z | 430.267 | 558.364 | 687.406 | 816.447 | 917.498 | 1674.751 | 1307.573 | 1424.616 | 1846.781 | 1912.272 | 1030.435 | 899.403 | 731.308 | 660.27 | 532.217 | 417.186 | 288.144 | 159.101 | 1954.268 | 1525.109 | 1475.62 | 1006.911 |

## Slide 37
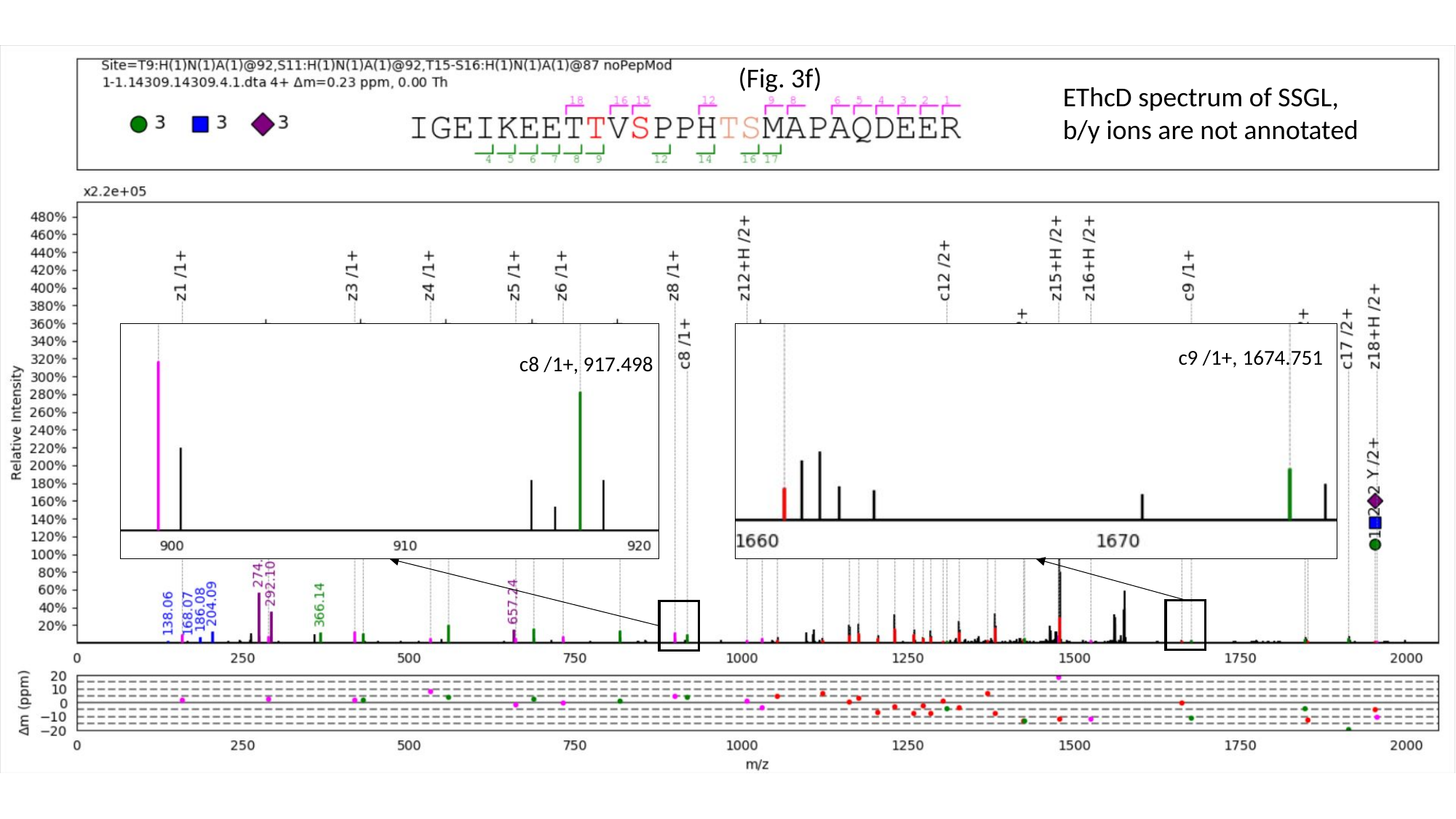

(Fig. 3f)
EThcD spectrum of SSGL, b/y ions are not annotated
c9 /1+, 1674.751
c8 /1+, 917.498

## Slide 38
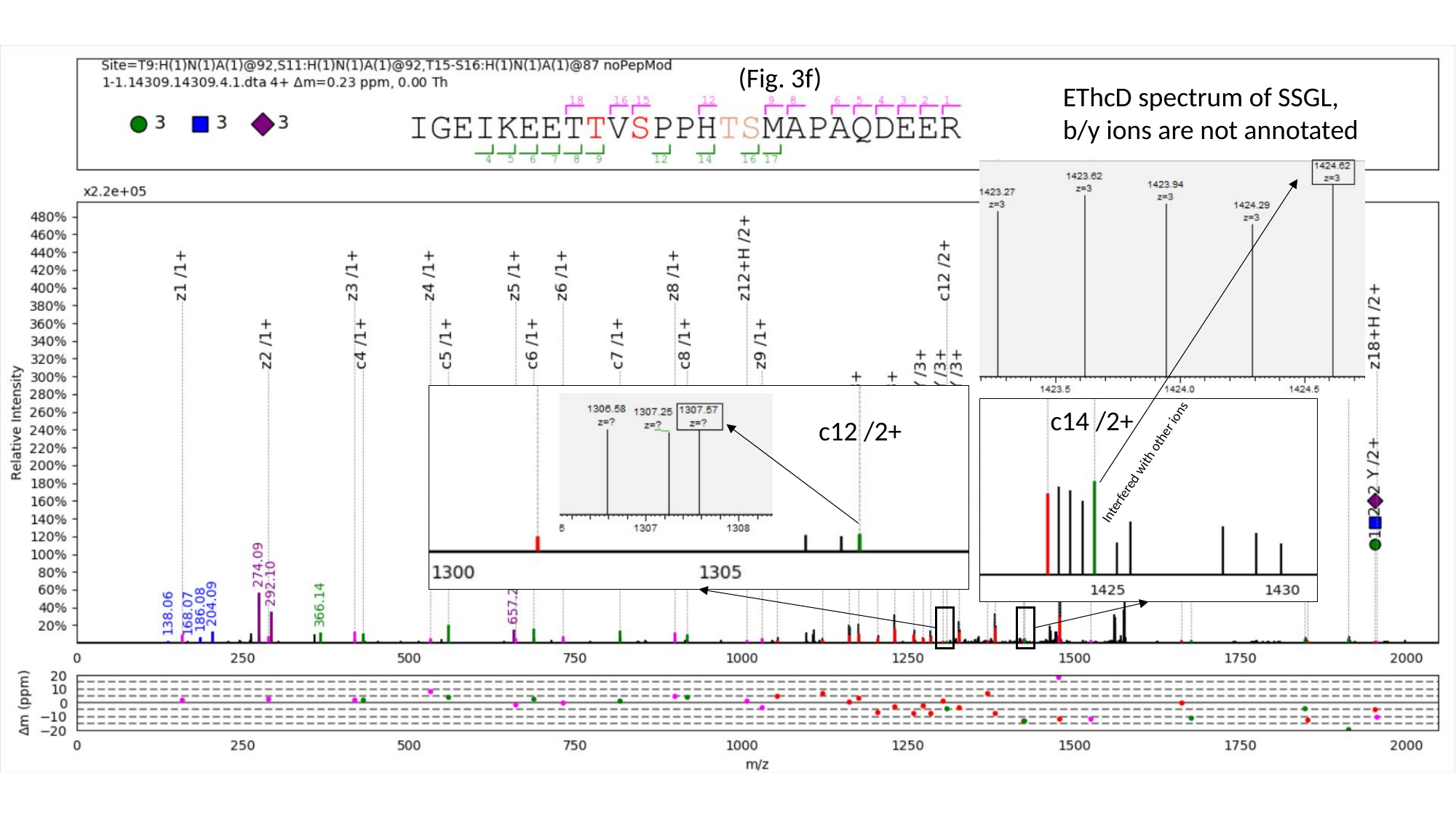

(Fig. 3f)
EThcD spectrum of SSGL, b/y ions are not annotated
c14 /2+
c12 /2+
Interfered with other ions

## Slide 39
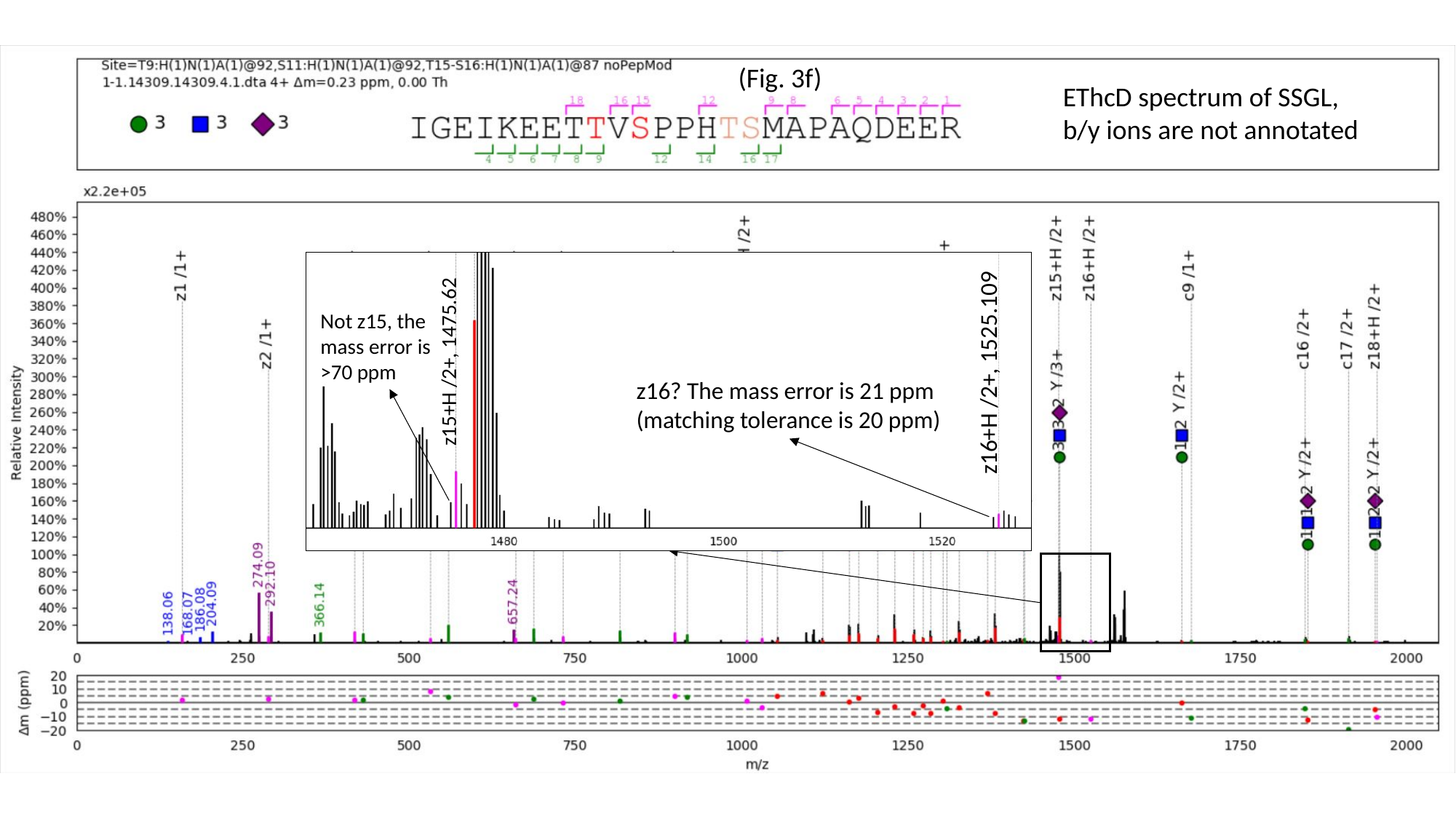

(Fig. 3f)
EThcD spectrum of SSGL, b/y ions are not annotated
Not z15, the mass error is >70 ppm
z15+H /2+, 1475.62
z16+H /2+, 1525.109
z16? The mass error is 21 ppm (matching tolerance is 20 ppm)

## Slide 40
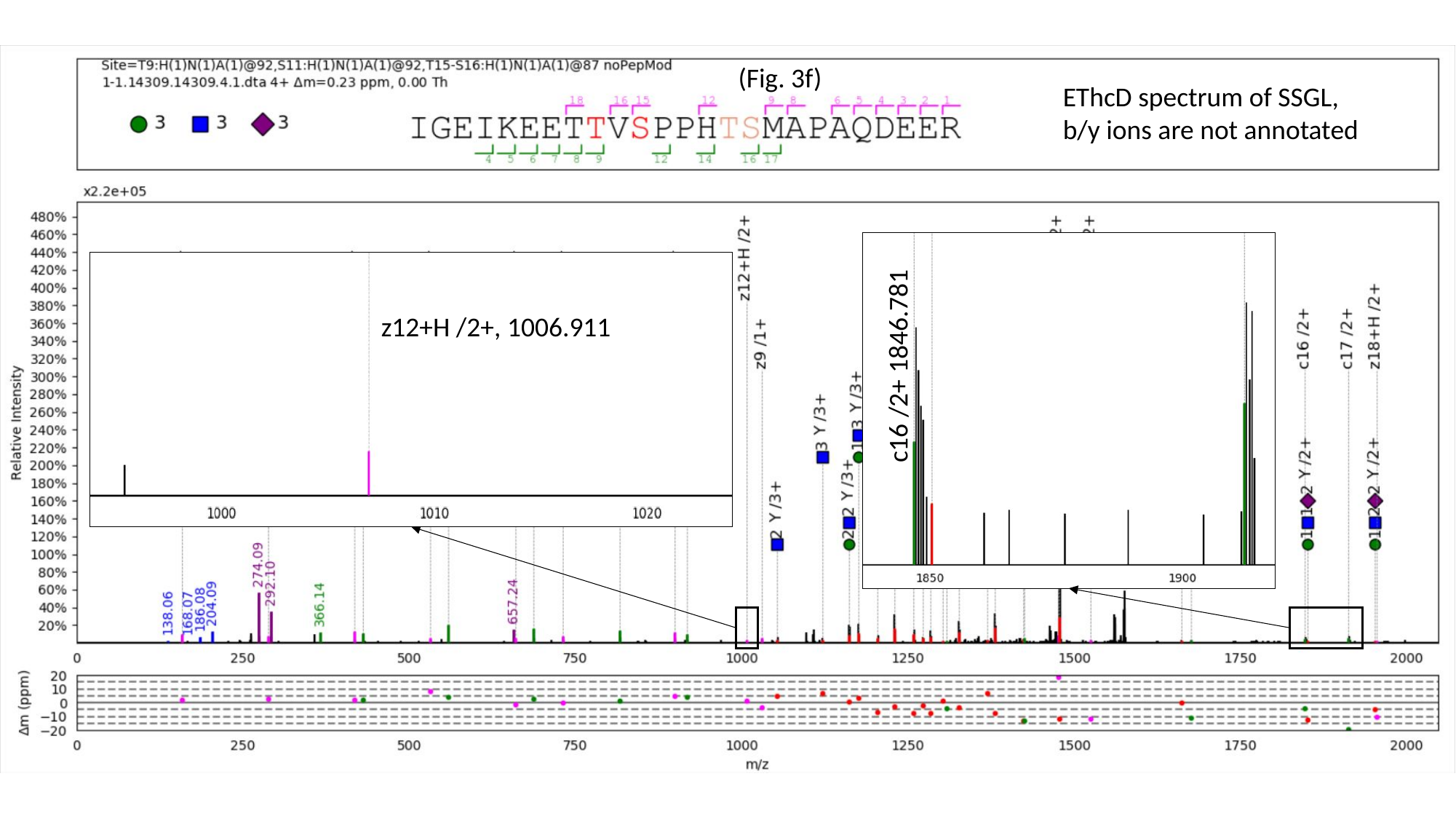

(Fig. 3f)
EThcD spectrum of SSGL, b/y ions are not annotated
z12+H /2+, 1006.911
c16 /2+ 1846.781

## Slide 41
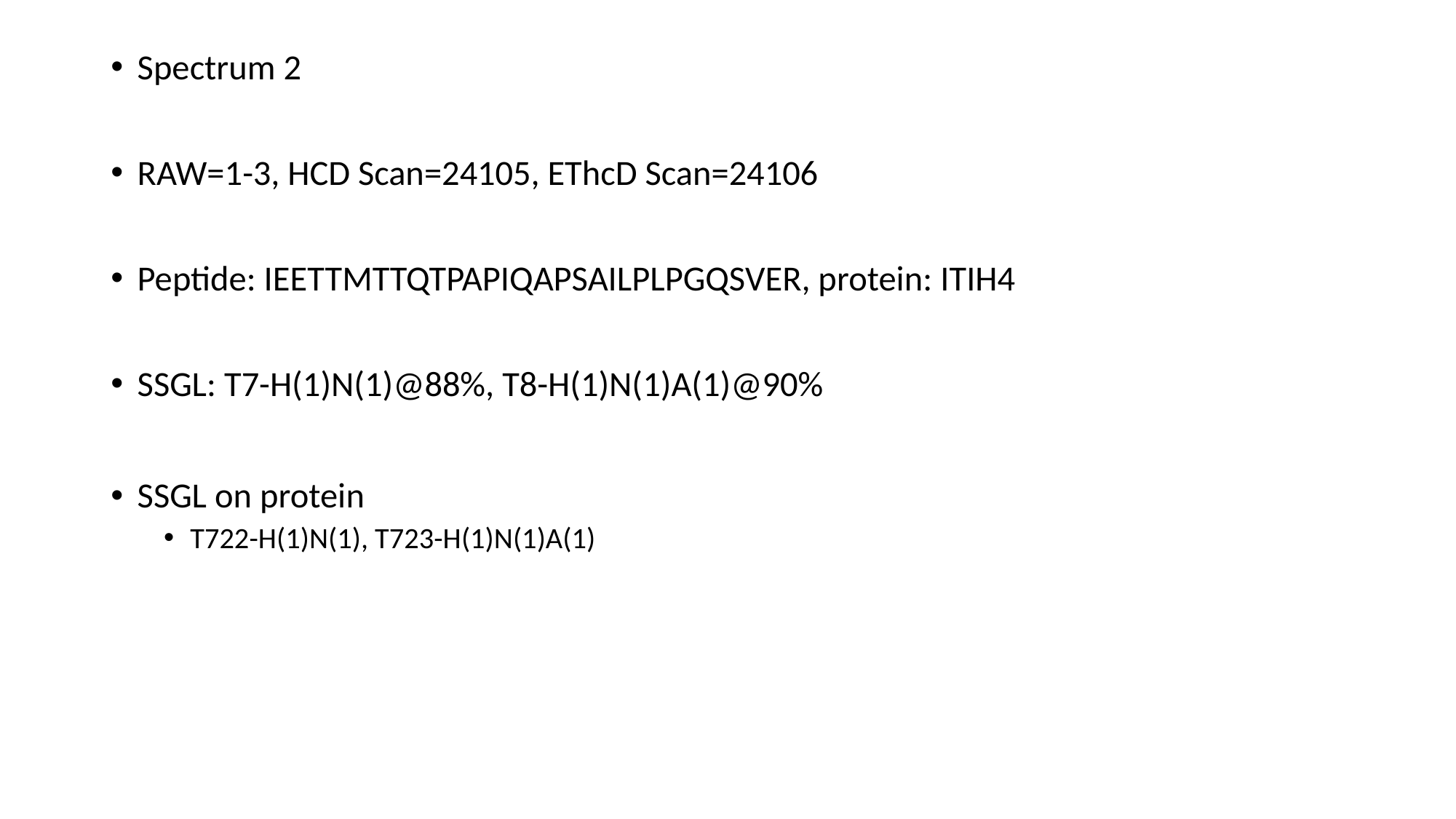

Spectrum 2
RAW=1-3, HCD Scan=24105, EThcD Scan=24106
Peptide: IEETTMTTQTPAPIQAPSAILPLPGQSVER, protein: ITIH4
SSGL: T7-H(1)N(1)@88%, T8-H(1)N(1)A(1)@90%
SSGL on protein
T722-H(1)N(1), T723-H(1)N(1)A(1)

## Slide 42
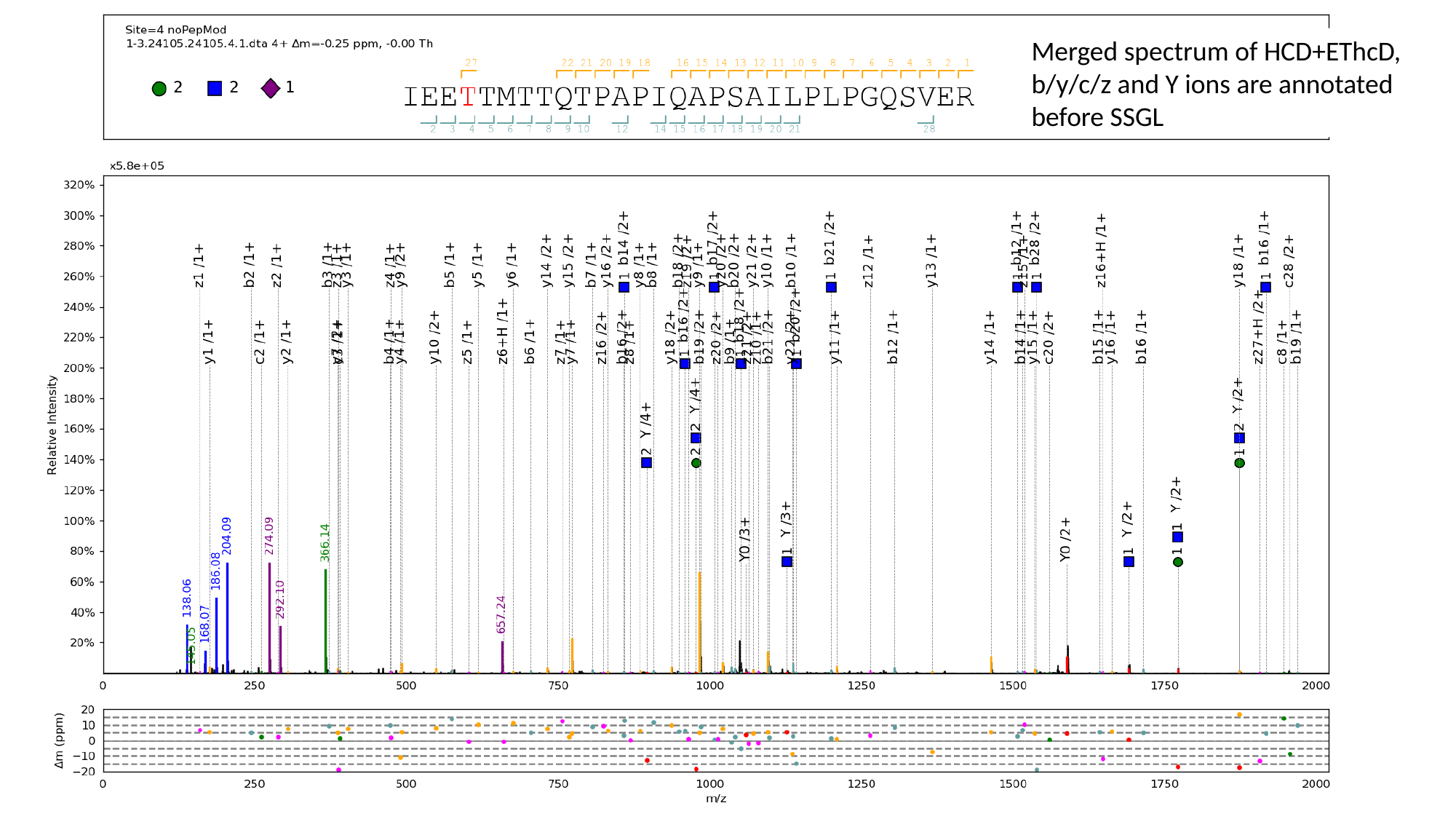

Merged spectrum of HCD+EThcD,
b/y/c/z and Y ions are annotated
before SSGL

## Slide 43
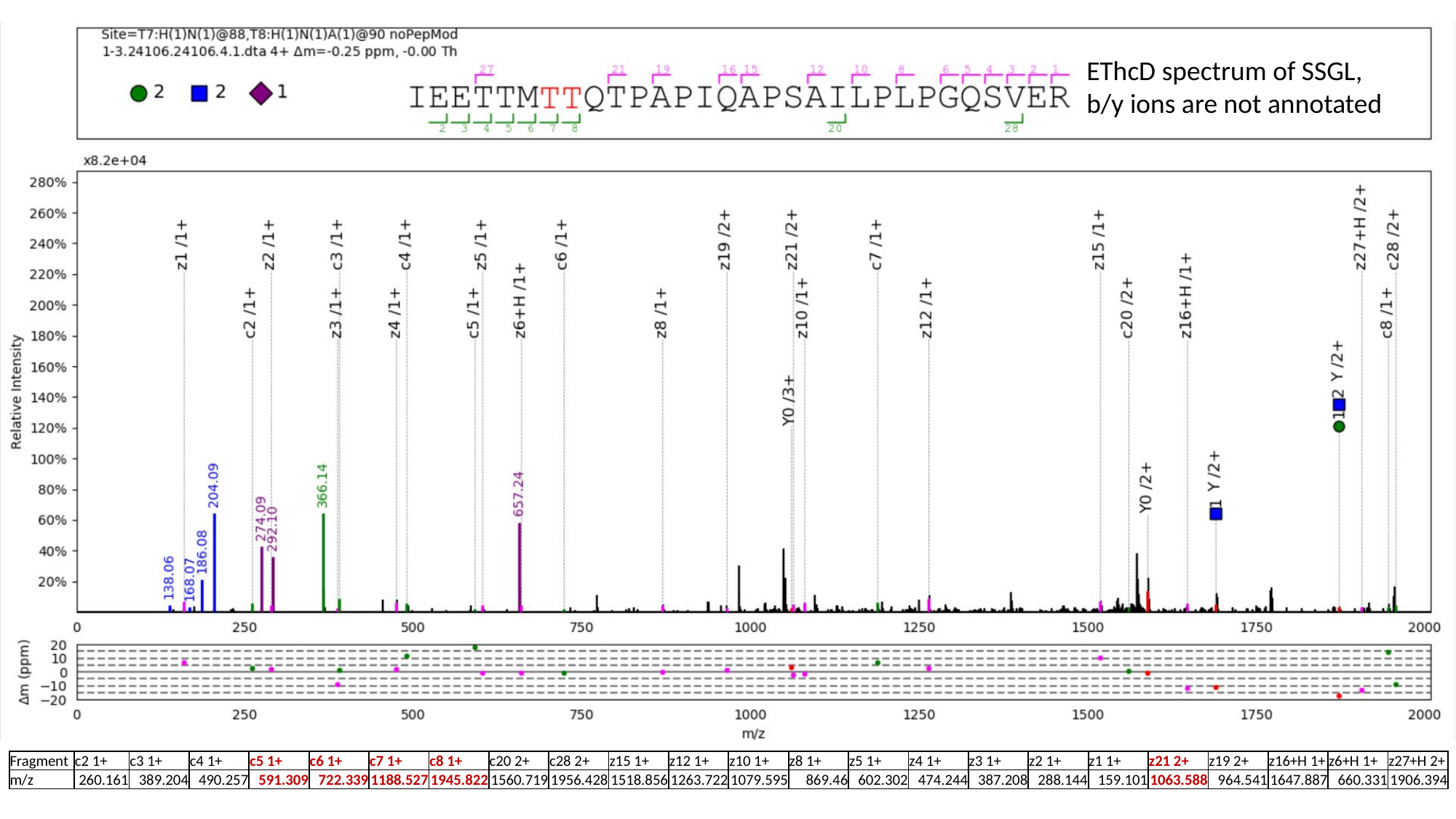

EThcD spectrum of SSGL, b/y ions are not annotated
| Fragment | c2 1+ | c3 1+ | c4 1+ | c5 1+ | c6 1+ | c7 1+ | c8 1+ | c20 2+ | c28 2+ | z15 1+ | z12 1+ | z10 1+ | z8 1+ | z5 1+ | z4 1+ | z3 1+ | z2 1+ | z1 1+ | z21 2+ | z19 2+ | z16+H 1+ | z6+H 1+ | z27+H 2+ |
| --- | --- | --- | --- | --- | --- | --- | --- | --- | --- | --- | --- | --- | --- | --- | --- | --- | --- | --- | --- | --- | --- | --- | --- |
| m/z | 260.161 | 389.204 | 490.257 | 591.309 | 722.339 | 1188.527 | 1945.822 | 1560.719 | 1956.428 | 1518.856 | 1263.722 | 1079.595 | 869.46 | 602.302 | 474.244 | 387.208 | 288.144 | 159.101 | 1063.588 | 964.541 | 1647.887 | 660.331 | 1906.394 |

## Slide 44
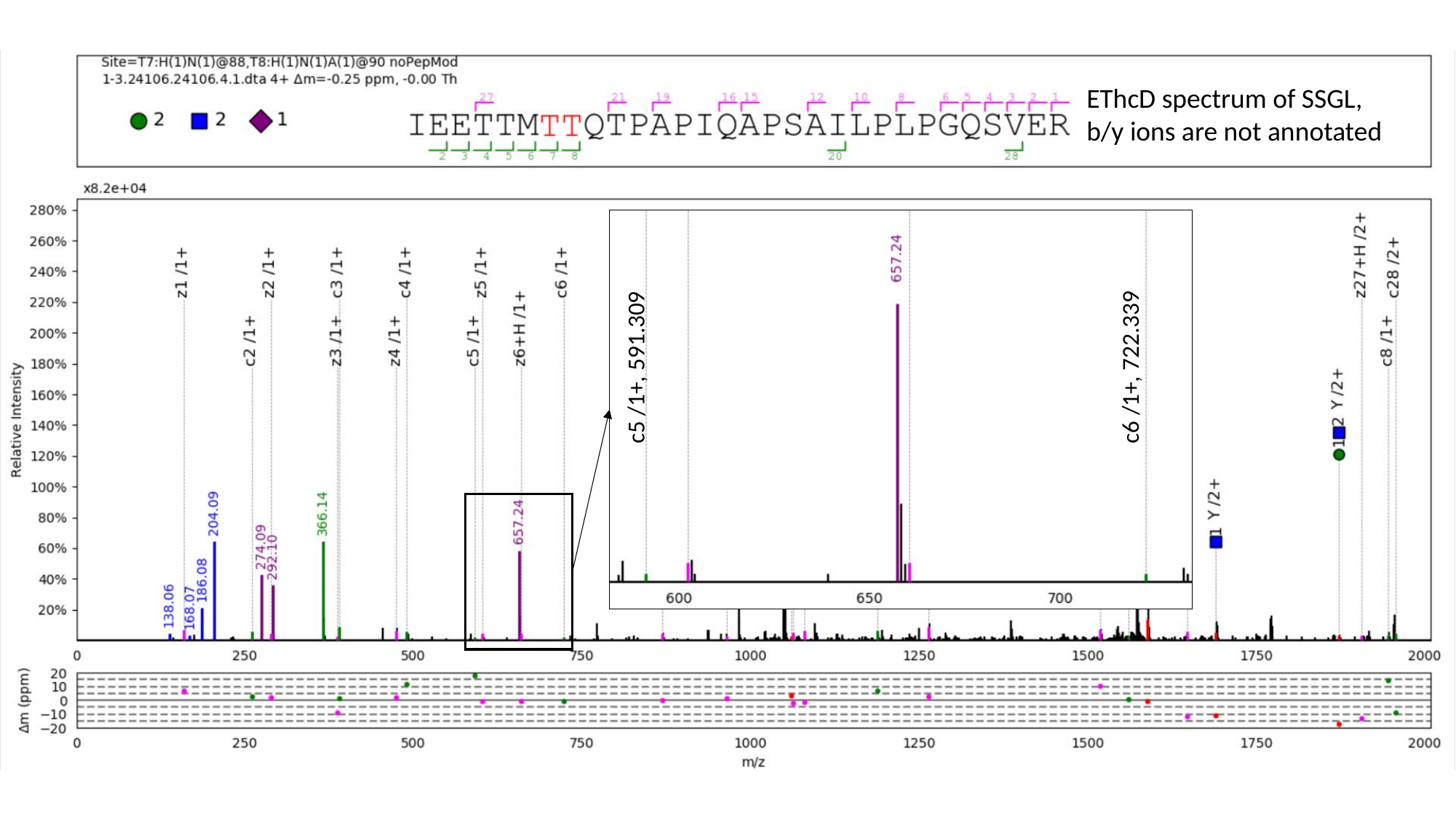

EThcD spectrum of SSGL, b/y ions are not annotated
c6 /1+, 722.339
c5 /1+, 591.309

## Slide 45
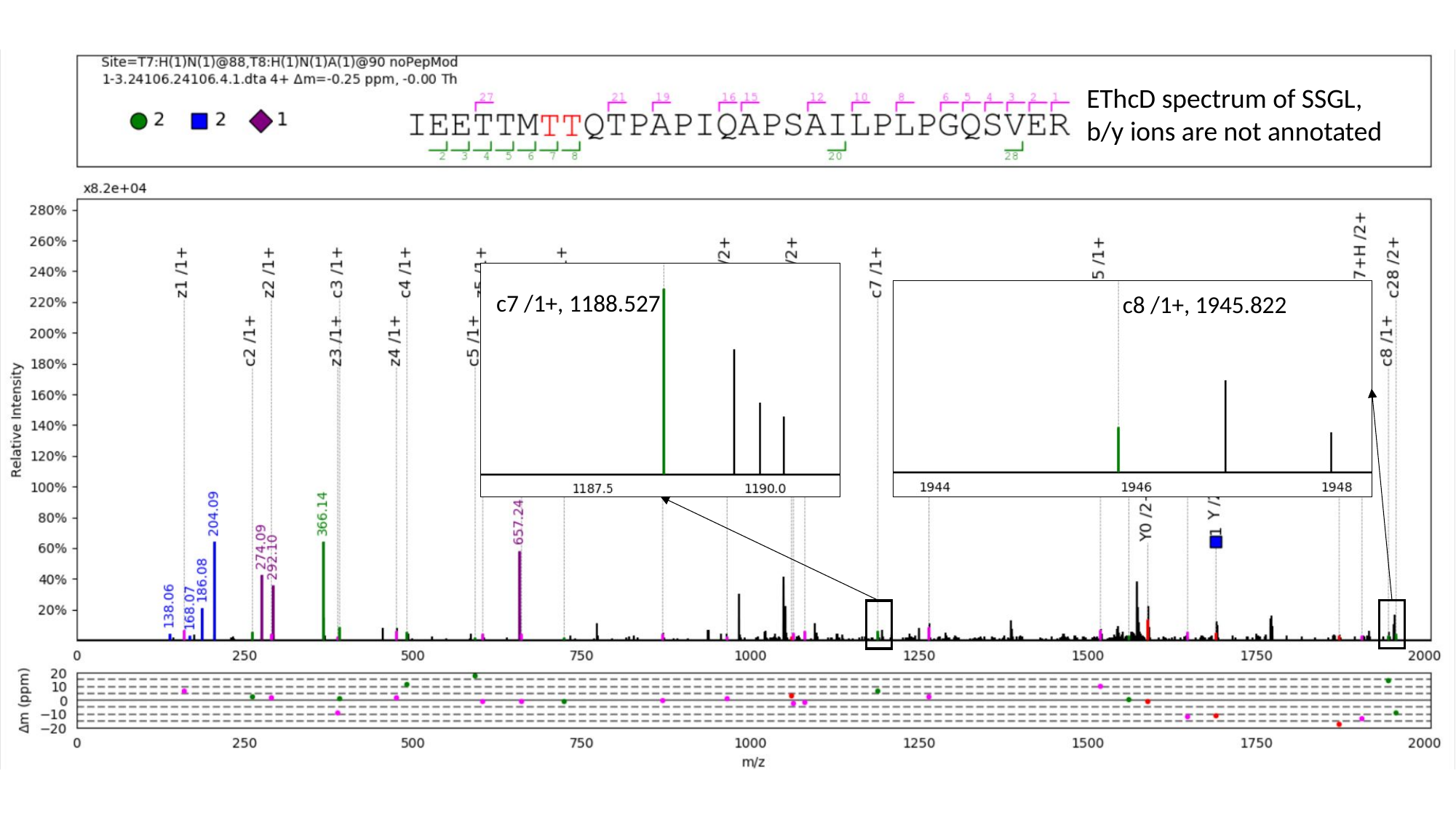

EThcD spectrum of SSGL, b/y ions are not annotated
c7 /1+, 1188.527
c8 /1+, 1945.822

## Slide 46
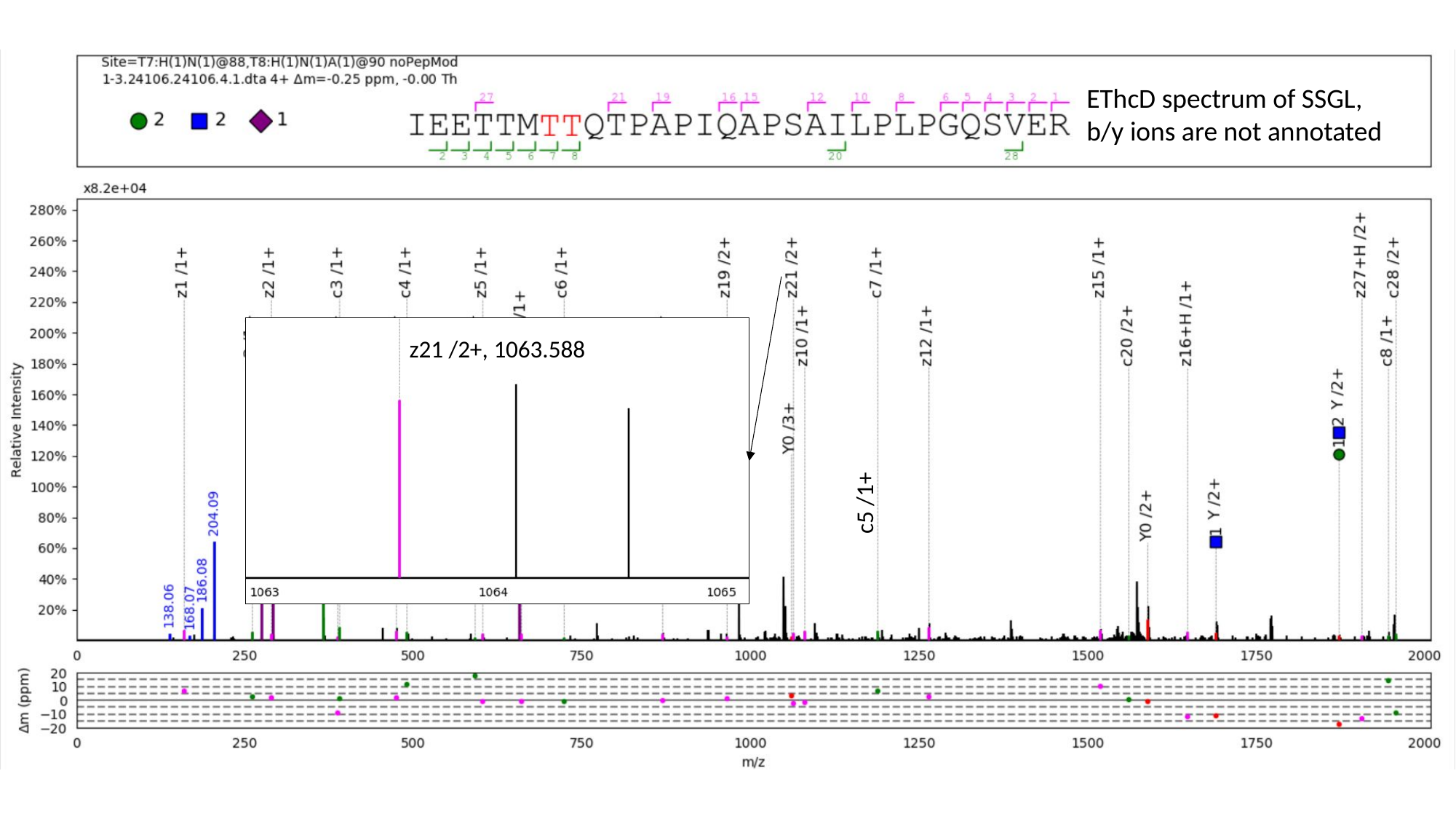

EThcD spectrum of SSGL, b/y ions are not annotated
z21 /2+, 1063.588
c5 /1+

## Slide 47
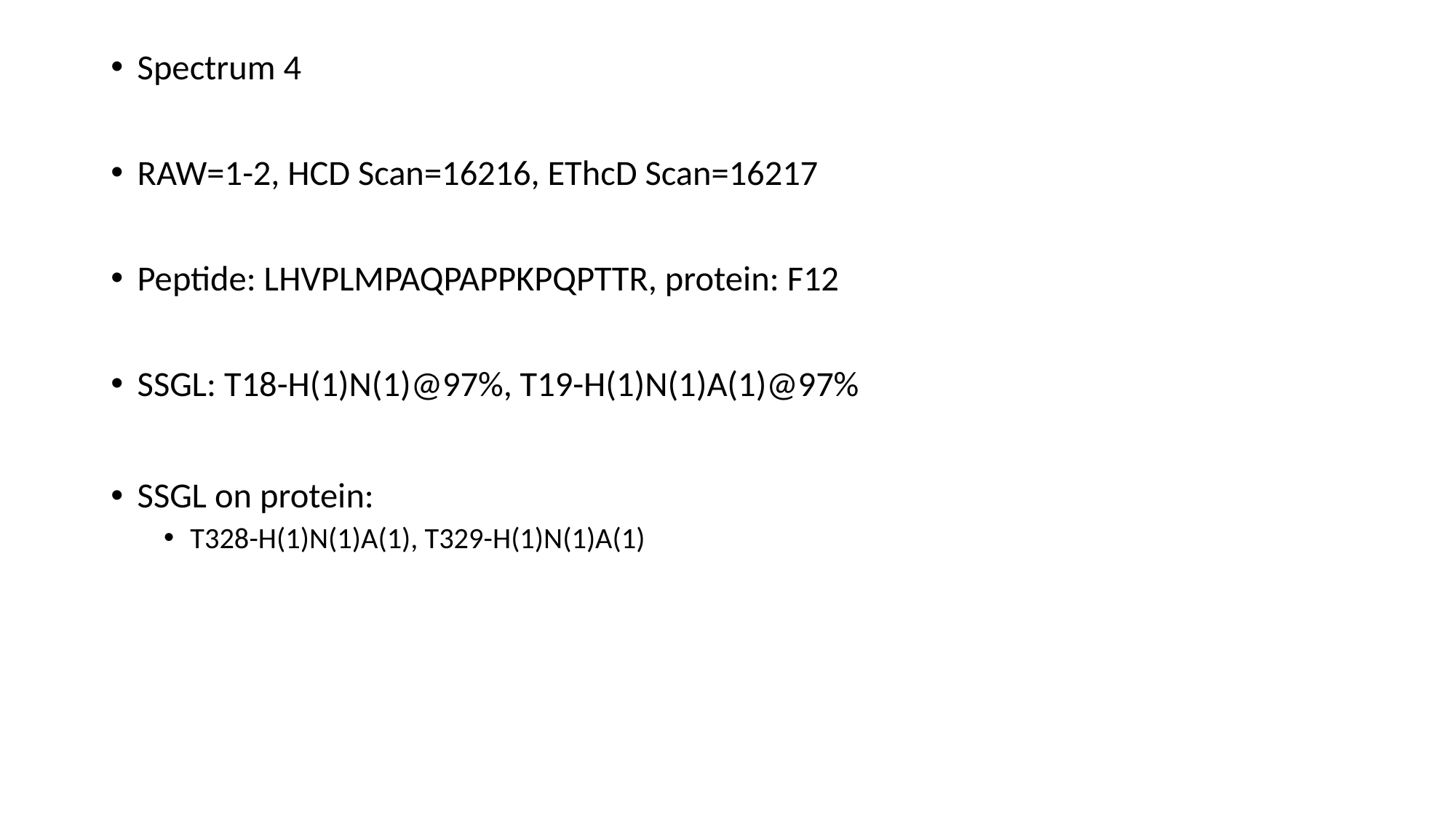

Spectrum 4
RAW=1-2, HCD Scan=16216, EThcD Scan=16217
Peptide: LHVPLMPAQPAPPKPQPTTR, protein: F12
SSGL: T18-H(1)N(1)@97%, T19-H(1)N(1)A(1)@97%
SSGL on protein:
T328-H(1)N(1)A(1), T329-H(1)N(1)A(1)

## Slide 48
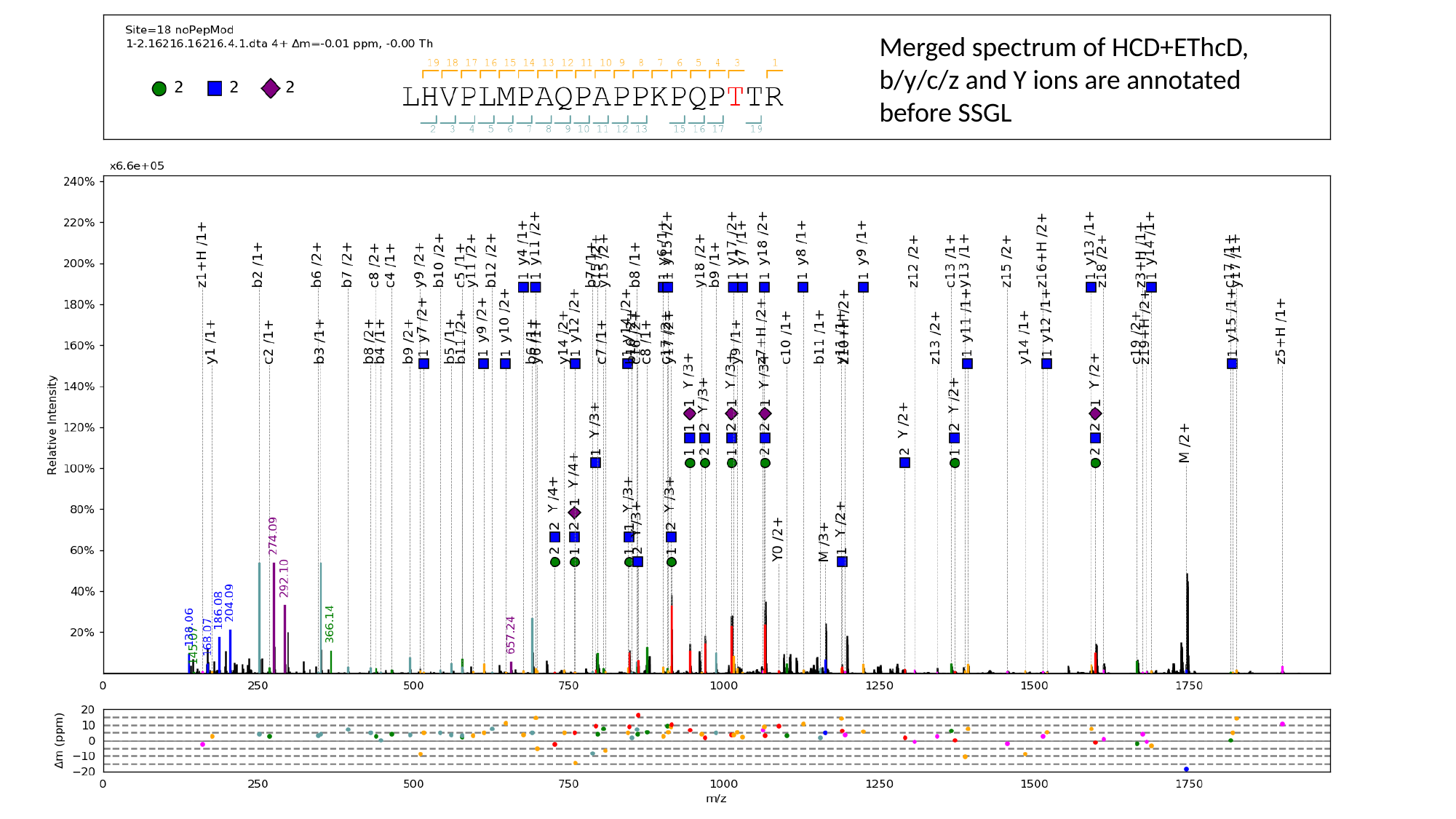

Merged spectrum of HCD+EThcD,
b/y/c/z and Y ions are annotated
before SSGL

## Slide 49
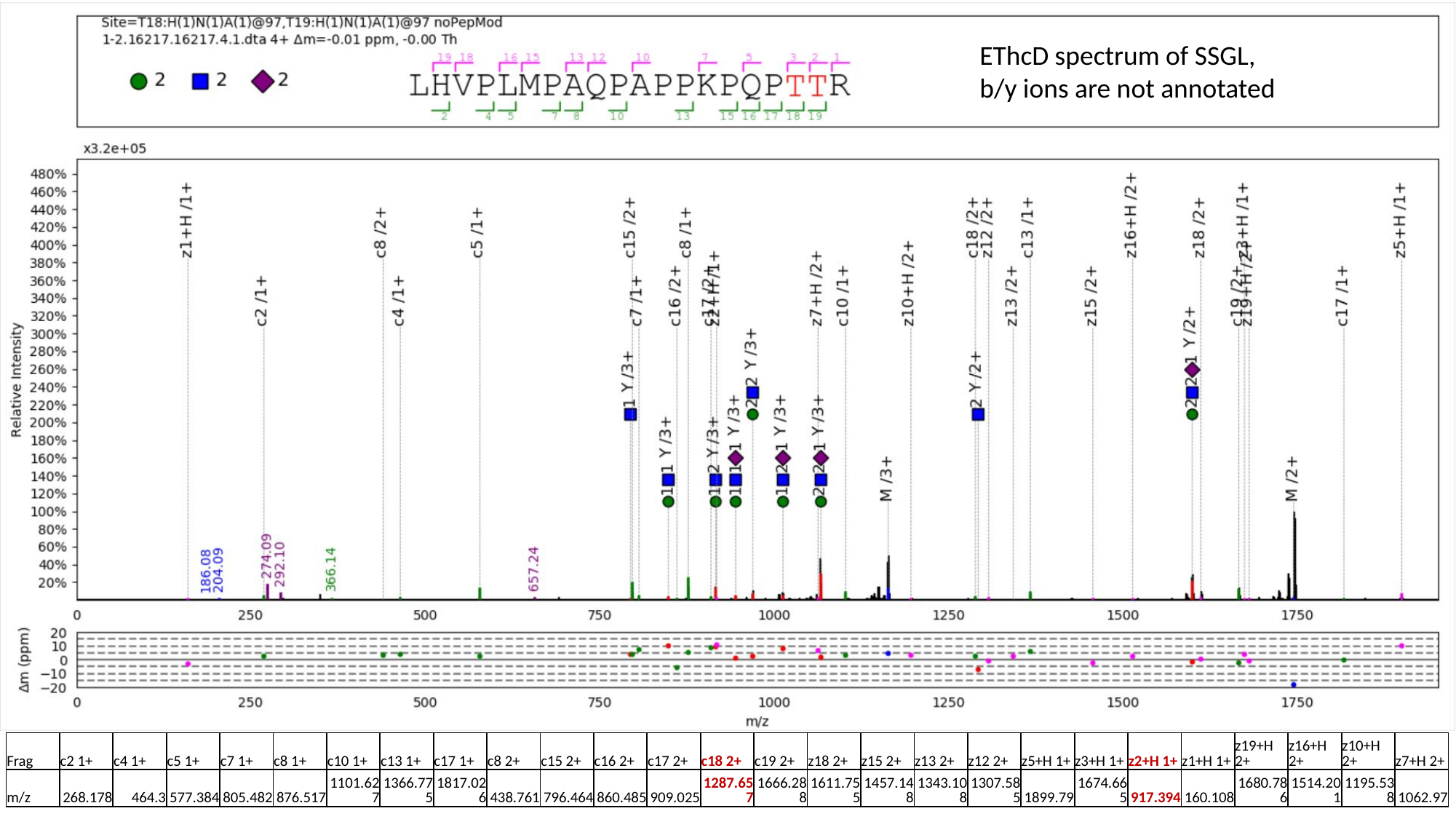

EThcD spectrum of SSGL, b/y ions are not annotated
| Frag | c2 1+ | c4 1+ | c5 1+ | c7 1+ | c8 1+ | c10 1+ | c13 1+ | c17 1+ | c8 2+ | c15 2+ | c16 2+ | c17 2+ | c18 2+ | c19 2+ | z18 2+ | z15 2+ | z13 2+ | z12 2+ | z5+H 1+ | z3+H 1+ | z2+H 1+ | z1+H 1+ | z19+H 2+ | z16+H 2+ | z10+H 2+ | z7+H 2+ |
| --- | --- | --- | --- | --- | --- | --- | --- | --- | --- | --- | --- | --- | --- | --- | --- | --- | --- | --- | --- | --- | --- | --- | --- | --- | --- | --- |
| m/z | 268.178 | 464.3 | 577.384 | 805.482 | 876.517 | 1101.627 | 1366.775 | 1817.026 | 438.761 | 796.464 | 860.485 | 909.025 | 1287.657 | 1666.288 | 1611.755 | 1457.148 | 1343.108 | 1307.585 | 1899.79 | 1674.665 | 917.394 | 160.108 | 1680.786 | 1514.201 | 1195.538 | 1062.97 |

## Slide 50
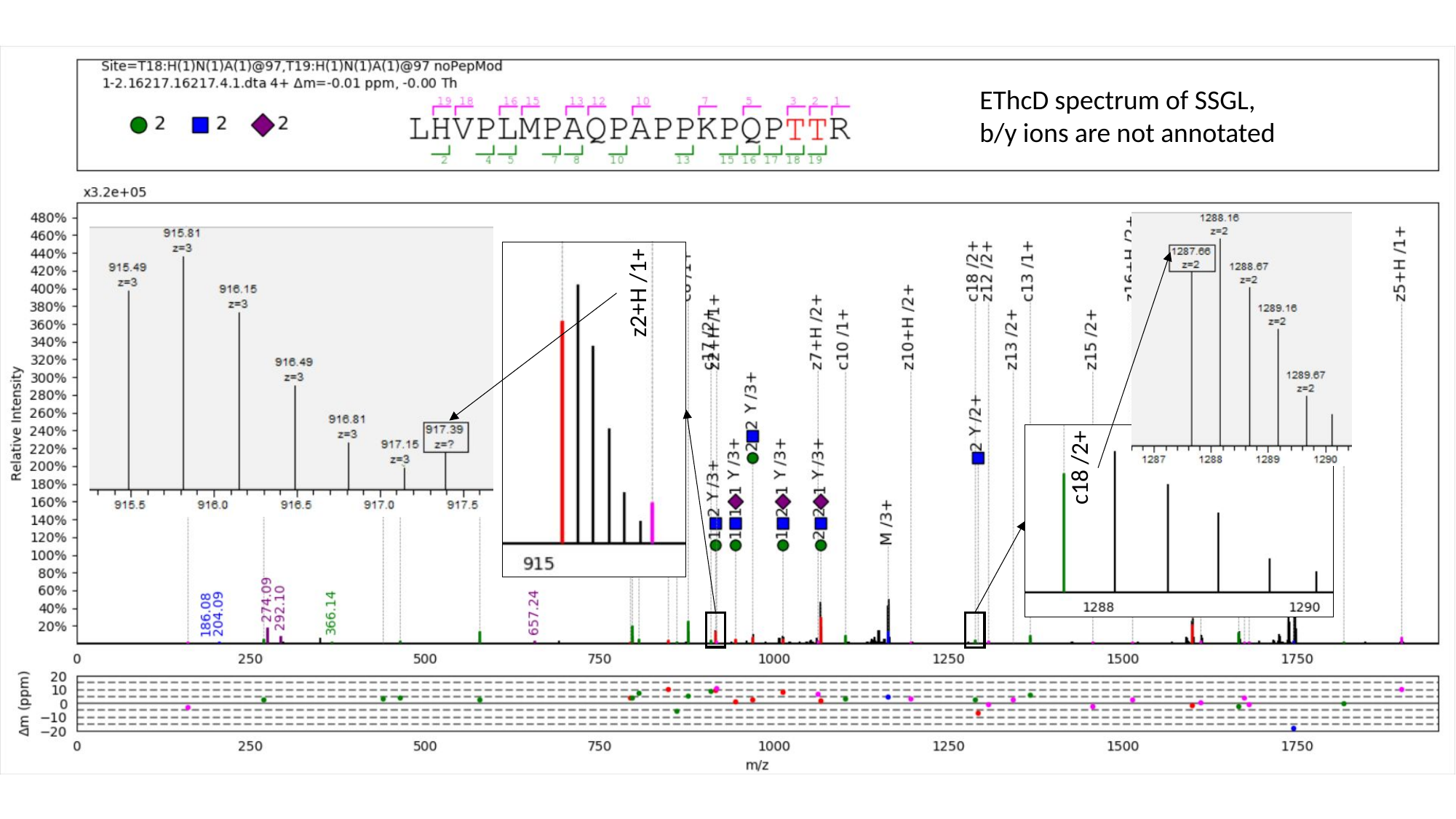

EThcD spectrum of SSGL, b/y ions are not annotated
z2+H /1+
c18 /2+

## Slide 51
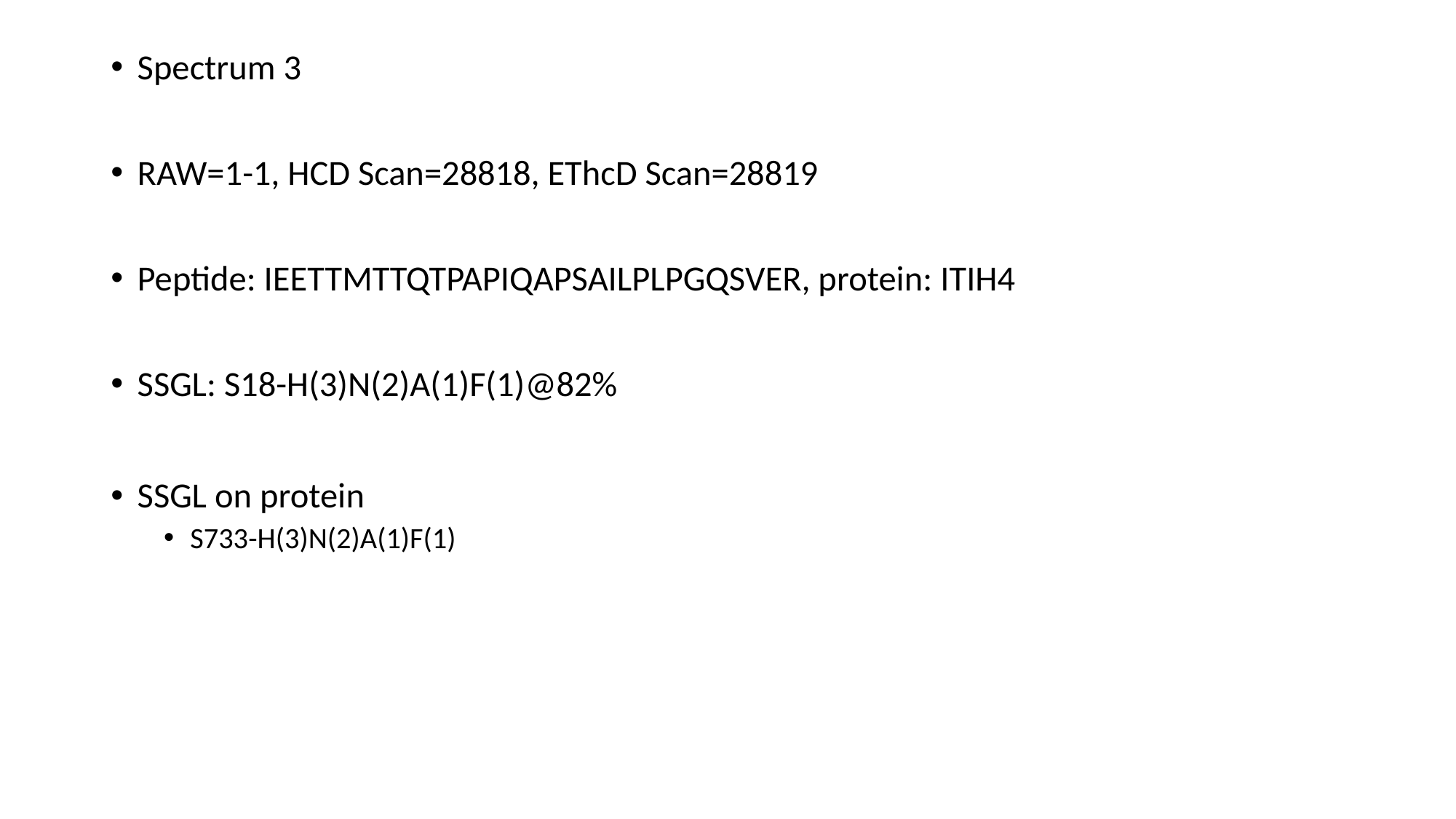

Spectrum 3
RAW=1-1, HCD Scan=28818, EThcD Scan=28819
Peptide: IEETTMTTQTPAPIQAPSAILPLPGQSVER, protein: ITIH4
SSGL: S18-H(3)N(2)A(1)F(1)@82%
SSGL on protein
S733-H(3)N(2)A(1)F(1)

## Slide 52
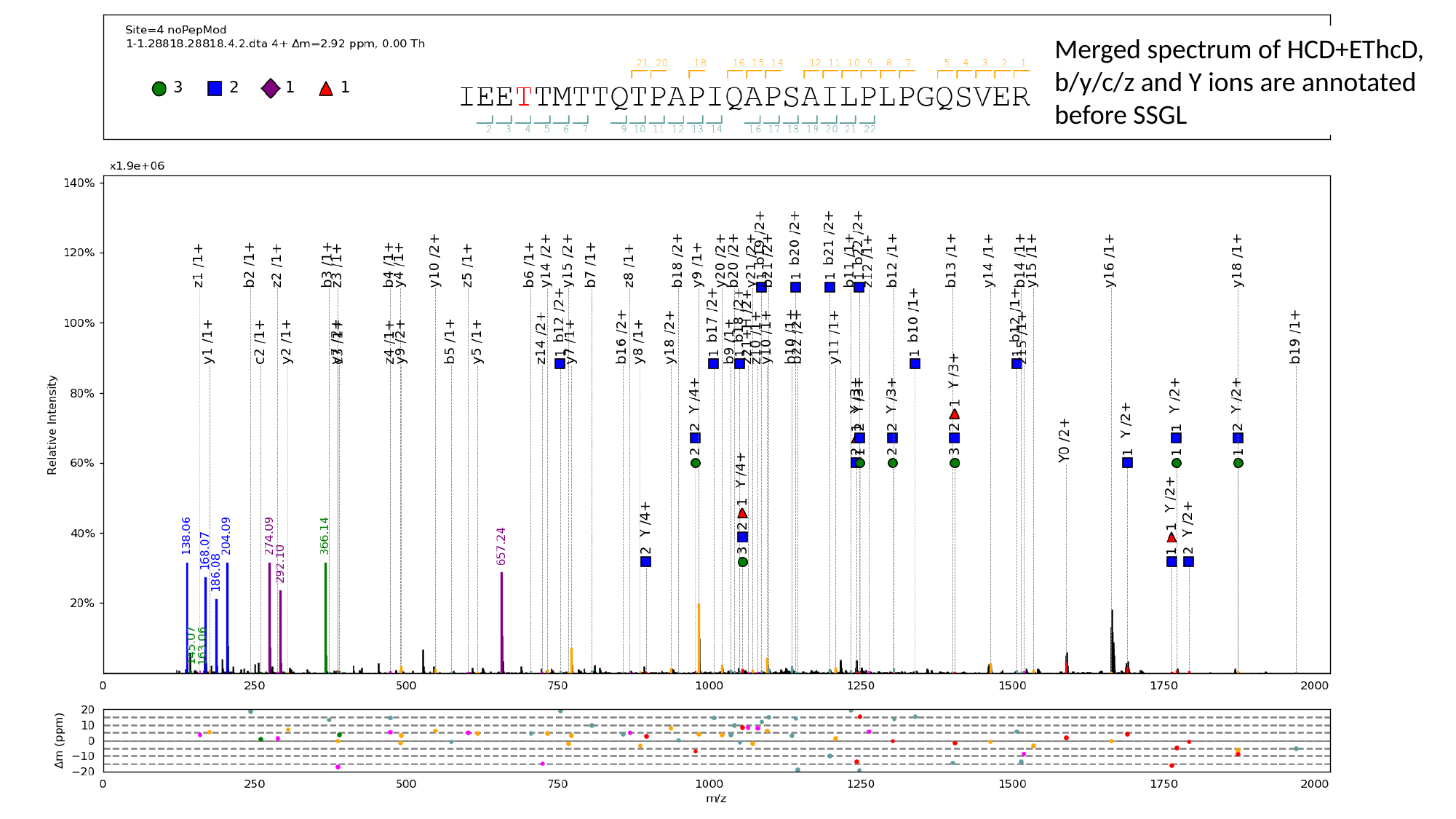

Merged spectrum of HCD+EThcD,
b/y/c/z and Y ions are annotated
before SSGL

## Slide 53
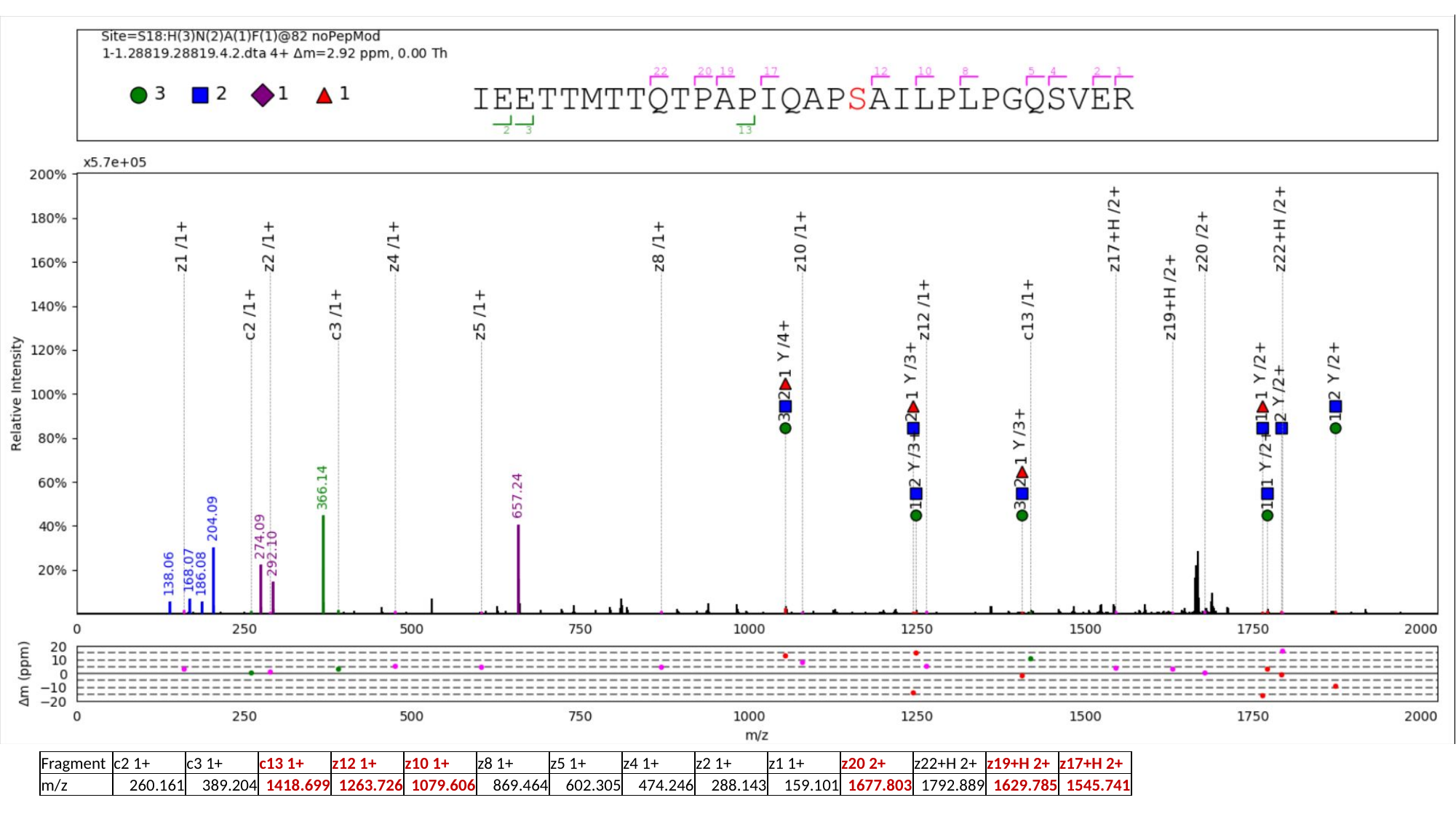

| Fragment | c2 1+ | c3 1+ | c13 1+ | z12 1+ | z10 1+ | z8 1+ | z5 1+ | z4 1+ | z2 1+ | z1 1+ | z20 2+ | z22+H 2+ | z19+H 2+ | z17+H 2+ |
| --- | --- | --- | --- | --- | --- | --- | --- | --- | --- | --- | --- | --- | --- | --- |
| m/z | 260.161 | 389.204 | 1418.699 | 1263.726 | 1079.606 | 869.464 | 602.305 | 474.246 | 288.143 | 159.101 | 1677.803 | 1792.889 | 1629.785 | 1545.741 |

## Slide 54
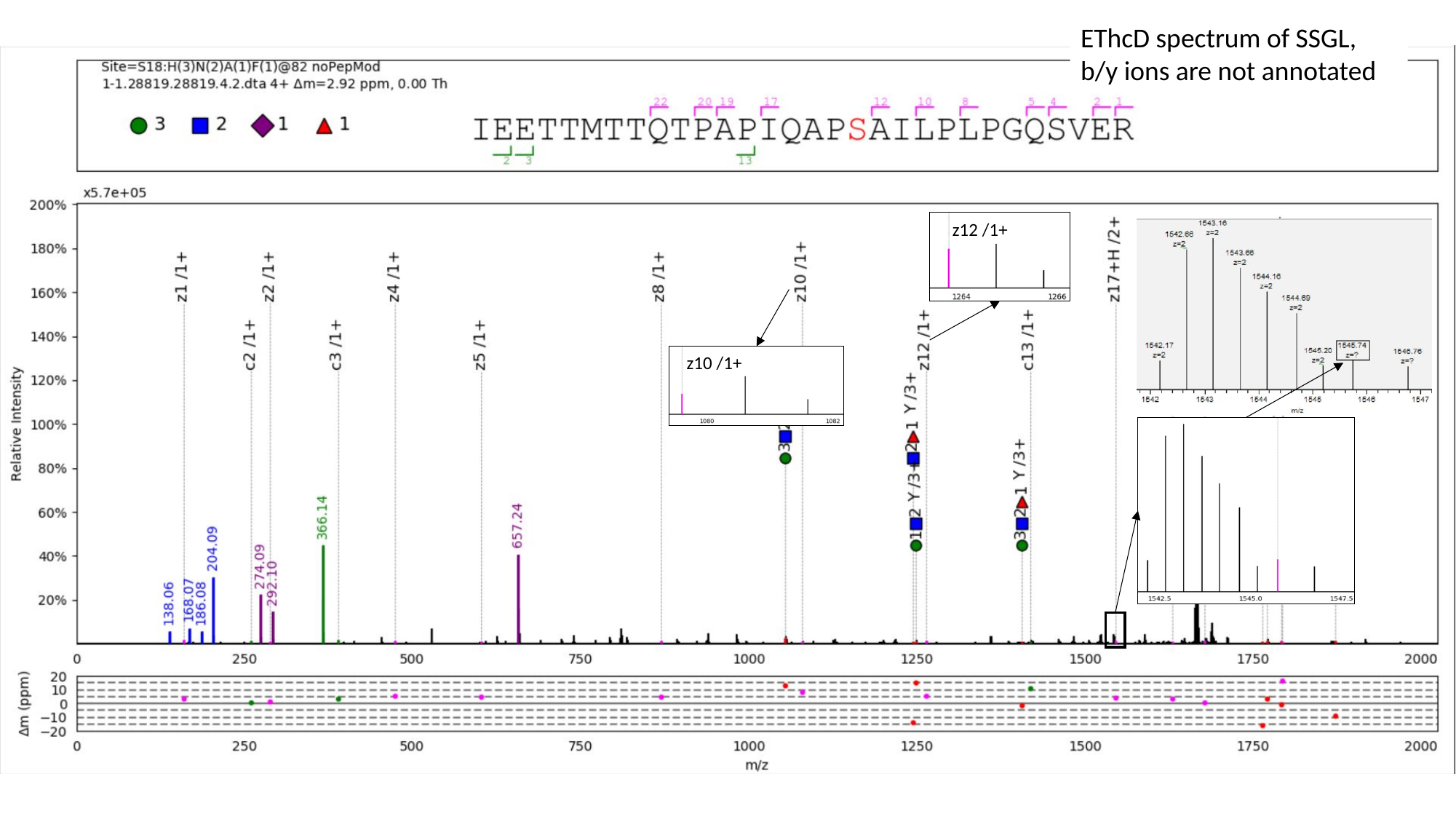

EThcD spectrum of SSGL, b/y ions are not annotated
z12 /1+
z10 /1+

## Slide 55
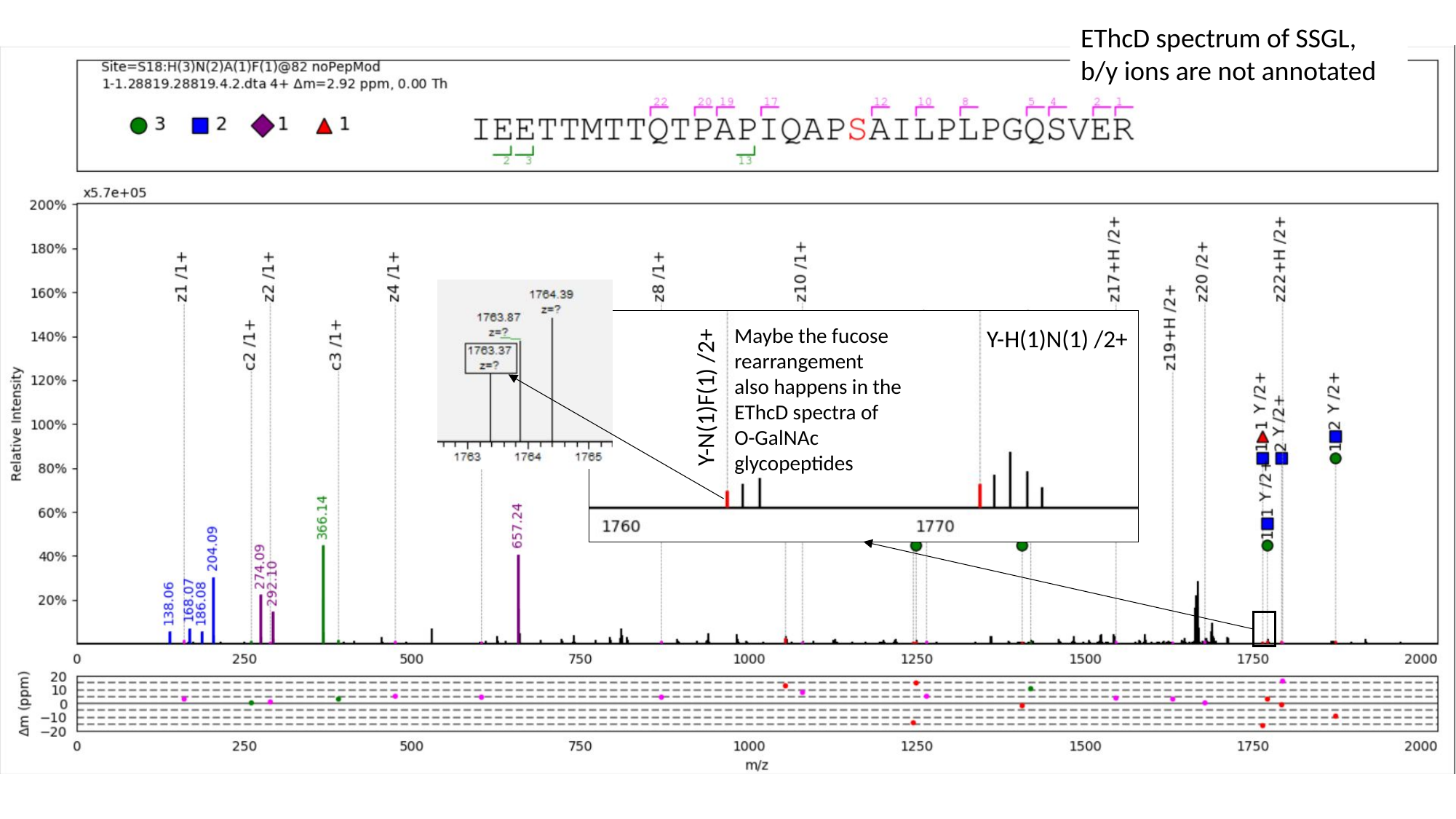

EThcD spectrum of SSGL, b/y ions are not annotated
Maybe the fucose rearrangement also happens in the EThcD spectra of O-GalNAc glycopeptides
Y-H(1)N(1) /2+
Y-N(1)F(1) /2+

## Slide 56
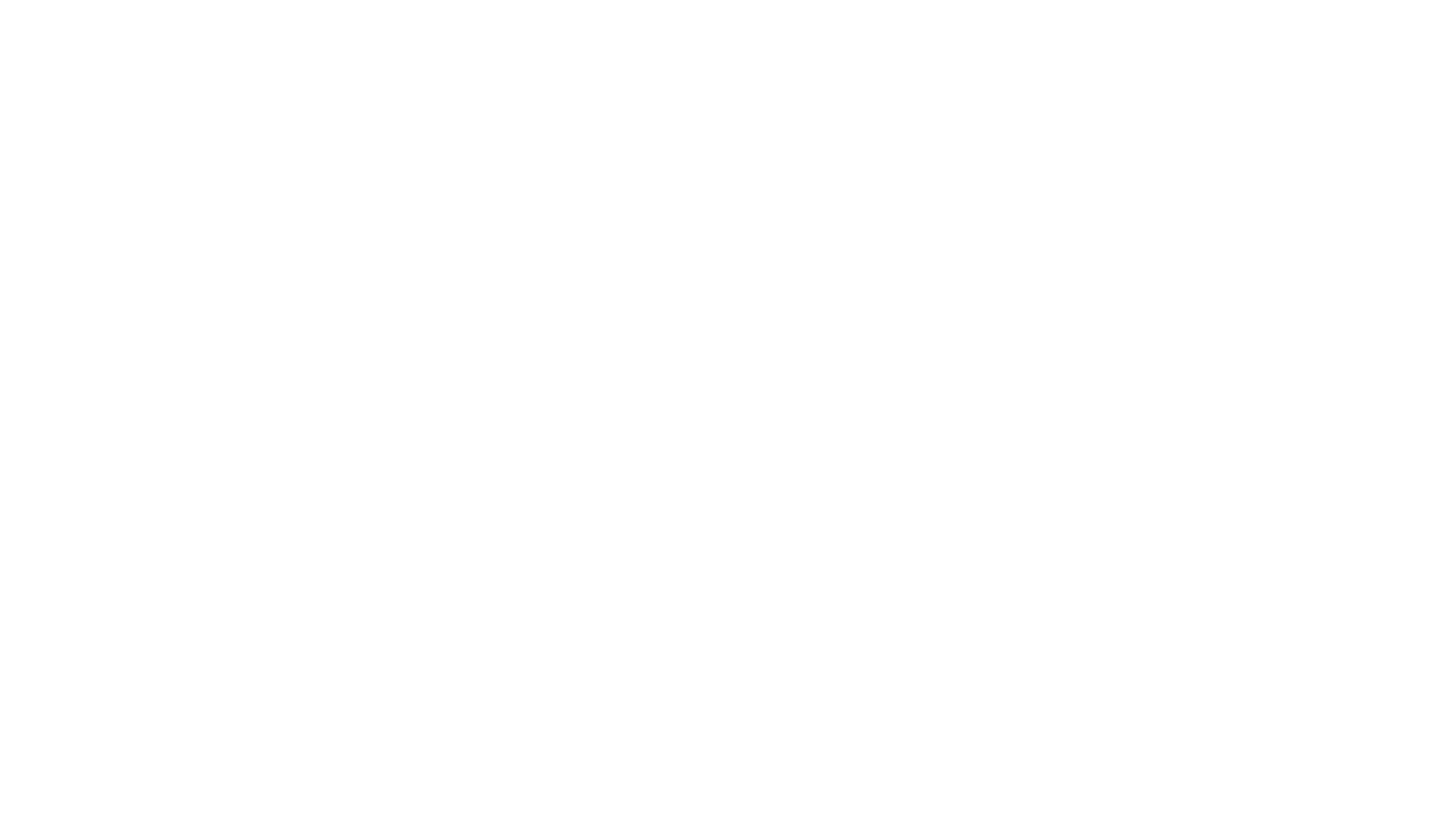

Supplement: Supplementary file 3 — All additional data files. [file 41592_2021_1306_MOESM3_ESM.zip › Supplementary Data/Spectrum Annotations.pptx]
